# Hydroboration of Terminal Alkenes and *trans*‐1,2‐Diboration of Terminal Alkynes Catalyzed by a Manganese(I) Alkyl Complex

**DOI:** 10.1002/anie.202110736

**Published:** 2021-10-13

**Authors:** Stefan Weber, Daniel Zobernig, Berthold Stöger, Luis F. Veiros, Karl Kirchner

**Affiliations:** ^1^ Institute of Applied Synthetic Chemistry Vienna University of Technology Getreidemarkt 9/163-AC A-1060 Wien Austria; ^2^ X-Ray Center Vienna University of Technology Getreidemarkt 9 A-1060 Wien Austria; ^3^ Centro de Química Estrutural and Departamento de Engenharia Química Instituto Superior Técnico Universidade de Lisboa Av Rovisco Pais 1049-001 Lisboa Portugal

**Keywords:** 1,2-diboration, alkenes, alkynes, hydroboration, manganese alkyl complex

## Abstract

A Mn^I^‐catalyzed hydroboration of terminal alkenes and a 1,2‐diboration of terminal alkynes with pinacolborane (HBPin) is described. For alkenes, anti‐Markovnikov hydroboration takes place; for alkynes the reaction proceeds with excellent trans‐1,2‐selectivity. The most active pre‐catalyst is bench‐stable alkyl bisphosphine Mn^I^ complex fac‐[Mn(dippe)(CO)_3_(CH_2_CH_2_CH_3_)]. The catalytic process is initiated by migratory insertion of a CO ligand into the Mn–alkyl bond to yield an acyl intermediate, which undergoes B−H bond cleavage of HBPin (for alkenes) and rapid C−H bond cleavage (for alkynes), forming the active Mn^I^ boryl and acetylide catalysts [Mn(dippe)(CO)_2_(BPin)] and [Mn(dippe)(CO)_2_(C≡CR)], respectively. A broad variety of aromatic and aliphatic alkenes and alkynes was efficiently and selectively borylated. Mechanistic insights are provided based on experimental data and DFT calculations revealing that an acceptorless reaction is operating involving dihydrogen release.

## Graphical abstract

A Mn^I^‐catalyzed hydroboration of terminal alkenes and the 1,2‐diboration of terminal alkynes with pinacolborane is described. In the case of alkenes *anti*‐Markovnikov hydroboration takes place, while in the case of alkynes the reaction proceeds with *trans*‐1,2‐selectivity.

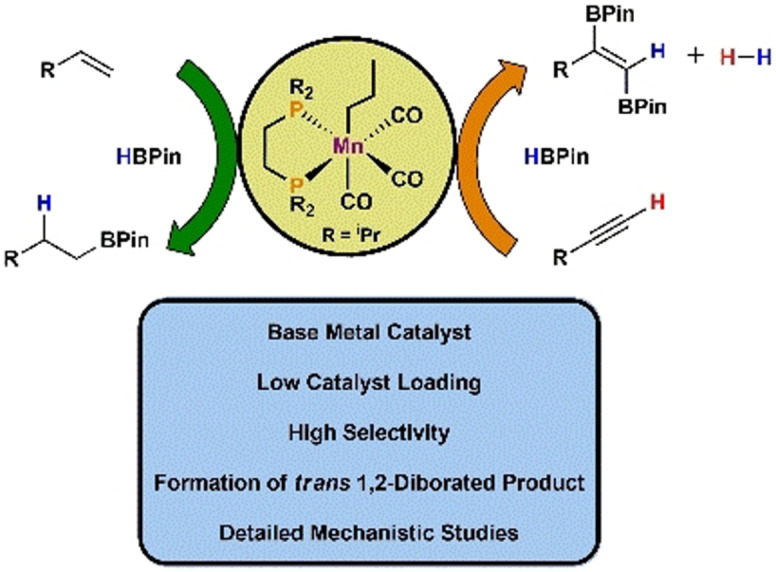

The use of organoboron reagents especially in the field of cross‐coupling chemistry increased within the last decades.^[1]^ Hydroboration reactions display a powerful tool for the synthesis of organoboron compounds.^[2]^ In the last years, the use of dialkoxyboranes such as pinacolborane (HBPin) was implemented in organic synthesis due to the stability of HBPin as reagent and convenient handling of the hydroborated product.^[3]^ However, due to the superior stability of dialkoxyboranes in comparison to “BH_3_”‐species, low reactivity towards hydroboration of alkenes is attributed.^[4]^ Within this context the transition‐metal catalyzed hydroboration of C−C multiple bonds displays a versatile route towards organoboron species.^[5]^ Catalysts with noble metals such as Rh^[6]^ and Ir^[7]^ are already well established in the field of C−C multiple bond hydroboration. In the last years, non‐precious metal catalysts based on Cu,^[8]^ Ni,^[9]^ Co^[10]^ and Fe^[11]^ were successfully introduced in this area. As manganese is concerned, several examples for Mn^I^‐catalyzed hydroborations of polarized C‐X multiple bonds such as carbonyls,^[12]^ nitriles,^[13]^ and CO_2_ ^[14]^ were reported. Interestingly, the hydroboration of alkenes^[15]^ and alkynes^[16]^ is as yet restricted to manganese complexes in the oxidation state +II containing potentially non‐innocent ligands such as terpyridine or benzylic imines. An overview of manganese‐based hydroboration catalysts for alkenes and alkynes is depicted in Scheme 1.

**Scheme 1:**
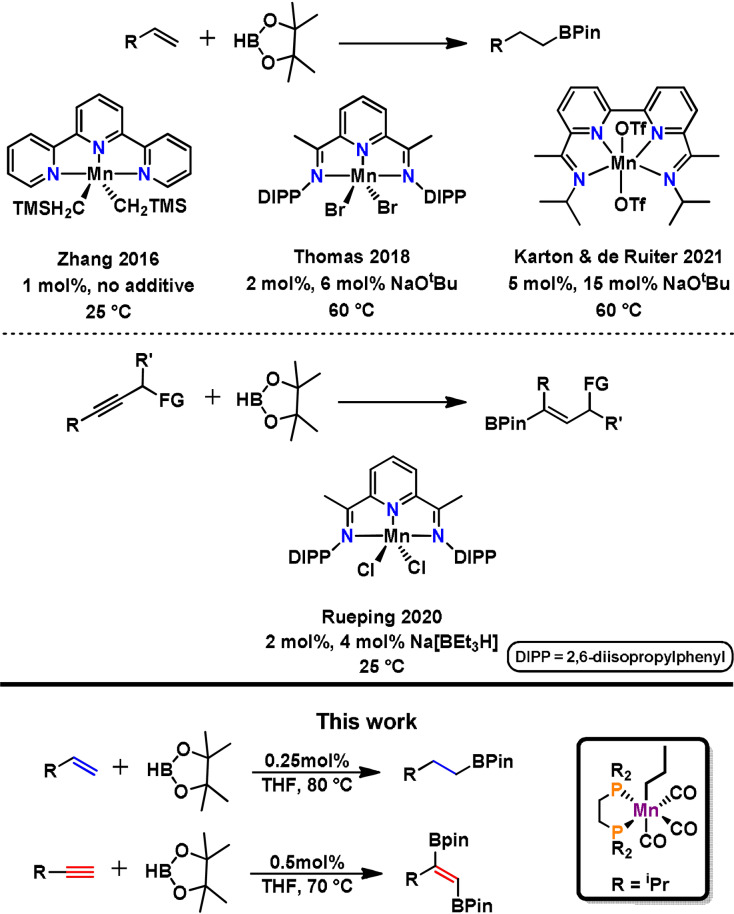
Overview of manganese‐based hydroboration catalysts.

Our group recently reported additive‐free hydrogenation of nitriles,^[17]^ alkenes,^[18]^ ketones,^[19]^ CO_2_,^[20]^ the dimerization and cross coupling of terminal alkynes,^[21]^ and dehydrogenative silylation of alkenes^[22]^ catalyzed by bench stable Mn^I^ alkyl carbonyl complexes. In contrast to the vast majority of reported Mn^I^ complexes,^[23]^ these newly introduced systems operate via an inner‐sphere mechanism.

We hereby took advantage of the fact that alkyl ligands undergo migratory insertion reactions. This leads to the formation of a complex, containing a strongly basic acyl ligand. If the entering substrate contains a weakly polarized E−H bond (E=H, C≡CR, SiR_3_, BR_2_), the acyl ligand is capable of initiating E−H bond cleavage thereby forming the catalytically Mn‐E species and a weakly bonded aldehyde ligand which can be easily substituted by incoming substrates (Scheme 2).

**Scheme 2:**
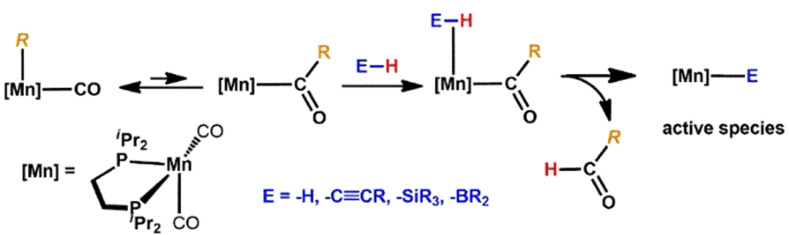
Formation of the active species via migratory insertion and deprotonation of the entering ligand.

Here, we describe the activity of *fac*‐[Mn(dippe)(CO)_3_(CH_2_CH_2_CH_3_)] (dippe=1,2‐bis(di‐*iso*‐propylphosphinoethane) (**1**), *fac*‐[Mn(dippe)(CO)_3_(Br)] (**2**) and *fac*‐[Mn(dippe)(CO)_3_(H)] (**3**) as pre‐catalysts for the selective *anti*‐Markovnikov hydroboration of terminal alkenes and the selective *trans*‐1,2‐diboration of terminal alkynes. A plausible reaction mechanism based on detailed experimental and theoretical studies is presented.

Initial catalyst evaluation for the hydroboration of terminal alkenes was carried out with complexes **1**, **2** and **3** and 4‐chlorostyrene as model substrate and HBPin.

Selected optimization reactions are depicted in Table 1 (for details, see the Supporting Information). The screening of complexes **1**, **2** and **3** revealed excellent performance for **1**, whereas only traces of product could be detected for **2** and **3**. This proved the crucial role of the alkyl group for the catalytic performance. Complex **1** shows high reactivity in THF or toluene, but no productivity in MeOH due to rapid methanolysis of HBPin leading to massive hydrogen gas evolution. It should be noted that the selectivity for all optimization reactions was at least 96 % to the *anti*‐Markovnikov product (**4 a**). The catalyst loading could be decreased to only 0.25 mol %. Lowering the catalyst amount to 0.1 mol % still gave 76 % conversion.

**Table 1:** Optimization reaction for the manganese catalyzed hydroboration of 4‐chlorostyrene with HBPin.^[a]^ 
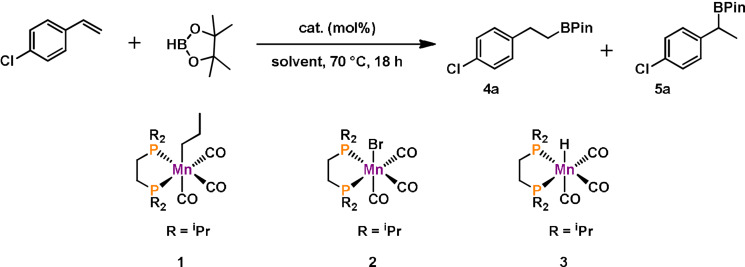

Entry	Catalyst (mol %)	Solvent	Conversion^[b]^ [%]	Ratio **4 a**:**5 a** ^[b]^
1	**1** (2)	THF	>99	97:3
2	**2** (2)	THF	2	n.d.
3	**3**(2)	THF	6	n.d.
4	**1** (1)	THF	>99	97:3
5	**1** (1)	toluene	>99	96:4
6	**1** (1)	MeOH	n.r.	n.d.
7	**1** (0.5)	THF	88	98:2
**8^[c]^**	**1 (0.25)**	**THF**	**97**	**98:2**
9^[c]^	**1** (0.1)	THF	76	99:1
10^[c]^	none	THF	7	99:1

[a] Conditions: 4‐chlorostyrene (1.13 mmol, 1 equiv), HBPin (1.15 mmol, 1.02 equiv), catalyst (0.1–2 mol %), 0.5 mL solvent, 70 °C, 18 h, Ar. [b] determined by GC‐MS analysis. [c] 80 °C, 24 h.

Having established the optimized reaction conditions, the scope and limitation of catalyst **1** was examined (Table 2). A broad variety of aryl substrates was investigated, tolerating a range of functional groups including halides, ethers, amines, and esters. Notably, the isomer ratio for all substrates was ≥98:2 towards the *anti*‐Markovnikov product. High conversions for substrates containing an electron withdrawing‐group were achieved (**4 a**–**4 c** and **4 e**–**4 g**). The investigation of aromatic systems bearing electron‐donating groups in the *para*‐position (**4 h**–**4 j**) resulted in a minor drop in reactivity. A good yield was also achieved for 2,4,6‐trimethylstyrene (**4 l**) a sterically demanding substrate, if the catalyst loading was increased to 1 mol %. Investigation of the scope for aliphatic alkenes revealed high reactivity for a large number of alkyl substrates (**4 n**–**4 v**). Negligible conversion of α‐methylstyrene (**4 w**) and no conversion of cyclohexene (**4 x**) was found.

**Table 2:**
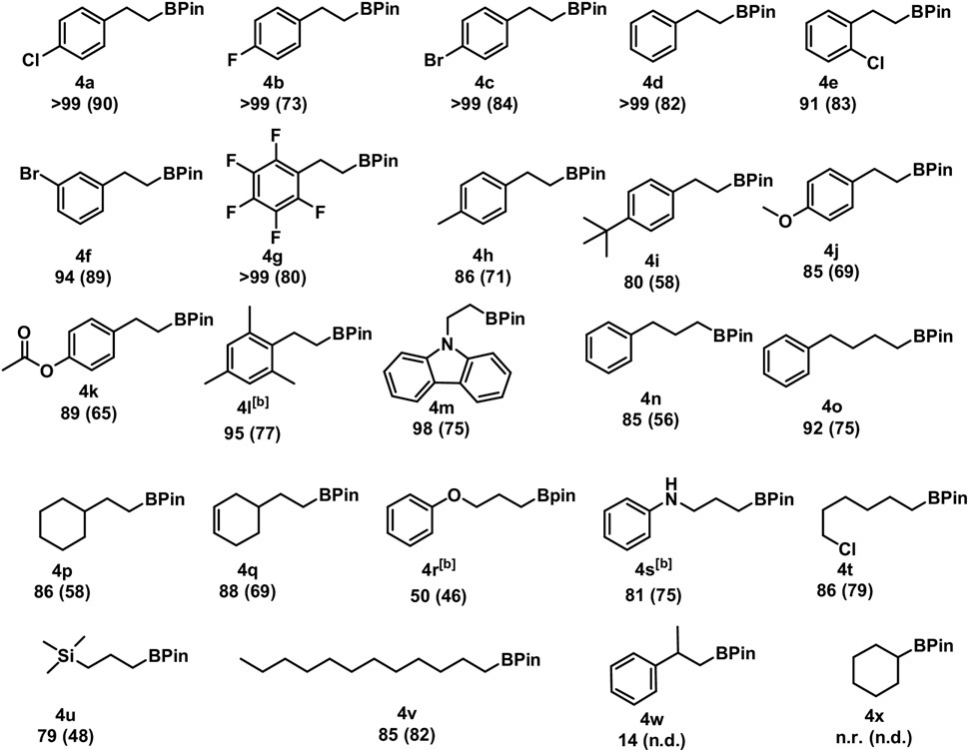
Substrate scope for the manganese‐catalyzed hydroboration of terminal alkenes.^[a]^ 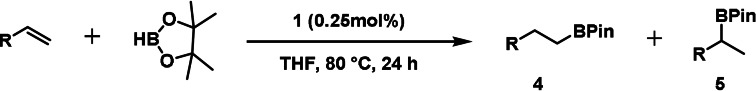

[a] Conditions: alkene (1.13 mmol, 1 equiv), HBPin (1.15 mmol, 1.02 equiv), **1** (0.25 mol %), 0.5 mL THF, 80 °C, 24 h, Ar, conversion, and isomer ratio (≥98:2 of **4**:**5**) determined by GC‐MS, isolated yield given in parenthesis. [b] **1** (1 mol %) was used.

To gain some mechanistic insights several experiments were carried out employing 4‐chlorostyrene as substrate. The homogeneity of the system was proven upon addition of one drop of Hg which did not lead to a loss of productivity. On the other hand, addition of 1 equiv of PMe_3_ resulted in only 8 % conversion which is in line with an uncatalyzed reaction (7 % conversion, Table 1, entry 10). This indicates that the reaction proceeds via an inner‐sphere reaction since PMe_3_ blocks the vacant coordination site of the actual catalyst. Interestingly, **1** reacts with pinacolborane to yield [Mn(dippe)(CO)_2_(κ^2^‐HBPin)] (**6**) in 68 % isolated yield. The structure of **6** was elucidated by X‐ray crystallography (Scheme 4). This complex could be detected by in situ NMR analysis upon reaction progress. Importantly, the same reactivity and selectivity for the hydroboration of 4‐chlorostyrene was achieved by employing complex **6** as catalyst.

We also studied the hydroboration of terminal alkynes. Apart from *E*‐, *Z‐* and geminal hydroborated products (**8**–**10**), significant amounts of the unsaturated *trans*‐1,2‐diborated isomer (**7**) was observed (Table 3), which is an important building block in organic chemistry.^[24h]^ This transformation is so far not known for any transition metal‐catalyst, since 1,2‐diboration of (terminal) alkynes typically afford *syn*‐1,2‐diborated compounds employing boron dimers such as B_2_Pin_2_.^[24]^ It should be mentioned that no formation of alkyne‐dimerization was observed under the given reaction conditions.

**Table 3:**
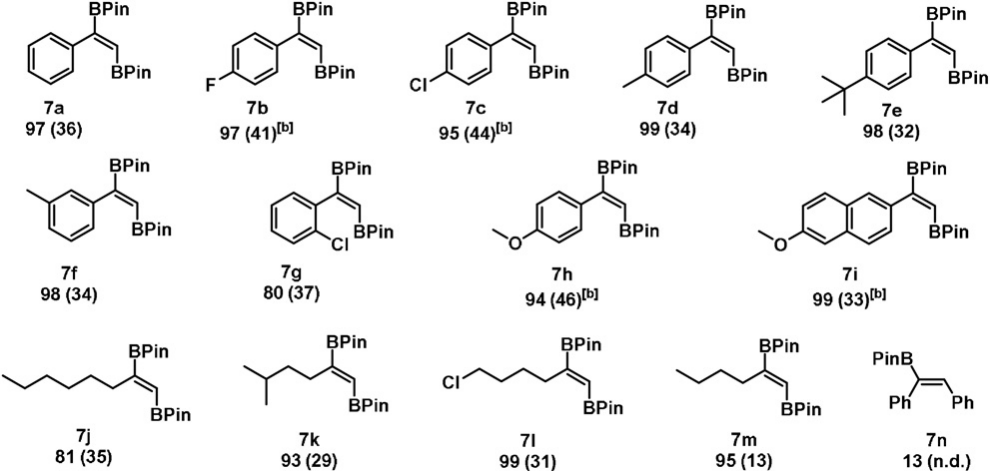
Substrate scope for the manganese‐catalyzed *trans*‐1,2 diboration of terminal alkynes.^[a]^ 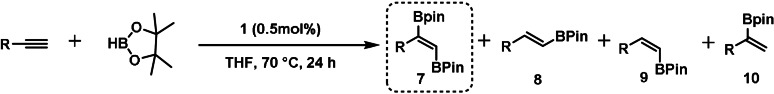

[a] Conditions: alkyne (1.13 mmol, 1 equiv), HBPin (1.7 mmol, 1.5 equiv), **1** (0.5 mol %), 0.5 mL THF, 70 °C, 24 h, Ar, conversion determined by GC‐MS, isolated yield given in parenthesis. [b] yield determined by GC‐MS using *n*‐dodecane as standard.

Upon optimization reactions (see SI), a selectivity of up to 55 % of the desired *trans*‐1,2‐diborated product was achieved with only 0.5 mol % of catalyst **1**. The formation of the *trans*‐1,2‐diborated product seems to be attributed to a massive formation of hydrogen gas and is thus described as an acceptorless process (vide infra). Investigation of the substrate scope revealed a broad applicability of the investigated transformation for aromatic (**7 a**–**7 i**) and aliphatic systems (**7 j**–**7 m**) (Table 3). The presence of the C−H bond of the terminal alkyne is crucial for this transformation, since in case of diphenylacetylene (**7 n**) only low conversion and no formation of the desired *trans*‐1,2‐diborated product was observed.

A variety of experiments were carried out in order to establish a plausible reaction mechanism (Scheme 3). Head space analysis showed that hydrogen gas is released during the reaction. This proves that the reaction operates via an acceptorless pathway. In absence of catalyst exclusively the unsaturated mono‐borated *E*‐isomer (**8 a**) is formed in low yield. Neither mono‐borylated compounds **8 a**, **9 a**, **10 a** nor the mono‐borylated alkyne species (**11**), which was detected in traces (<3 %) upon substrate scope investigations for some aromatic substrates, showed any reactivity under the given reaction conditions. The reaction seems to proceed via a concerted mechanism rather than a stepwise hydroboration/dehydrogenative borylation process. A deuterium labeling experiment employing phenylacetylene‐d_1_ revealed that only traces of deuterium are incorporated in the *trans*‐1,2‐diborated product. Interestingly, complex **6** exhibited a similar catalytic reactivity to **1** and may be considered as resting state which can be activated under these reaction conditions (Scheme 3).

**Scheme 3:**
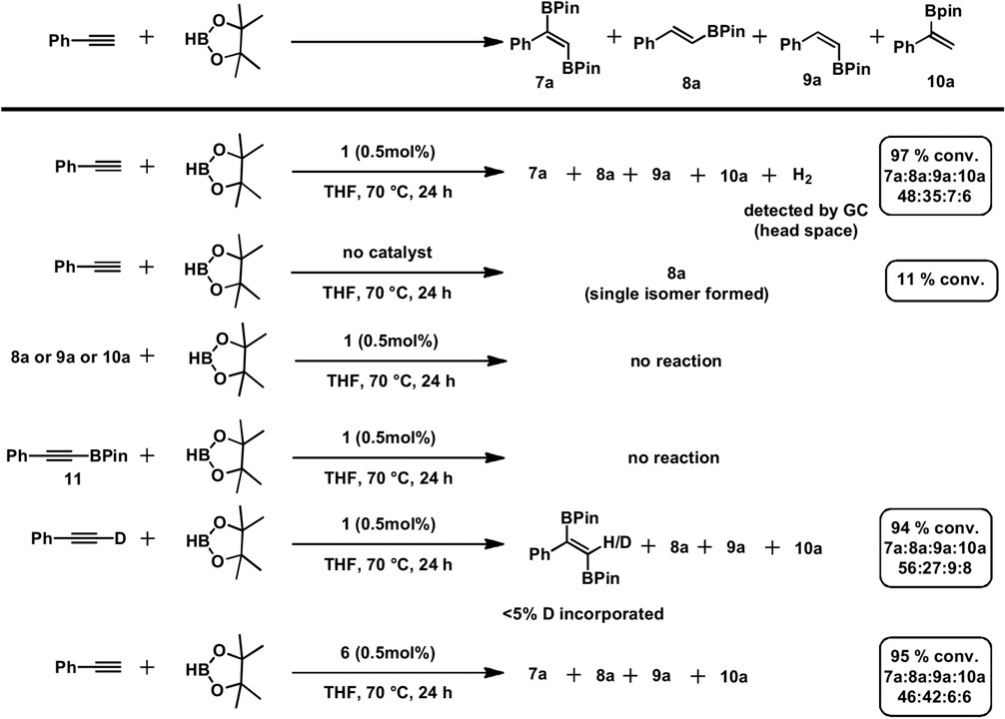
Mechanistic experiments for the manganese‐catalyzed *trans*‐1,2‐diboration of terminal alkynes.

The mechanism of the *trans*‐1,2‐diboration of terminal alkynes catalyzed by **1** was investigated in detail by DFT calculations using phenylacetylene as model substrate.^[25]^ The resulting free energy profiles are provided in the Supporting Information (Figures S1–S4). A simplified catalytic cycle is depicted in Scheme 4.

**Scheme 4:**
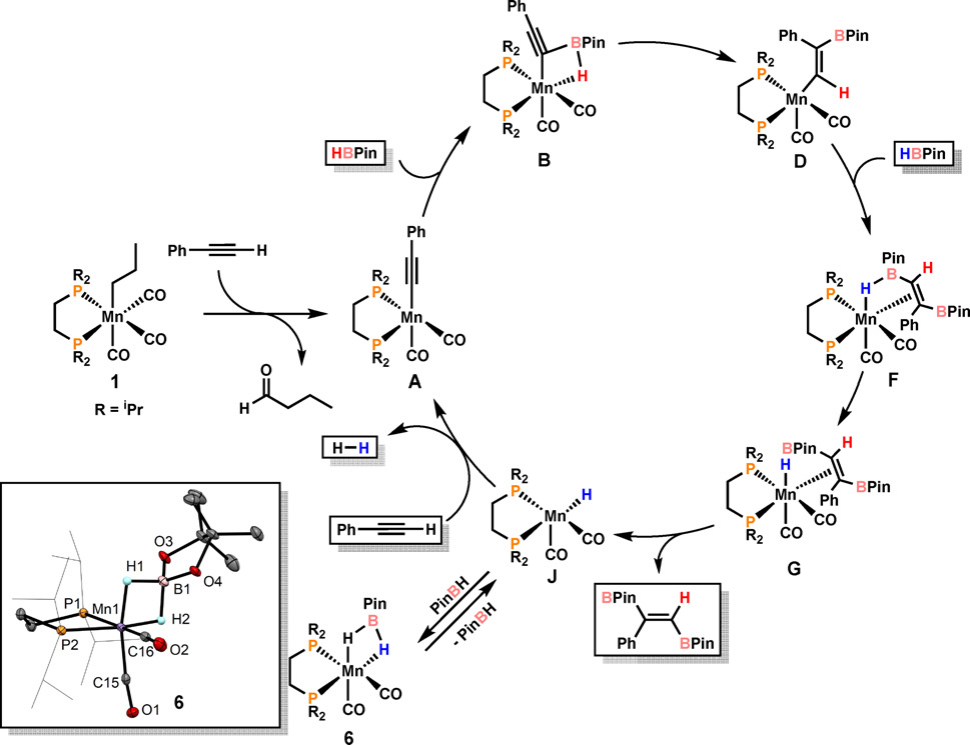
Simplified catalytic cycle for the *trans*‐1,2‐diboration of phenyl acetylene with HBPin. Inset: X‐ray structure of complex **6**.

Catalyst initiation, reported previously,^[21]^ starts from **1** to give the 16e^−^ acetylide catalyst [Mn(dippe)(CO)_2_(C≡*CPh*)] (**A**) together with liberated butanal. It has to be noted that the direct activation of the B−H bond of HBin to form the reactive 16e^−^ boryl intermediate [Mn(dippe)(CO)_2_(BPin)] was also considered but this process is less favorable by 8 kcal mol^−1^ in comparison to C−H bond activation of the terminal alkyne (see SI, Figure S4). This process however seems to take place in the case of alkene hydroboration.

Addition of HBPin to **A** results in the formation of intermediate **B** where B−C and Mn−H bonds are formed with the B−H bond remaining still intact. This is a facile process with a barrier of only 4 kcal mol^−1^ (**TS_AB_**). From **B**, the BPin moiety undergoes a 1,2‐boryl shift to form vinyl intermediate **D**. This is the rate‐determining step overcoming a barrier of 20 kcal mol^−1^ and is accompanied by B−H bond cleavage and formation of a new C−H bond. Addition of a second molecule of HBPin results in the formation of **F** where the second C−B bond is formed (the overall barrier for these steps is 11 kcal mol^−1^) (see SI Figure S1). Facile B−H bond cleavage in **F** results in the formation of the intermediate **G** containing a hydride ligand and the product coordinated in η^2^‐fashion. After product release, the hydride intermediate **J** is formed which reacts with phenyl acetylene to reform pre‐catalyst **A**. This transformation requires two steps involving C−H bond activation of the alkyne with concomitant formation of a dihydrogen intermediate which readily releases dihydrogen (for details see SI, Figures S2 and S3). **J** may also react with HBPin to give isolable **6** as a dormant species, which can be activated upon dissociation of HBPin.

In conclusion, the bench‐stable alkyl bisphosphine Mn^I^ complex *fac*‐[Mn(dippe)(CO)_3_(CH_2_CH_2_CH_3_)] turned out to be an efficient catalyst for an additive‐free hydroboration of terminal alkenes and the *trans*‐1,2‐diboration of terminal alkynes with HBPin. The diboration reaction is accompanied by dihydrogen release. In the case of alkenes *anti*‐Markovnikov hydroboration takes place, while in the case of alkynes the reaction proceeds with excellent *trans*‐1,2‐selectivity. The catalytic process is initiated by migratory insertion of a CO ligand into the Mn‐alkyl bond to yield an acyl intermediate which undergoes B−H bond cleavage of HBPin (in the case of alkenes) and rapid C−H bond cleavage (in the case of alkynes) forming the active 16 e^−^ Mn^I^ boryl and acetylide catalysts [Mn(dippe)(CO)_2_(BPin)] and [Mn(dippe)(CO)_2_(C≡CR)], respectively. A broad variety of aromatic and aliphatic alkenes and alkynes was efficiently and selectively borylated. Mechanistic insights are provided based on experimental data. In the case of the diboration reaction, a detailed mechanism is provided based on DFT calculations revealing that an acceptorless reaction pathway is operating involving dihydrogen release.

## Conflict of interest

The authors declare no conflict of interest.

## Supporting information

As a service to our authors and readers, this journal provides supporting information supplied by the authors. Such materials are peer reviewed and may be re‐organized for online delivery, but are not copy‐edited or typeset. Technical support issues arising from supporting information (other than missing files) should be addressed to the authors.

Supporting Information

Supporting Information
