# Supplementary material for: Hydroboration of Terminal Alkenes and trans‐1,2‐Diboration of Terminal Alkynes Catalyzed by a Manganese(I) Alkyl Complex
Source: Angew Chem Int Ed Engl. 2021 Oct 13;60(46):24488–92. doi: 10.1002/anie.202110736 (PMC8596825; doi:10.1002/anie.202110736)
Supplement: Supplementary file 2 — Supporting Information [file ANIE-60-24488-s001.pdf]

Angewandte Chemie
Eine Zeitschrift der Gesellschaft Deutscher Chemiker

Supporting Information

Hydroboration of Terminal Alkenes and *trans*-1,2-Diboration of Terminal Alkynes Catalyzed by a Manganese(I) Alkyl Complex

*Stefan Weber[+], Daniel Zobernig[+], Berthold Stöger, Luis F. Veiros, and Karl Kirchner**

anie_202110736_sm_miscellaneous_information.pdf
anie_202110736_sm_cif_xyz.zip

Supporting Information

---

1. General information..S1
2. Further optimization reactions...S2
3. Mechanistic experiments S4
4. X-ray structure determination...S10
5. Procedure for substrate scope and characterization of organic products.......................S10
6. Computational details S25
7. References..S29
8. NMR Spectra S32

1. General information

All used reagents and solvents were purchased from commercial suppliers and directly used without further purification, if not stated otherwise. Anhydrous MeOH, THF and toluene were dried over molecular sieve.

Preparative flash column chromatography was conducted manually using glass columns packed with silica gel 60 (Merck, 40-63 µm).

^{1}H-, ^{13}C{^{1}H}- and ^{31}P{^{1}H}-NMR were recorded in chloroform-*d*, methylene chloride-d_2 or benzene-d_6 solution on a Bruker Avance 250 (250 MHz) or Bruker Avance 400 (400 MHz). All chemical shifts (δ) are reported in ppm, using tetramethylsilane for ^{1}H and for ^{13}C{^{1}H}-, and H_3PO_4 for ^{31}P{^{1}H}-NMR spectra. All coupling constants (J) are reported in Hertz (Hz). The following abbreviations are used to describe multiplets: s = singlet, d = duplet, t = triplet, m = multiplet.

Complexes *fac*-[Mn(dippe)(CO)$_3$(Pr)][1] (dippe = 1,2-bis(di-*iso*-propylphosphino)ethane) (**1**), *fac*-[Mn(dippe)(CO)$_3$(Br)][2] (**2**) and *fac*-[Mn(dippe)(CO)$_3$(H)][3] (**3**) were synthesized according to literature. Phenylacetylene-d_1 (>98 % D) was synthesized from phenylacetylene, *n*-BuLi and D_2O. (*E*)-4,4,5,5-tetramethyl-2-styryl-1,3,2-dioxaborolane (**8a**) was synthesized form phenylacetylene and pinacolborane in a neat reaction for 3 days at 100 °C and evaporation of the volatiles. (*Z*)-4,4,5,5-tetramethyl-2-styryl-1,3,2-dioxaborolane (**9a**)[4] 4,4,5,5-tetramethyl-2-(1-phenylvinyl)-1,3,2-dioxaborolane (**10a**)[5] and 4,4,5,5-tetramethyl-2-(phenylethynyl)-1,3,2-dioxaborolane (**11**)[6] were synthesized according to literature.

GC–MS analyses were conducted on a ISQ LT Single quadrupole MS (Thermo Fisher) directly interfaced to a TRACE 1300 Gas Chromatographic systems (Thermo Fisher), using a Rxi-5Sil MS (30 m, 0.25mm ID) cross-bonded dimethyl polysiloxane capillary column at a carrier flow of He 1.5 mL/min. The oven program temperature was:

Method **A**: 100 °C (2 min)//35 °C/min//300 °C (4 min)

Method **B**: 40 °C (2.5 min)//12 °C/min//220 °C (2.5 min)

If not stated otherwise, Method **A** was used as default.

High-resolution accurate mass spectra were recorded on an Agilent 6545 QTOF equipped with an Agilent MMI ion source (Agilent Technologies, Santa Clara, CA, USA) which can be operated in mixed ESI and APCI mode. Measured accurate mass data for confirming calculated elemental compositions were within ±3 ppm accuracy.

2. Further optimization reactions

Further optimization reactions for the hydroboration of alkenes

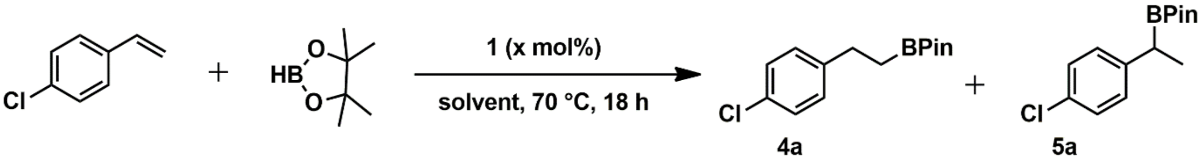

Inside an argon flushed glovebox, a screw cap vial (8 mL) was charged with 4-chlorostyrene (140 µL, 1.13 mmol, 1 equiv.), pinacolborane (167 - 246 µL, 1.15 - 1.69 mmol, 1.02 - 1.50 equiv.), **1** (0.5 - 5.0 mg, 0.1 - 1.0 mol%) and solvent (0.5 mL) and closed under argon atmosphere. The vial was transferred out of the glovebox and stirred for the indicated time at given temperature. The sample was allowed to reach room temperature, exposed to air and analyzed by GC-MS.

Table S1. Additional optimization reactions for the hydroboration of alkenes

Solvent	Reaction time	Temperature	Catalyst Loading	Pinacolborane (equiv.)	Conversion	4a:5a
neat	18 h	70°C	1 mol%	1.02.	>99 %	96:4
neat	18 h	70°C	0.5 mol%	1.02	88 %	97:3
toluene	18 h	70°C	0.5 mol%	1.02	82 %	98:2
THF	18 h	70°C	1 mol%	1.02	>99 %	97:3
THF	18 h	70°C	0.5 mol%	1.02	88 %	98:2
THF	18 h	70°C	1 mol%	1.02	>99 %	97:3
THF	24 h	70°C	0.5 mol%	1.02.	97 %	98:2
THF	24 h	70°C	0.5 mol%	1.20	88 %	98:2
THF	24 h	70°C	0.25 mol%	1.20.	70 %	99:1
THF	24 h	70°C	0.1 mol%	1.50.	33 %	>99:1
THF	24 h	80°C	0.5 mol%	1.20.	>99 %	97:3
THF	24 h	80°C	0.2 mol%	1.02	94 %	98:2

Optimization reactions for the trans-1,2-diboration of alkynes

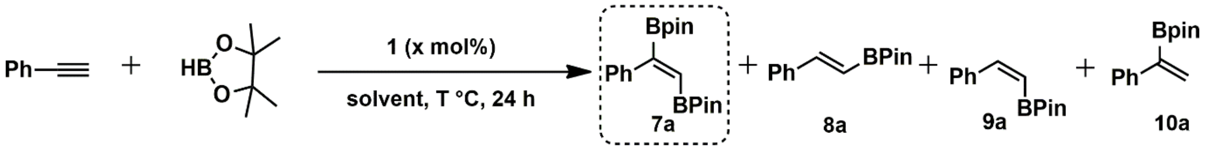

Inside an argon flushed glovebox, a screw cap vial (8 mL) was charged with phenylacetylene (124 µL, 1.13 mmol, 1 equiv.), pinacolborane (167 - 501 µL, 1.15 - 3.46 mmol, 1.02 - 3.06 equiv.), **1** (0.25 - 0.25 mol%) and solvent (0.5 mL) and closed under argon atmosphere. The vial was transferred out of the glovebox and stirred for 24 hours at the given temperature. The sample was allowed to reach room temperature, exposed to air and analyzed with GC-MS.

Table S2. Optimization reactions for the *trans*-1,2-diboration of alkynes

Solvent	Reaction time	Temperature	Catalyst Loading	Pincacolborane (equiv.)	Conversion	7a-8a-9a-10a
**THF**	24 h	70°C	0.25 mol%	1.50	88 %	54 - 32 - 7 - 7
**toluene**	24 h	70°C	0.25 mol%	1.50	65 %	61 - 28 - 7 - 3
**fluorobenzene**	24 h	70°C	0.25 mol%	1.50.	73 %	58 - 31 - 7 - 4
***n*-heptane**	24 h	70°C	0.25 mol%	1.50	61 %	60 - 29 - 9 - 2
**DME**	24 h	70°C	0.25 mol%	1.50	77 %	55 - 32 - 8 - 5
**DCE**	24 h	70°C	0.25 mol%	1.50.	92 %	54 - 30 -7 - 9
**-**	24 h	70°C	0.25 mol%	1.50	83 %	62 - 23 - 6 - 9
**THF**	24 h	60°C	0.50 mol%	1.50	56 %	55 - 29 - 7 - 9
**THF**	24 h	80°C	0.50 mol%	2.04.	89 %	54 - 33 - 9 - 4
**THF**	24 h	80°C	0.50 mol%	3.06.	97 %	39 - 52 - 7 - 2
**THF**	24 h	70°C	0.50 mol%	1.50.	97 %	48 - 39 - 7 - 6
**THF**	24 h	70°C	-	1.50	11 %	0 - 100 – 0 - 0

3. Mechanistic experiments

Synthesis of Complex **6**

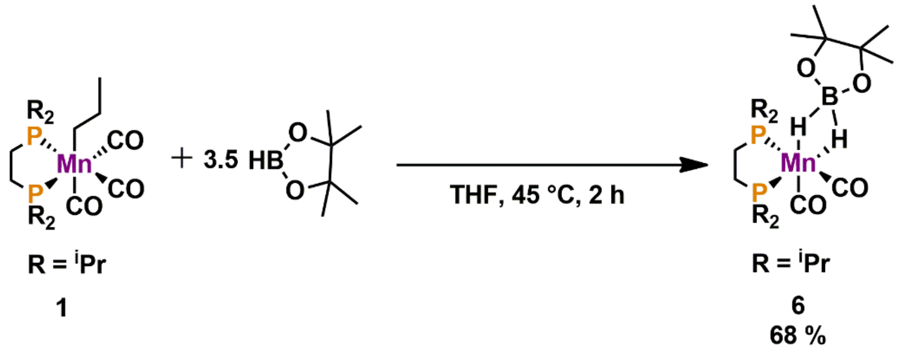

Inside an argon-flushed glovebox, an NMR-tube was charged with **1** (50 mg, 0.11 mmol, 1 equiv.), THF-d_8 (0.6 mL) and pinacolborane (50.5 mg, 0.39 mmol, 3.5 equiv.). The tube was sealed, transferred out of the glovebox and heated to 45 °C. The reaction was carefully monitored *via* ^{1}H- and ^{31}P{^{1}H}-NMR. Upon full conversion (approx. 2 h), the solution was transferred into a screw cap vial (8 mL) and the solvent was gently removed, giving a yellow, sticky residue, which was dried for 1 h. The residue was washed with *n*-pentane (3x1 mL), giving a slightly yellow solid. The solid was dried, yielding **6** as slightly yellow powder (38 mg, 68%). Single crystals suitable for single crystal analysis were obtained upon slow evaporation of a benzene solution.

^{1}H NMR (400 MHz, THF-d_8): δ = 2.56 (m, 1H), 2.41 – 2.28 (m, 1H), 2.23 (dq, J = 11.0, 7.0 Hz, 1H), 2.06 – 1.89 (m, 3H), 1.34 – 1.15 (m, 19H), 1.13 (d, J = 7.1 Hz, 4H), 1.10 (q, J = 3.2 Hz, 13H), 1.00 (dd, J = 15.2, 7.1 Hz, 3H), -10.5 – -11.2 (m, 1H), -11.8 – -12.7 (m, 1H).

^{13}C {^{1}H} NMR (63 MHz, THF-d_8): δ = 82.4, 29.5 (d, J = 22.7 Hz), 27.8 (dd, J = 21.8, 1.4 Hz), 26.7 (dd, J = 20.6, 2.4 Hz), 24.4 – 24.0 (m), 23.9, 23.6, 21.1, 20.8 (d, J = 3.9 Hz), 20.4 (d, J = 4.2 Hz), 18.7 (d, J = 2.1 Hz), 18.3 (dd, J = 5.5, 3.6 Hz), 18.1 – 17.8 (m), 17.0, 16.6 (d, J = 6.3 Hz). CO not observed

^{31}P{^{1}H} (163 MHz, THF-d_8): δ = 116.3 (br, 1P), 97.3 (br, 1P).

ATR-IR (solid, cm^{-1}): 1930 (ν_{CO}), 1856 (ν_{CO}).

HRMS (TOF ESI+): *m/z* calculated for $C_{22}H_{48}BMnO_4P_2Na$ $[M+Na]^+$: 526.2399, found 526.2308.

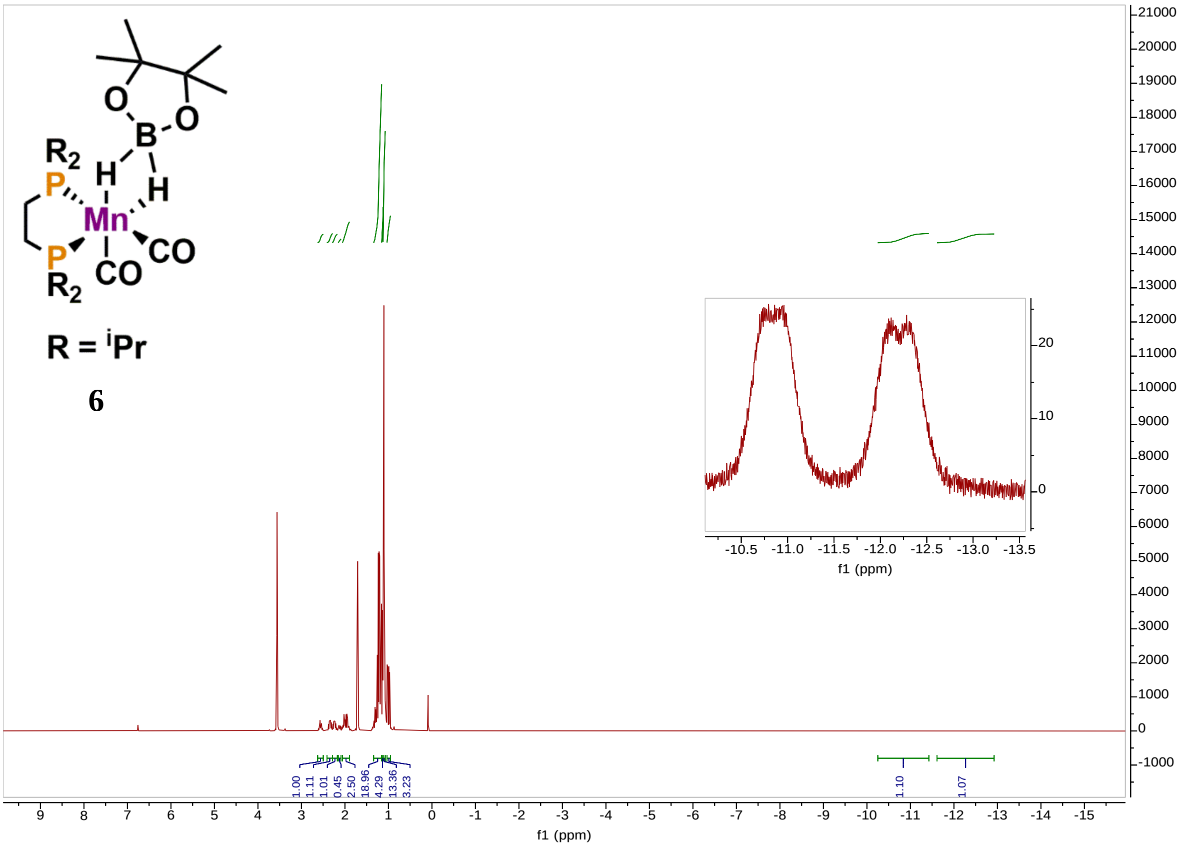

R = iPr
6
f1 (ppm)

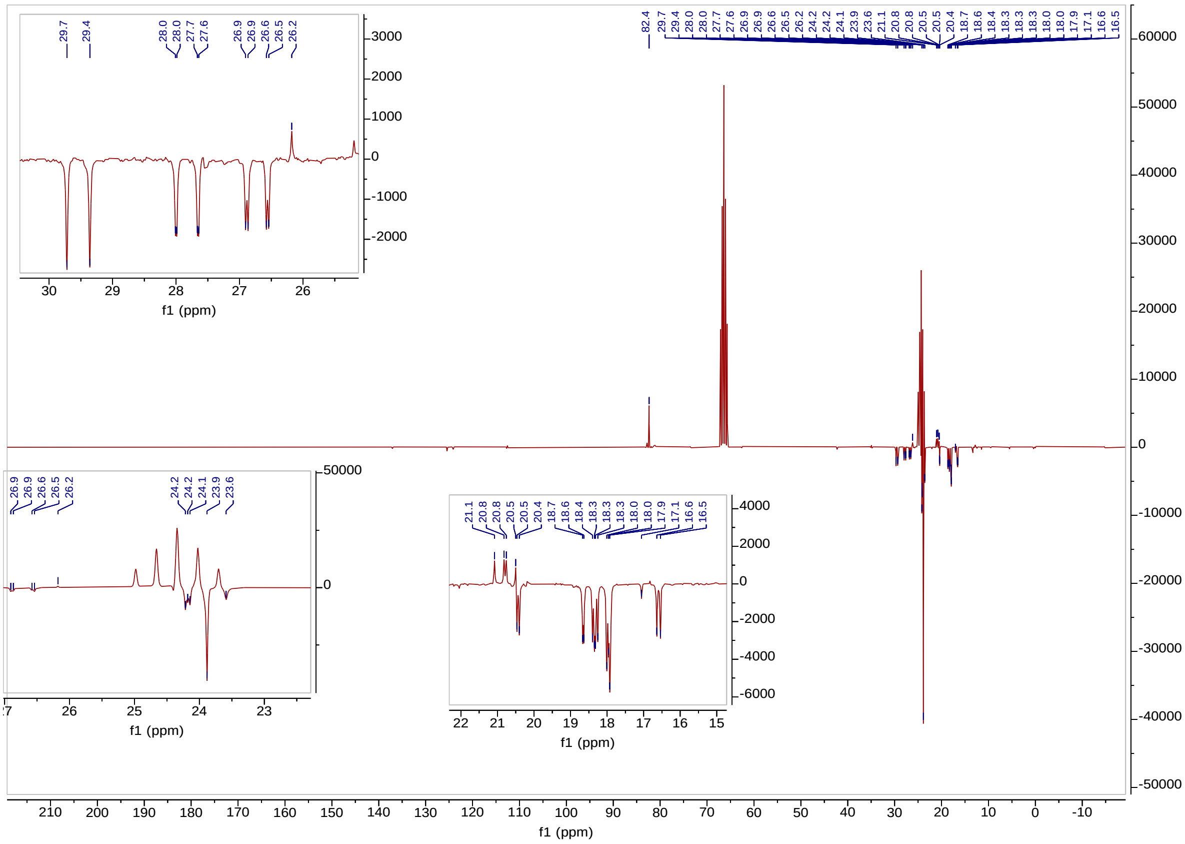

f1 (ppm)

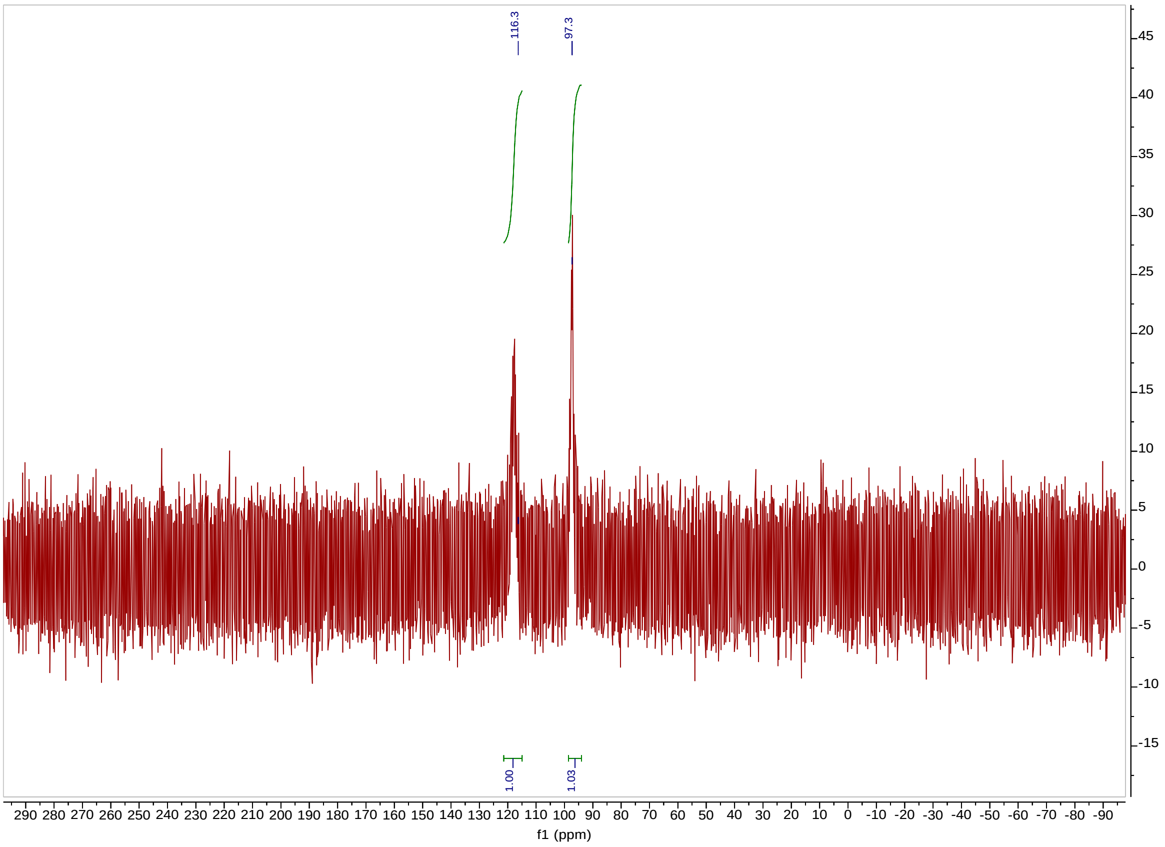

116.3
97.3
1.00
1.03
45
40
35
30
25
20
15
10
5
0
-5
-10
-15
290 280 270 260 250 240 230 220 210 200 190 180 170 160 150 140 130 120 110 100 90 80 70 60 50 40 30 20 10 0 -10 -20 -30 -40 -50 -60 -70 -80 -90
f1 (ppm)

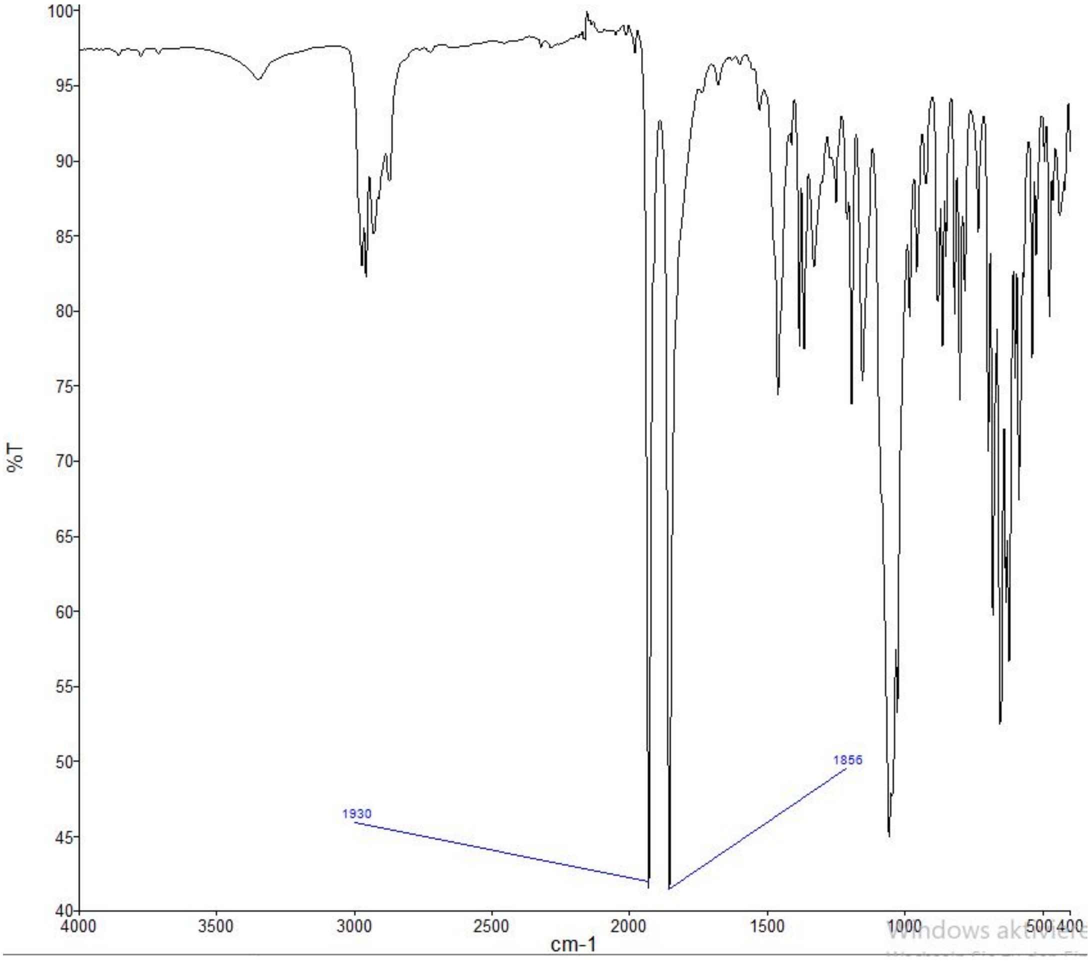

100
95
90
85
80
75
70
65
60
55
50
45
40
%T
1930
1856
4000
3500
3000
2500
2000
1500
1000
500 400
cm-1

Synthetic procedure for mercury drop test

Inside an argon flushed glovebox, a screw cap vial (8 mL) was charged with substrate (4-Cholorstyrene as alkene or phenylacetylene as alkyne) (1.13 mmol, 1 equiv.), pinacolborane (1.02 equiv. for alkene or 1.5 equiv. or alkyne), **1** (0.25 mol% for alkene or 0.5 mol% for alkyne), 1 drop of mercury, THF (0.5 mL) and closed under argon atmosphere. The vial was transferred out of the glovebox and stirred for 24 hours at 70 °C (for alkyne) or 80 °C (for alkene). The sample was allowed to reach room temperature, exposed to air analyzed with GC-MS.

No significant decrease in reactivity or selectivity could be observed for alkenes or alkynes.

Synthetic procedure for poisoning with trimethylphosphine test

Inside an argon flushed glovebox, a screw cap vial (8 mL) was charged with substrate (4-Cholorstyrene as alkene or phenylacetylene as alkyne) (1.13 mmol, 1 equiv.), pinacolborane (1.02 equiv. for alkene or 1.5 equiv. or alkyne), **1** (0.25 mol% for alkene or 0.5 mol% for alkyne), PMe_3 (1.13 mmol, 1 equiv.), THF (0.5 mL) and closed under argon atmosphere. The vial was transferred out of the glovebox and stirred for 24 hours at 70 °C (for alkyne) or 80 °C (for alkene). The sample was allowed to reach room temperature, exposed to air analyzed with GC-MS.

Only traces of product could be observed for alkenes or alkynes.

Synthetic procedure for detection of hydrogen gas for *trans*-1,2-boration of phenylacetylene

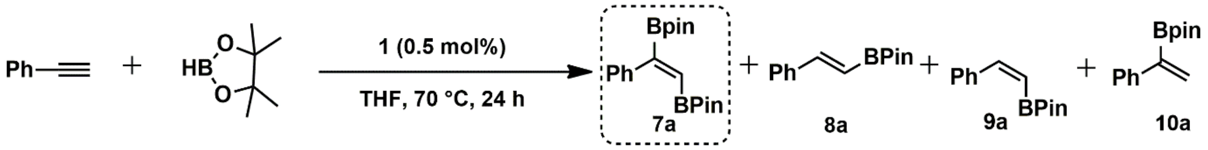

Inside an argon flushed glovebox, a screw cap vial with septum (8 mL) was charged with phenylacetylene (1.13 mmol, 1 equiv.), pinacolborane (246 µL, 1.50 mmol, 1.5 equiv.), catalyst (2.5 mg, 0.5 mol%) and THF (0.5 mL) and closed under argon atmosphere. The vial was transferred out of the glovebox and stirred for 24 hours at 70 °C. The septum was penetrated with a Hamilton syringe and 50 µL of the headspace was taken up in syringe. The sample was diluted to 200 µL with argon inside the syringe and injected in the GC.

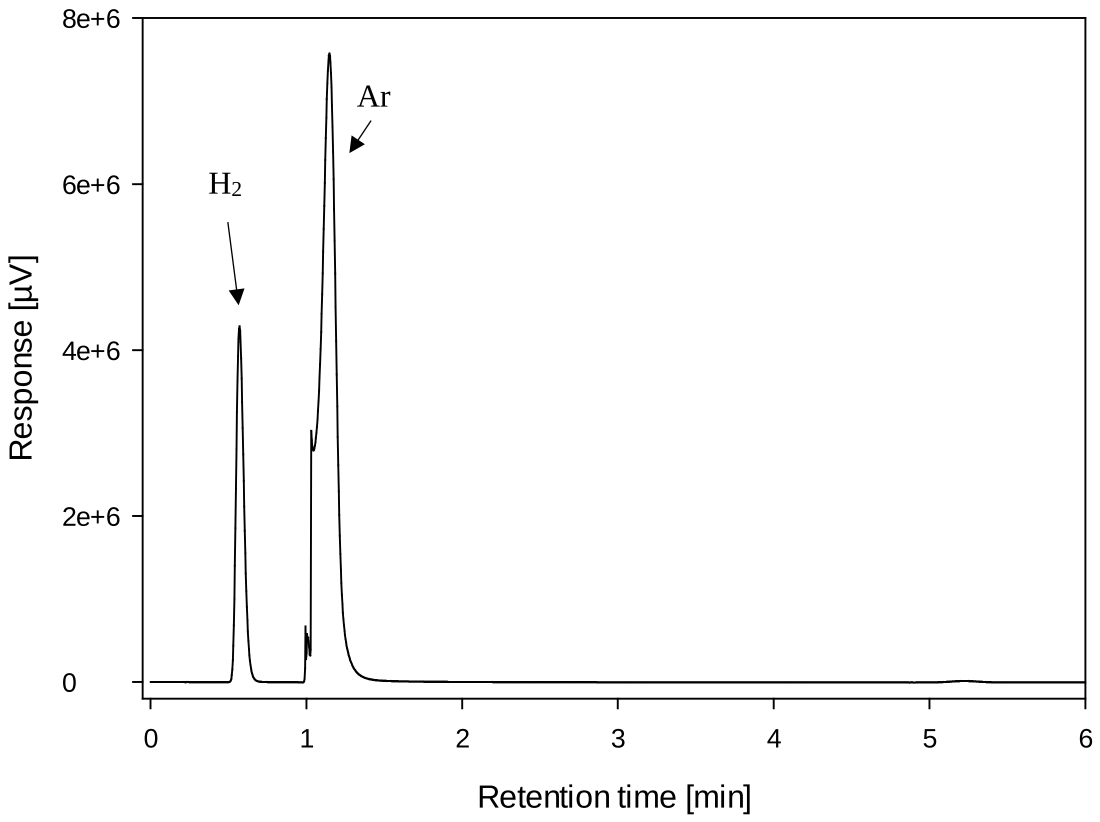

<u>Synthetic procedure for deuterium label experiment with phenylacetylene-*d₁*</u>

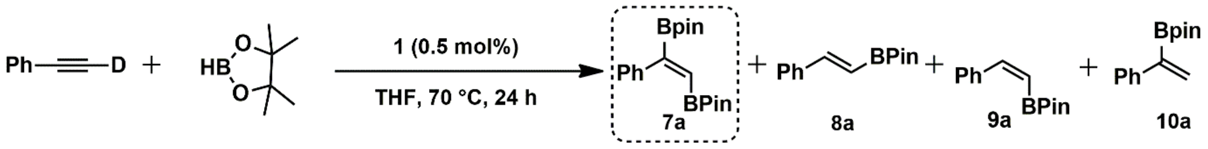

Inside an argon flushed glovebox, a screw cap vial (8 mL) was charged with phenylacetylene-d_1 (1.13 mmol, 1 equiv.), pinacolborane (246 µL, 1.50 mmol, 1.5 equiv.), catalyst (2.5 mg, 0.5 mol%) and THF (0.5 mL) and closed under argon atmosphere. The vial was transferred out of the glovebox and stirred for 24 hours at 70 °C. The sample was allowed to reach room temperature, exposed to air and analyzed with GC-MS. The solvent was removed, and the residue purified by column chromatography (PE/Et_2O 8:1). A deuterium content of <5% could be detected in the isolated product.

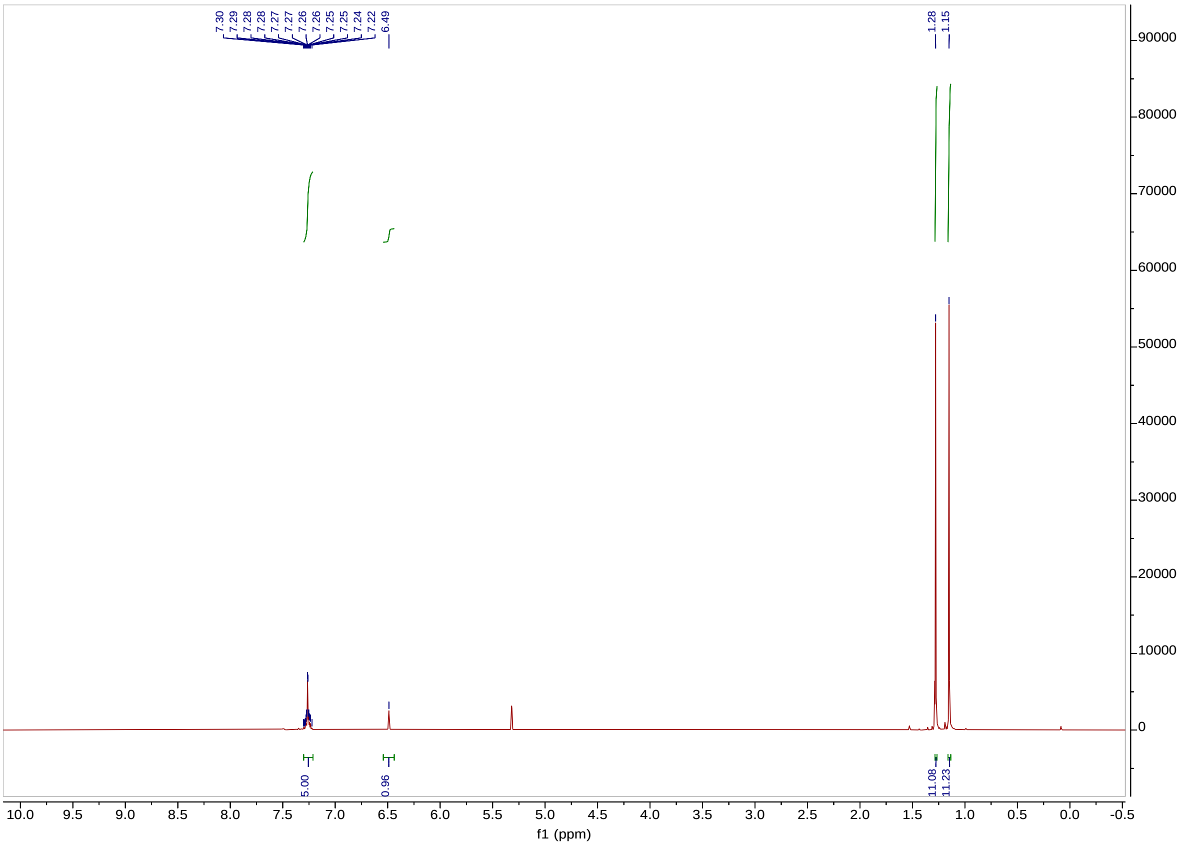
7.30
7.29
7.28
7.28
7.27
7.27
7.26
7.26
7.25
7.25
7.24
7.22
6.49
1.28
1.15
90000
80000
70000
60000
50000
40000
30000
20000
10000
0
5.00
0.96
11.08
11.23
10.0 9.5 9.0 8.5 8.0 7.5 7.0 6.5 6.0 5.5 5.0 4.5 4.0 3.5 3.0 2.5 2.0 1.5 1.0 0.5 0.0 -0.5
f1 (ppm)

4. X-ray structure determination

X-ray diffraction data of **6** (CCDC 2102243) were collected at T = 100 K in a dry stream of nitrogen on a Bruker Kappa APEX II diffractometer system using graphite-monochromatized Mo-$K\alpha$ radiation (λ = 0.71073 Å) and fine sliced φ- and ω-scans. Data were reduced to intensity values with SAINT and an absorption correction was applied with the multi-scan approach implemented in SADABS.[7] The structure was solved by the dual-space approach implemented in SHELXT[8]and refined against F^2 with SHELXL.[9] Non-hydrogen atoms were refined with anisotropic displacement parameters. H atoms attached to C were placed in calculated positions and thereafter refined as riding on the parent atoms. Borane H atoms were located from difference Fourier maps and refined freely. Molecular graphics were generated with the program MERCURY.[10]

5. Procedure for substrate scope and characterization of organic products

Substrate scope of terminal alkenes

Inside an argon flushed glovebox, a screw cap vial (8 mL) was charged with substrate (1.13 mmol, 1 equiv.), pinacolborane (167 µL, 1.15 mmol, 1.02 equiv.), catalyst (1.25 - 5.0 mg, 0.25 - 1.0 mol%) and THF (0.5 mL) and closed under argon atmosphere. The vial was transferred out of the glovebox and stirred for 24 hours at 80 °C. The sample was allowed to reach room temperature, exposed to air analyzed with GC-MS. The solution was filtered through a thin pad of *silica* with a solvent (*vide infra*). The solution was stirred for approx. 2 minutes with water (0.5 mL), extracted with solvent (5 mL) and dried in *vacuo*.

4,4,5,5-Tetramethyl-2-[2-(4-Chlorophenyl)ethyl] -1,3,2-dioxaborolane (**4a**)

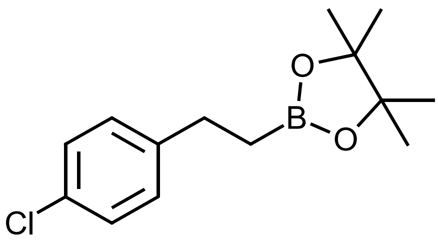

4-Chlorostyrene (143 µL, 1.13 mmol, 1 eq.); catalyst (1.25 mg, 2.8 µmol, 0.25 mol%); eluent: *n*-pentane; extracted with *n*-pentane; 271 mg (90 %) of a colorless oil.

^{1}H NMR (250 MHz, $CDCl_3$): δ 7.25 – 7.19 (m, 2H), 7.17 – 7.10 (m, 2H), 2.71 (t, J = 7.9 Hz, 2H), 1.21 (s, 12H), 1.15 – 1.07 (m, 2H) ppm.
^{13}C {^{1}H} NMR (63 MHz, $CDCl_3$): δ = 143.0, 131.3, 129.5, 128.4, 83.3, 29.5, 24.9, 13.0 ppm.
RT (GC): 6.22 min MS: 266.06 m/z $[M]^+$
These spectroscopic data correspond to reported data.[11]

2-[2-(4-Fluorophenyl)ethyl]-4,4,5,5-tetramethyl-1,3,2-dioxaborolane (**4b**)

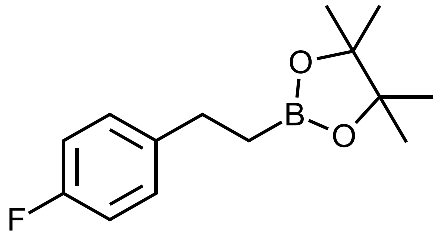

4-Fluorostyrene (134 µL, 1.13 mmol, 1 eq.); catalyst (1.25 mg, 2.8 µmol, 0.25 mol%); eluent: dichloromethane; extracted with dichloromethane; 246 mg (87 %) of a colorless oil.

^{1}H NMR (250 MHz, $CDCl_3$): δ = 7.16 (t, *J* = 6.7 Hz, 2H), 6.93 (t, *J* = 8.4 Hz, 2H), 2.71 (t, *J* = 8.0 Hz, 2H), 1.21 (s, 12H), 1.11 (t, *J* = 7.6 Hz, 2H) ppm.

^{13}C {^{1}H} NMR (63 MHz, $CDCl_3$): δ = 161.2 (d, *J* = 242.8 Hz), 140.0 (d, *J* = 3.1 Hz), 129.4 (d, *J* = 7.7 Hz), 114.9 (d, *J* = 21.0 Hz), 83.2, 29.2, 24.8, 13.4 ppm.

RT (GC): 5.54 min MS: 250.20 m/z $[M]^+$

These spectroscopic data correspond to reported data.[11]

2-[2-(4-Bromphenyl)ethyl]-4,4,5,5-tetramethyl-1,3,2-dioxaborolane (**4c**)

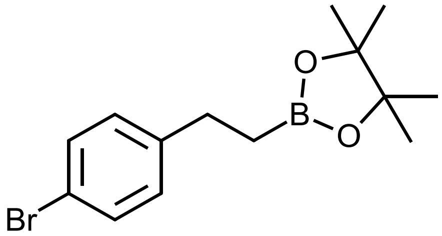

4-Bromostyrene (147 µL, 1.13 mmol, 1 eq.); catalyst (1.25 mg, 2.8 µmol, 0.25 mol%); eluent: dichloromethane; extracted with dichloromethane; 288 mg (82 %) of a colorless oil.

^{1}H NMR (250 MHz, $CDCl_3$): δ = 7.37 (d, *J* = 8.2 Hz, 2H), 7.08 (d, *J* = 8.2 Hz, 2H), 2.69 (t, *J* = 8.1 Hz, 2H), 1.21 (s, 12H), 1.10 (t, *J* = 8.2 Hz, 2H) ppm.

^{13}C {^{1}H} NMR (63 MHz, $CDCl_3$): δ = 143.3, 131.2, 129.8, 119.2, 83.1, 29.4, 24.8, 12.9 ppm.

RT (GC): 6.56 min MS: 310.14, 312.14 m/z $[M]^+$

These spectroscopic data correspond to reported data.[11]

4,4,5,5-Tetramethyl-2-(2-phenylethyl)-1,3,2-dioxaborolane (**4d**)

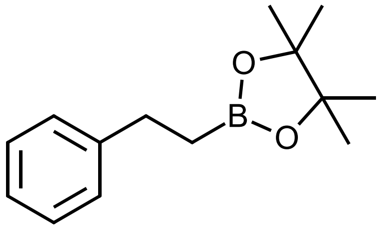

Styrene (129 µL, 1.13 mmol, 1 eq.); catalyst (1.25 mg, 0.25 mol%); eluent: *n*-pentane and diethyl ether (10:1); extracted with *n*-pentane; 190 mg (73 %) of a colorless oil.

^{1}H NMR (250 MHz, $CDCl_3$): δ = 7.47 – 7.13 (m, 5H), 2.78 (t, *J* = 8.1 Hz, 2H), 1.25 (s, 12H), 1.17 (t, *J* = 7.7 Hz, 2H) ppm.

^{13}C {^{1}H} NMR (63 MHz, $CDCl_3$): δ = 144.4, 128.2, 128.1, 125.6, 83.1, 30.0, 24.9, 13.0 ppm.

RT (GC): 5.35 min MS: 232.22 m/z $[M]^+$

These spectroscopic data correspond to reported data.[11]

2-[2-(2-Chlorophenyl)ethyl]-4,4,5,5-tetramethyl-1,3,2-dioxaborolane (**4e**)

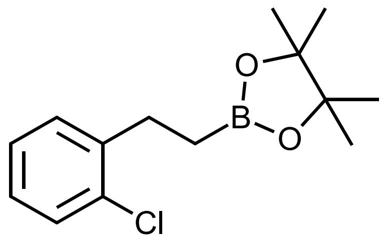

2-Chlorostyrene (144 µL, 1.13 mmol, 1 eq.); catalyst (1.25 mg, 2.8 µmol, 0.25 mol%); eluent: dichloromethane; extracted with dichloromethane; 248 mg (83 %) of a colorless oil.

^{1}H NMR (250 MHz, $CDCl_3$): δ = 7.34 – 7.26 (m, 2H), 7.21 – 7.07 (m, 2H), 2.87 (t, *J* = 8.3 Hz, 2H), 1.27 (s, 12H), 1.18 (t, *J* = 8.0 Hz, 2H) ppm.

^{13}C {^{1}H} NMR (63 MHz, $CDCl_3$): δ = 145.1, 133.9, 129.9, 129.4, 127.1, 126.7, 83.3, 27.9, 24.9, 11.8 ppm.

RT (GC): 6.18 min MS: 266.05 m/z $[M]^+$

These spectroscopic data correspond to reported data.[12]

2-[2-(3-Bromophenyl)ethyl]-4,4,5,5-tetramethyl-1,3,2-dioxaborolane (**4f**)

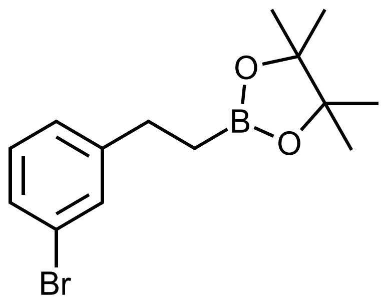

3-Bromostyrene (147 µL, 1.13 mmol, 1 eq); catalyst (1.25 mg, 2.8 µmol, 0.25 mol%); eluent: dichloromethane; extracted with dichloromethane; 310 mg (89 %) of a colorless oil.

^{1}H NMR (400 MHz, CD_2Cl_2): δ = 7.37 (s, 1H), 7.30 – 7.26 (m, 1H), 7.16 – 7.11 (m, 2H), 2.68 (t, *J* = 8.0 Hz, 2H), 1.19 (s, 12H), 1.06 (t, *J* = 8.0 Hz, 2H) ppm.

^{13}C {^{1}H} NMR (101 MHz, CD_2Cl_2): δ = 147.5, 131.5, 130.2, 128.9, 127.2, 122.5, 83.5, 30.1, 25.0, 13.8 ppm.

RT (GC): 6.48 min MS: 309.99, 311.99 m/z $[M]^+$

These spectroscopic data correspond to reported data.[13]

2-[2-(2,3,4,5,6-Pentafluorophenyl)ethyl]-4,4,5,5-tetramethyl-1,3,2-dioxaborolane (**4g**)

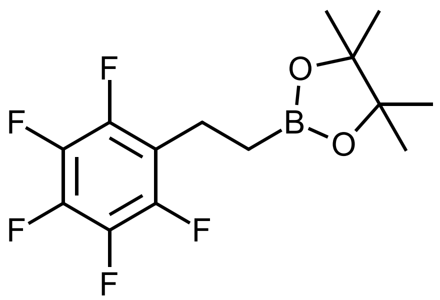

2,3,4,5,6-Pentafluorostyrene (155 µL, 1.13 mmol, 1 eq); catalyst (1.25 mg, 2.8 µmol, 0.25 mol%); eluent: dichloromethane; extracted with dichloromethane; 291 mg (80 %) of a colorless oil.

^{1}H NMR (400 MHz, $CDCl_3$): δ = 2.78 (t, *J* = 8.2 Hz, 2H), 1.23 (s, 12H), 1.10 (t, *J* = 7.9 Hz, 2H) ppm.

^{13}C {^{1}H} NMR (101 MHz, $CDCl_3$): δ = 144.9 (m), 139.3 (m), 137.3 (m), 117.2 (m), 83.5, 24.8, 17.0, 11.3 ppm.

RT (GC): 5.11 min MS: 322.19 m/z $[M]^+$

These spectroscopic data correspond to reported data.[13]

<u>4,4,5,5-Tetramethyl-2-[2-(p-tolyl)ethyl]-1,3,2-dioxaborolane</u> (**4h**)

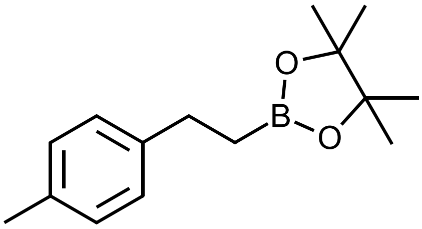

4-Methylstyrene (148 µL, 1.13 mmol, 1 eq.); catalyst (1.25 mg, 0.25 mol%); eluent: *n*-pentane; extracted with *n*-pentane; 197 mg (71 %) of a colorless oil.

^{1}H NMR (250 MHz, $CDCl_3$): δ = 7.22 – 7.03 (m, 4H), 2.72 (t, *J* = 8.0 Hz, 2H), 2.31 (s, 3H), 1.24 (s, 12H), 1.13 (t, *J* = 8.7 Hz, 2H) ppm.

^{13}C {^{1}H} NMR (63 MHz, $CDCl_3$): δ = 141.4, 134.9, 128.9, 127.9, 83.1, 29.5, 24.8, 21.0, 13.0 ppm.

RT (GC): 5.70 min MS: 246.21 m/z $[M]^+$

These spectroscopic data correspond to reported data.[11]

<u>2-[2-(4-tert-Butylphenyl)ethyl]-4,4,5,5-tetramethyl-1,3,2-dioxaborolane</u> (**4i**)

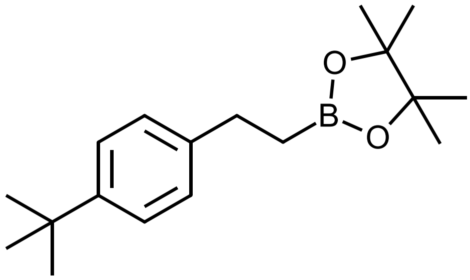

4-tert-Butylstyrene (206 µL, 1.13 mmol, 1 eq.); catalyst (1.25 mg, 2.8 µmol, 0.25 mol%); eluent: *n*-pentane; extracted with *n*-pentane; 188 mg (59 %) of a colorless oil.

^{1}H NMR (250 MHz, $CDCl_3$): δ = 7.37 - 7.27 (m, 2H), 7.23 – 7.12 (m, 2H), 2.72 (t, J = 8.3 Hz, 2H), 1.30 (s, 9H), 1.22 (s, 12H), 1.14 (t, J = 7.9 Hz, 2H) ppm.

^{13}C {^{1}H} NMR (63 MHz, $CDCl_3$): δ = 148.3, 141.4, 127.8, 125.1, 83.1, 31.6, 31.4, 29.5, 24.9, 13.2 ppm.

RT (GC): 17.00 min; MS: 288.26 m/z $[M]^+$ (Method **B**)

These spectroscopic data correspond to reported data.[13]

<u>2-[2-(4-Methoxyphenyl)ethyl]-4,4,5,5-tetramethyl-1,3,2-dioxaborolane</u> (**4j**)

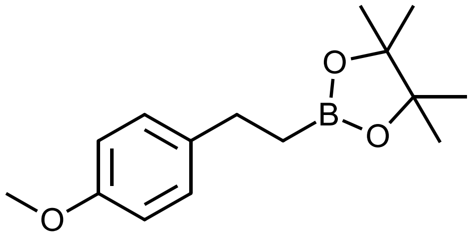

4-Methoxystyrene (150 µL, 1.13 mmol, 1 eq); catalyst (1.25 mg, 2.8 µmol, 0.25 mol%); eluent: *n*-pentane and diethyl ether (40:1); extracted with *n*-pentane; 204 mg (69 %) of a slightly yellow oil.

^{1}H NMR (250 MHz, $CDCl_3$): δ = 7.14 (d, *J* = 8.5 Hz, 2H), 6.81 (d, *J* = 8.6 Hz, 2H), 3.78 (s, 3H), 2.69 (t, *J* = 8.0 Hz, 2H), 1.22 (s, 12H), 1.11 (t, *J* = 8.2 Hz, 2H) ppm.

^{13}C {^{1}H} NMR (63 MHz, $CDCl_3$): δ = 157.6, 136.6, 128.9, 113.6, 83.1, 55.2, 29.1, 24.8, 13.2 ppm.

RT (GC): 6.22 min MS: 262.21 m/z $[M]^+$

These spectroscopic data correspond to reported data.[11]

4-[2-(4,4,5,5-Tetramethyl-1,3,2-dioxaborolan-2-yl)ethyl]phenyl acetate (**4k**)

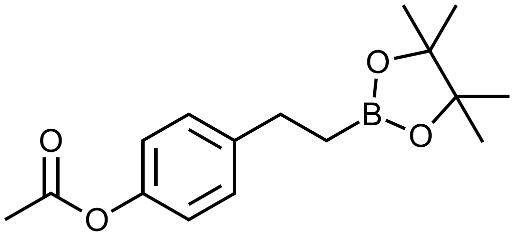

4-Vinylphenyl acetate (172 µL, 1.13 mmol, 1 eq); catalyst (1.25 mg, 2.8 µmol, 0.25 mol%); eluent: dichloromethane; extracted with dichloromethane; 213 mg (65 %) of a colorless oil.

^{1}H NMR (400 MHz, CD_2Cl_2): δ = 7.22 (d, *J* = 8.7 Hz, 2H), 6.96 (d, *J* = 8.6 Hz, 2H), 2.72 (t, *J* = 8.1 Hz, 2H), 2.25 (s, 3H), 1.21 (s, 12H), 1.09 (t, *J* = 8.2 Hz, 2H) ppm.

^{13}C {^{1}H} NMR (101 MHz, CD_2Cl_2): δ = 170.4, 149.5, 143.0, 129.7, 122.1, 83.9, 30.2, 25.4, 21.7, 13.9 ppm.

RT (GC): 17.64 min MS: 290.25 m/z $[M]^+$ (Method **B**)

These spectroscopic data correspond to reported data.[11]

4,4,5,5-Tetramethyl-2-[2-(2,4,6-Trimethylphenyl)ethyl]- 1,3,2-dioxaborolane (**4l**)

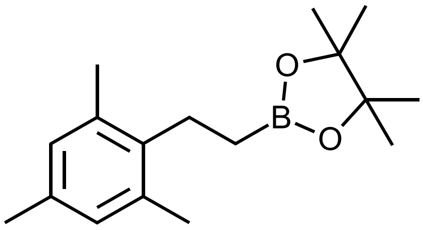

2,4,6-Trimethylstyrol (180 µL, 1.13 mmol, 1 eq.); catalyst (5 mg, 11.3 µmol 1 mol%), eluent: *n*-pentane, extracted with *n*-pentane; 236 mg (77 %) of a colorless oil.

^{1}H NMR (400 MHz, $CDCl_3$): δ = 6.83 (s, 2H), 2.69 (t, *J* = 8.7 Hz, 2H), 2.32 (s, 6H), 2.26 (s, 3H), 1.29 (s, 12H), 0.98 (t, *J* = 8.8 Hz, 2H) ppm.

^{13}C {^{1}H} NMR (101 MHz, $CDCl_3$): δ = 138.6, 135.7, 134.7, 128.9, 83.2, 25.0, 23.4, 20.9, 19.8, 11.4 ppm.

RT (GC): 6.52 min MS: 274.13 m/z $[M]^+$

These spectroscopic data correspond to reported data.[14]

9-(2-(4,4,5,5-tetramethyl-1,3,2-dioxaborolan-2-yl)ethyl)9H-carbazole (**4m**)

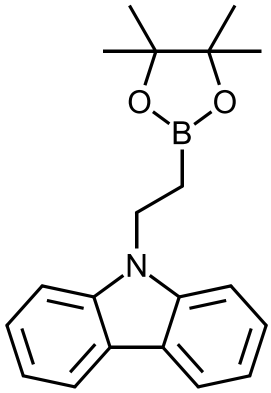

N-Vinylcarbazole (218 mg, 1.13 mmol, 1 eq.); catalyst (1.25 mg, 2.8 µmol, 0.25 mol%); eluent: dichloromethane; extracted with dichloromethane; 272 mg (75 %) of a slightly yellow solid.

^{1}H NMR (250 MHz, $CDCl_3$): δ = 8.08 (d, *J* = 7.5 Hz, 2H), 7.56 – 7.38 (m, 4H), 7.29 – 7.16 (m, 2H), 4.47 (t, *J* = 8.0 Hz, 2H), 1.42 (t, *J* = 7.4 Hz, 2H), 1.21 (s, 12H) ppm.
^{13}C {^{1}H} NMR (63 MHz, $CDCl_3$): δ = 140.0, 125.5, 123.0, 120.3, 118.7, 109.1, 83.6, 38.8, 24.9, 11.8 ppm.
RT (GC): 8.45 min MS: 321.26 m/z $[M]^+$
These spectroscopic data correspond to reported data.[11]

4,4,5,5-Tetramethyl-2-(3-phenylpropyl)-1,3,2-dioxaborolane (**4n**)

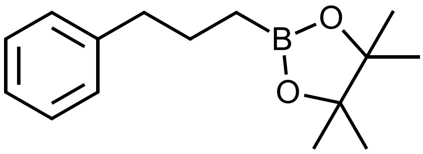

Allylbenzene (149 µL, 1.13 mmol, 1 eq.); catalyst (1.25 mg, 2.8 µmol, 0.25 mol%); eluent: *n*-pentane and diethyl ether (10:1); extracted with *n*-pentane; 155 mg (56 %) of a colorless oil.

^{1}H NMR (250 MHz, $CDCl_3$): δ = 7.46 – 7.12 (m, 5H), 2.58 (t, *J* = 7.6 Hz, 2H), 1.70 (t, J = 7.7 Hz, 2H), 1.21 (s, 12H), 0.79 (t, *J* = 7.9 Hz, 2H) ppm.
^{13}C {^{1}H} NMR (63 MHz, $CDCl_3$): δ = 142.7, 128.6, 128.2, 125.6, 82.9, 38.6, 26.2, 24.9, 11.2 ppm.
RT (GC): 15.34 min MS: 246.25 m/z $[M]^+$ (Method **B**)
These spectroscopic data correspond to reported data.[11]

4,4,5,5-Tetramethyl-2-(4-Phenylbutyl) -1,3,2-dioxaborolane (**4o**)

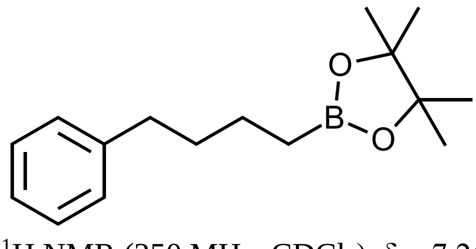

4-Phenylbutene (169 µL, 1.13 mmol, 1 eq.); catalyst (1.25 mg, 2.8 µmol, 0.25 mol%); eluent: *n*-pentane; extracted with *n*-pentane; 218 mg (75 %) of a colorless oil.

^{1}H NMR (250 MHz, $CDCl_3$): δ = 7.25 – 7.09 (m, 5H), 2.57 (t, *J* = 7.7 Hz, 2H), 1.71 – 1.52 (m, 2H), 1.52 – 1.39 (m, 2H), 1.21 (s, 12H), 0.78 (t, *J* = 7.9 Hz, 2H) ppm.
^{13}C {^{1}H} NMR (101 MHz, $CDCl_3$): δ = 143.1, 128.5, 128.3, 125.6, 83.0, 35.9, 34.3, 25.0, 23.9, 11.4 ppm.

RT (GC): 6.28 min MS: 260.13 m/z $[M]^+$

These spectroscopic data correspond to reported data.[12]

<u>2-[2-(cyclohexyl)ethyl]-4,4,5,5-tetramethyl-1,3,2-dioxaborolane</u> (**4p**)

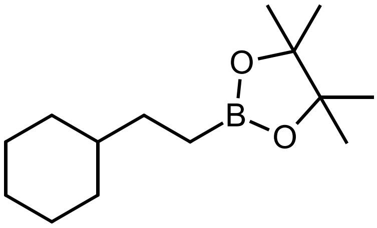

Vinylcyclohexane (114 µL, 1.13 mmol, 1 eq.); catalyst (1.25 mg, 2.8 µmol, 0.25 mol%); eluent: *n*-pentane; extracted with *n*-pentane; 155 mg (58 %) of a colorless oil.

^{1}H NMR (250 MHz, $CDCl_3$): δ = 1.79 – 1.55 (m, 4H), 1.35 – 1.04 (m, 19H), 0.87 (t, *J* = 6.1 Hz, 2H), 0.75 (t, *J* = 8.3 Hz, 2H) ppm.

^{13}C {^{1}H} NMR (63 MHz, $CDCl_3$): δ = 82.9, 40.0, 33.1, 31.4, 26.8, 26.5, 24.9, 8.14 ppm.

RT (GC): 13.85 min MS: 238.23 m/z $[M]^+$ (Method **B**)

These spectroscopic data correspond to reported data.[11]

<u>2-[2-(3-cyclohexen-1-yl)ethyl]-4,4,5,5-tetramethyl-1,3,2-dioxaborolane</u> (**4q**)

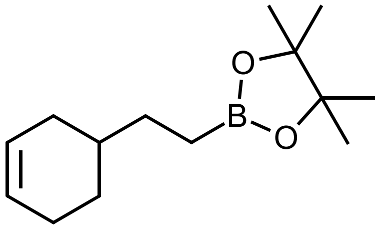

4-Vinylcyclohexene (146 µL, 1.13 mmol, 1 eq; catalyst (1.25 mg, 2.8 µmol, 0.25 mol%); eluent: *n*-pentane; extracted with *n*-pentane; 184 mg (69 %) of a colorless oil.

^{1}H NMR (400 MHz, CD_2Cl_2): δ = 5.64 (d, *J* = 2.0 Hz, 2H), 2.14 – 1.98 (m, 3H), 1.79 – 1.70 (m, 1H), 1.66 – 1.56 (m, 1H), 1.50 – 1.39 (m, 1H), 1.38 – 1.30 (m, 2H), 1.21 (s, 12H), 1.19 – 1.11 (m, 1H), 0.75 (t, *J* = 8.2 Hz, 2H) ppm.

^{13}C {^{1}H} NMR (101 MHz, $CDCl_3$): δ = 127.3, 127.0, 83.2, 36.2, 32.1, 31.2, 29.0, 25.8, 25.1, 8.7 ppm.

RT (GC): 14.17 min MS: 236.15 m/z $[M]^+$ (Method **B**)

These spectroscopic data correspond to reported data.[11]

<u>4,4,5,5-Tetramethyl-2-(3-phenoxypropyl)-1,3,2-dioxaborolane</u> (**4r**)

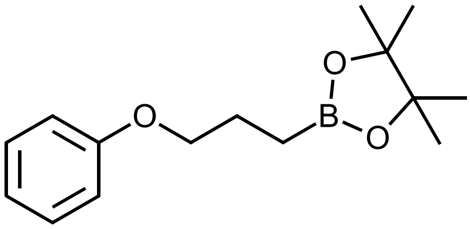

Allyl phenyl ether (154 µL, 1.13 mmol, 1 eq); catalyst (5 mg, 11.3 µmol, 1 mol%); eluent: *n*-pentane; extracted with *n*-pentane; 135 mg (46 %) of a colorless oil.

^{1}H NMR (400 MHz, $CDCl_3$): δ = 7.30 – 7.22 (m, 2H), 6.93 – 6.86 (m, 3H), 3.93 (t, *J* = 6.7 Hz, 2H), 1.91 – 1.80 (m, 2H), 1.23 (s, 12H), 0.88 (t, *J* = 7.9 Hz, 2H) ppm.

^{13}C {^{1}H} NMR (101 MHz, CD_2Cl_2): δ = 159.22, 129.32, 120.29, 114.47, 83.00, 69.50, 24.63, 23.79, 7.20 ppm.

RT (GC): 4.41 min MS: 227.13 m/z $[M]^+$

These spectroscopic data correspond to reported data.[15]

[3-(4,4,5,5-Tetramethyl-1,3,2-dioxaborolan-2-yl)propyl]aniline (**4s**)

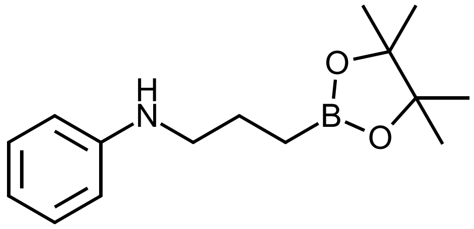

N-Allylaniline (153 µL, 1.13 mmol, 1 eq.); catalyst (5 mg, 11.3 µmol, 1 mol%); eluent: *n*-pentane; extracted with *n*-pentane; 222 mg (75 %) of a yellow oil.

^{1}H NMR (400 MHz, CD_2Cl_2): δ = 7.13 (t, J = 7.6 Hz, 2H), 6.72 – 6.52 (m, 3H), 3.82 (s, 1H), 3.15 – 3.03 (m, 2H), 1.70 (t, J = 7.2 Hz, 2H), 1.24 (s, 12H), 0.84 (t, J = 7.5 Hz, 2H) ppm.

^{13}C {^{1}H} NMR (101 MHz, CD_2Cl_2): δ = 149.2, 129.5, 117.1, 112.9, 83.5, 46.4, 25.1, 24.8, 9.2 ppm.

RT (GC): 6.79 min MS: 261.11 m/z $[M]^+$

These spectroscopic data correspond to reported data.[16]

2-(6-Chlorohexyl)-4,4,5,5-tetramethyl-1,3,2-dioxaborolane (**4t**)

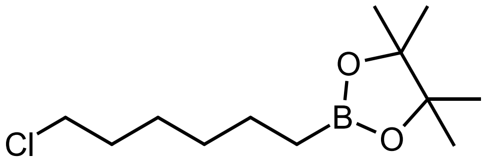

6-Chlorohexene (149 µL, 1.13 mmol, 1 eq.); catalyst (1.25 mg, 2.8 µmol, 0.25 mol%); eluent: *n*-pentane; extracted with *n*-pentane; 219 mg (79 %) of a colorless oil.

^{1}H NMR (400 MHz, $CDCl_3$): δ = 3.51 (t, J = 6.8 Hz, 2H), 1.82 – 1.69 (m, 2H), 1.47 – 1.36 (m, 4H), 1.36 – 1.27 (m, 2H), 1.23 (s, 12H), 0.76 (t, J = 7.6 Hz, 2H) ppm.

^{13}C {^{1}H} NMR (101 MHz, $CDCl_3$): δ = 83.0, 45.3, 32.7, 31.7, 26.8, 24.9, 23.9, 11.2 ppm.

RT (GC): 5.50 min MS: 246.06 m/z $[M]^+$

These spectroscopic data correspond to reported data.[11]

Trimethyl[3-(4,4,5,5-tetramethyl-1,3,2-dioxaborolan-2-yl)propyl]silane (**4u**)

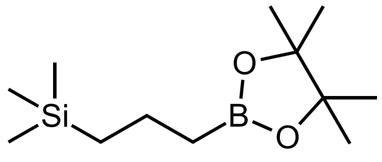

Allyltrimethylsilane (179 µL, 1.13 mmol, 1 eq); catalyst (5 mg, 11.3 µmol, 1 mol%); eluent: *n*-pentane; extracted with *n*-pentane; 130 mg (48 %) of a colorless liquid.

^{1}H NMR (400 MHz, $CDCl_3$): δ = 1.47 – 1.33 (m, 2H), 1.23 (s, 12H), 0.85 – 0.73 (m, 2H), 0.54 – 0.42 (m, 2H), -0.05 (s, 9H) ppm.

^{13}C {^{1}H} NMR (101 MHz, $CDCl_3$): δ = 83.0, 25.0, 20.2, 18.7, 15.6, -1.5 ppm.

RT (GC): 4.41 min MS: 227.13 m/z $[M]^+$

These spectroscopic data correspond to reported data.[11]

2-Dodecyl-4,4,5,5-tetramethyl-1,3,2-dioxaborolane (**4v**)

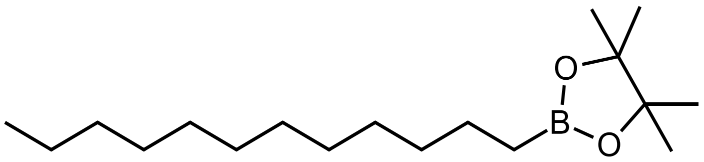

1-Dodecene (250 µL, 1.13 mmol, 1 eq.); catalyst (1.25 mg, 2.8 µmol, 0.25 mol%); eluent: *n*-pentane; extracted with *n*-pentane; 272 mg (82 %) of a colorless liquid.

^{1}H NMR (250 MHz, CD_2Cl_2): δ = 1.36 – 1.23 (m, 21H), 1.21 (s, 12H), 0.88 (t, *J* = 6.4 Hz, 2H), 0.71 (t, *J* = 7.2 Hz, 2H).

^{13}C {^{1}H} NMR (63 MHz, CD_2Cl_2): δ = 83.1, 32.8, 32.4, 30.1, 30.1, 30.0, 29.9, 29.8, 25.0, 24.5, 23.1, 14.3, 11.1 ppm.

RT (GC): 6.63 min MS: 296.41 m/z $[M]^+$

These spectroscopic data correspond to reported data.[15]

Substrate scope of terminal alkynes

Inside an argon flushed glovebox, a screw cap vial (8 mL) was charged with substrate (1.13 mmol, 1 equiv.), pinacolborane (246 µL, 1.50 mmol, 1.5 equiv.), catalyst (2.5 mg, 0.5 mol%) and THF (0.5 mL) and closed under argon atmosphere. The vial was transferred out of the glovebox and stirred for 24 hours at 70°C. The sample was allowed to reach room temperature, exposed to air and analyzed with GC-MS. The solvent was removed, and the residue purified by column chromatography.

2-[(*Z*)-1-Phenyl-2-(4,4,5,5-tetramethyl-1,3,2-dioxaborolan-2-yl)ethenyl]-4,4,5,5-tetramethyl-1,3,2-dioxaborolane (**7a**)

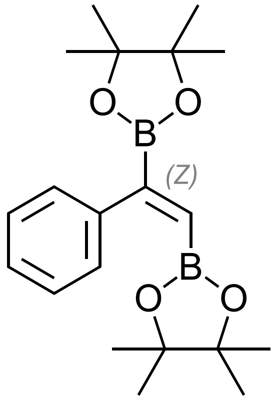

Phenylacetylene (124 µL, 1.13 mmol, 1 eq.); eluent: petroleum ether and diethyl ether (8:1); 143 mg (36%) of a slightly yellow liquid.
^{1}H NMR (400 MHz, $CDCl_3$): δ = 7.36 - 7.28 (m, 3H), 7.25 - 7.19 (m, 2H), 6.58 (s, 1H), 1.27 (s, 12H), 1.16 (s, 12H) ppm.
^{13}C {^{1}H} NMR (101 MHz, $CDCl_3$): δ = 142.4, 128.5, 127.7, 127.0, 84.0, 83.6, 24.9, 24.8 ppm. (C=C not detected)
RT (GC): 19.47 min MS: 356.15 m/z $[M]^+$ (Method **B**)
These spectroscopic data correspond to reported data.[17]

2-[(*Z*)-1-(*p*-Fluorophenyl)-2-(4,4,5,5-tetramethyl-1,3,2-dioxaborolan-2-yl)ethenyl]-4,4,5,5-tetramethyl-1,3,2-dioxaborolane (**7b**)

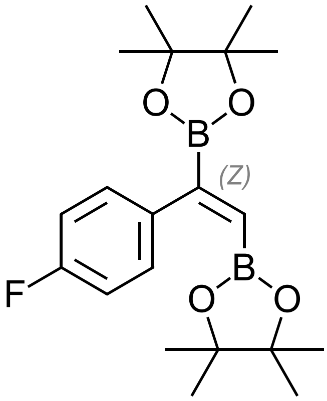

1-Fluoro-4-ethynylbenzene (136 mg, 1.13 mmol, 1 eq.); yield: 41 % (according to GC-MS). (*compound is highly unstable and decomposes even if stored under inert atmosphere and under reduced temperature)*
RT (GC): 19.24 min MS: 374.13 m/z $[M]^+$ (Method **B**)

2-[(*Z*)-1-(*p*-Chlorophenyl)-2-(4,4,5,5-tetramethyl-1,3,2-dioxaborolan-2-yl)ethenyl]-4,4,5,5-tetramethyl-1,3,2-dioxaborolane (**7c**)

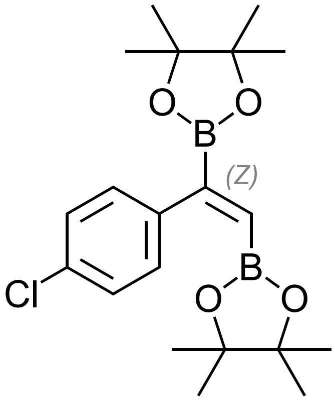

4-Ethynyltoluene (154 µL, 1.13 mmol, 1 eq); yield: 44% (according to GC-MS). (*compound is highly unstable and decomposes even if stored under inert atmosphere and under reduced temperature*)

RT (GC): 7.72 min MS: 390.11 m/z $[M]^+$

2-[(*Z*)-2-(4,4,5,5-Tetramethyl-1,3,2-dioxaborolan-2-yl)-1-(*p*-tolyl)ethenyl]-4,4,5,5-tetramethyl-1,3,2-dioxaborolane (**7d**)

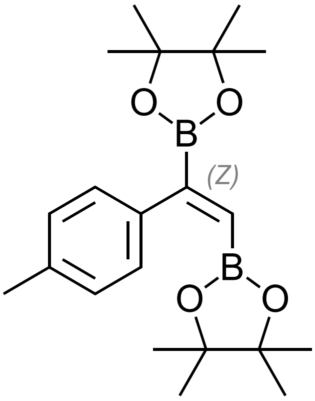

4-Ethynyltoluene (143 µL, 1.13 mmol, 1 eq.); eluent: petroleum ether and diethyl ether (8:1); 140 mg (34%) of a yellow solid.

^{1}H NMR (400 MHz, CD_2Cl_2): δ = 7.18 - 7.14 (m, 2H), 7.11 - 7.06 (m, 2H), 6.43 (s, 1H), 2.34 (s, 3H), 1.30 - 1.22 (m, 12H), 1.21 - 1.13 (m, 12H) ppm.

^{13}C {^{1}H} NMR (101 MHz, CD_2Cl_2): δ = 149.7, 140.0, 137.3, 128.9, 128.8, 84.5, 83.9, 25.1, 25.0, 21.4 ppm. (C-**C**(B)=C not detected)

RT (GC): 7.50 min MS: 370.14 m/z $[M]^+$

These spectroscopic data correspond to reported data.[17]

2-{(*Z*)-1-[*p*-(*tert*-Butyl)phenyl]-2-(4,4,5,5-tetramethyl-1,3,2-dioxaborolan-2-yl)ethenyl}-4,4,5,5-tetramethyl-1,3,2-dioxaborolane (**7e**)

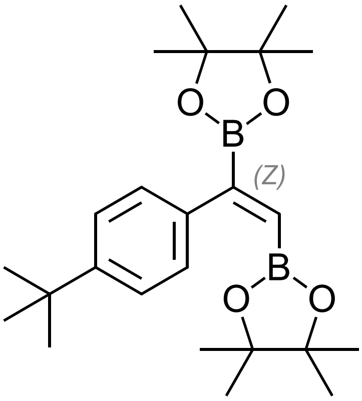

4-*tert*-Butylphenylacetylene (204 µL, 1.13 mmol, 1 eq.); eluent: petroleum ether and diethyl ether (8:1); 147 mg (32%) of a yellow solid.

^{1}H NMR (400 MHz, CD_2Cl_2): δ = 7.33 - 7.28 (m, 2H), 7.21 - 7.16 (m, 2H), 6.44 (s, 1H), 1.32 (s, 9H), 1.28 (s, 12H), 1.15 (s, 12H) ppm.

^{13}C {^{1}H} NMR (101 MHz, CD_2Cl_2): δ = 150.5, 149.6, 140.1, 128.6, 125.0, 84.5, 83.9, 34.9, 31.7, 25.1, 25.0 ppm. (C-**C**(B)=C not detected)

RT (GC): 7.93 min HRMS (APCI): m/z calculated for $C_{21}H_{33}B_2O_4$ $[M+H]^+$: 413.3028, found: 413.3035 m/z

2-[(*Z*)-2-(4,4,5,5-Tetramethyl-1,3,2-dioxaborolan-2-yl)-1-(*m*-tolyl)ethenyl]-4,4,5,5-tetramethyl-1,3,2-dioxaborolane (**7f**)

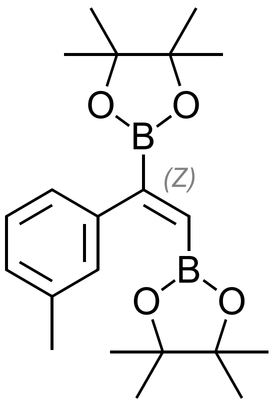

3-Ethynyltoluene (146 µL, 1.13 mmol, 1 eq.); eluent: petroleum ether and diethyl ether (8:1); 143 mg (34%) of a yellow liquid.

^{1}H NMR (400 MHz, CD_2Cl_2): δ = 7.44 - 7.26 (m, 1H), 7.26 - 7.10 (m, 1H), 7.10 - 6.89 (m, 2H), 6.45 (s, 1H), 2.32 (s, 3H), 1.28 (s, 12H), 1.16 (s, 12H) ppm.

^{13}C {^{1}H} NMR (101 MHz, CD_2Cl_2): δ = 149.9, 142.9, 137.6, 129.6, 128.2, 128.0, 126.0, 84.5, 83.9, 25.1, 25.0, 21.7 ppm. (C-**C**(B)=C not detected)

RT (GC): 7.44 min HRMS (APCI): m/z calculated for $C_{21}H_{33}B_2O_4$ $[M+H]^+$: 371.2559, found: 371.2562 m/z

2-[(*Z*)-1-(*o*-Chlorophenyl)-2-(4,4,5,5-tetramethyl-1,3,2-dioxaborolan-2-yl)ethenyl]-4,4,5,5-tetramethyl-1,3,2-dioxaborolane (**7g**)

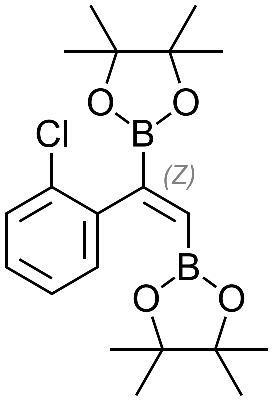

1-Chloro-2-ethynylbenzene (139 µL, 1.13 mmol, 1 eq.); eluent: petroleum ether and diethyl ether (8:1); 160 mg (37%) of an orange liquid.

^{1}H NMR (400 MHz, CD_2Cl_2): δ = 7.35 - 7.29 (m, 2H), 7.24 - 7.15 (m, 2H), 7.14 - 7.09 (m, 1H), 6.55 (s, 1H), 1.26 (s, 12H), 1.08 (s, 12H) ppm.

^{13}C {^{1}H} NMR (101 MHz, CD_2Cl_2): δ = 142.3, 132.8, 131.0, 129.1, 128.7, 126.8, 84.6, 83.9, 25.0, 24.9 ppm. (C=C not detected)

RT (GC): 7.46 min HRMS (APCI): m/z calculated for $C_{20}H_{30}B_2ClO_4$ $[M+H]^+$: 391.2013, found: 391.2017 m/z

2-[(*Z*)-1-(*p*-Methoxyphenyl)-2-(4,4,5,5-tetramethyl-1,3,2-dioxaborolan-2-yl)ethenyl]-4,4,5,5-tetramethyl-1,3,2-dioxaborolane (**7h**)

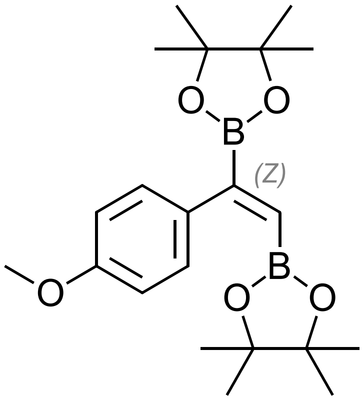

4-Ethynylanisole (147 µL, 1.13 mmol, 1 eq.); yield: 46% (according to GC-MS). (*compound is highly unstable and decomposes even if stored under inert atmosphere and under reduced temperature*)

RT (GC): 7.89 min MS: 386.13 m/z $[M]^+$

2-[(*Z*)-1-(6-Methoxy-2-naphthyl)-2-(4,4,5,5-tetramethyl-1,3,2-dioxaborolan-2-yl)ethenyl]-4,4,5,5-tetramethyl-1,3,2-dioxaborolane (**7i**)

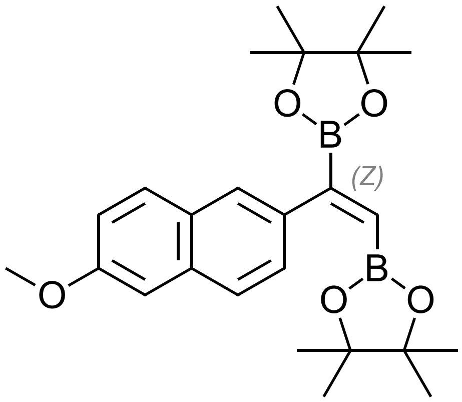

2-Ethynyl-6-methoxynaphthalene (206 mg, 1.13 mmol, 1 eq.); yield: 33% (according to GC-MS). (*compound is highly unstable and decomposes even if stored under inert atmosphere and under reduced temperature)*

RT (GC): 9.61 min MS: 436.17 m/z $[M]^+$

2-[(*Z*)-2-(4,4,5,5-Tetramethyl-1,3,2-dioxaborolan-2-yl)-1-octenyl]-4,4,5,5-tetramethyl-1,3,2-dioxaborolane (**7j**)

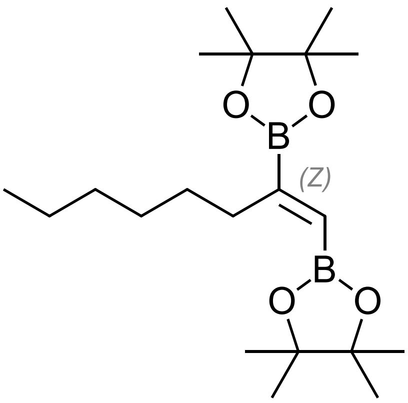

1-Octyne (167 µL, 1.13 mmol, 1 eq.); eluent: petroleum ether and diethyl ether (12:1); 144 mg (35%) of a colorless liquid.

^{1}H NMR (400 MHz, C_6D_6): δ = 6.97 (s, 1H), 3.00 (t, *J* = 7.6 Hz, 2H), 1.84 - 1.71 (m, 2H), 1.60 - 1.47 (m, 2H), 1.44-1.26 (m, 4H), 1.08 - 1.05 (m, 12H), 1.01 (s, 12H), 0.90 (t, *J* = 7.1 Hz, 3H) ppm.

^{13}C {^{1}H} NMR (101 MHz, C_6D_6): δ = 155.1, 83.5, 82.7, 34.2, 32.3, 31.4, 29.8, 24.9, 24.6, 23.1, 14.4 ppm. (C-**C**(B)=C not detected)

RT (GC): 18.67 min MS: 363.16 m/z $[M-H]^+$ (Method **B**)

These spectroscopic data correspond to reported data.[18]

2-[(*Z*)-5-Methyl-2-(4,4,5,5-tetramethyl-1,3,2-dioxaborolan-2-yl)-1-hexenyl]-4,4,5,5-tetramethyl-1,3,2-dioxaborolane (**7k**)

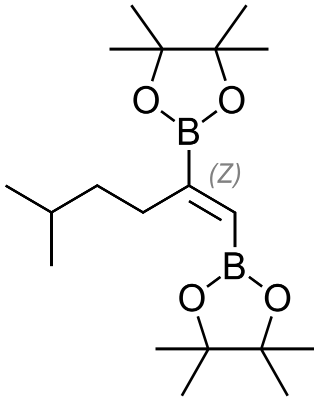

5-Methylhexyne (149 µL, 1.13 mmol, 1 eq.); eluent: petroleum ether and diethyl ether (12:1); 113 mg (29%) of a colorless oil.

^{1}H NMR (400 MHz, CD_2Cl_2): δ = 6.08 (s, 1H), 2.42 (t, *J* = 7.8 Hz, 2H), 1.59 – 1.45 (m, 1H), 1.27 – 1.19 (m, 26H), 0.89 (d, *J* = 6.8 Hz, 6H) ppm.

^{13}C NMR (101 MHz, CD_2Cl_2): δ = 84.0, 83.4, 40.6, 32.2, 28.7, 25.2, 25.1, 23.0 ppm. (C=C not detected)

RT (GC): 17.49 min MS: 349.13 m/z $[M-H]^+$ (Method **B**)

These spectroscopic correspond to reported data.[19]

2-[(*Z*)-6-Chloro-2-(4,4,5,5-tetramethyl-1,3,2-dioxaborolan-2-yl)-1-hexenyl]-4,4,5,5-tetramethyl-1,3,2-dioxaborolane (**7l**)

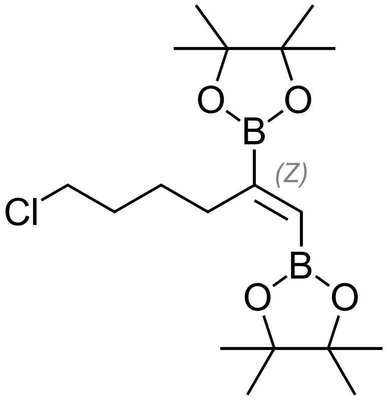

6-Chlorohexyne (137 µL, 1.13 mmol, 1 eq.); eluent: petroleum ether and diethyl ether (12:1); 129 mg (31%) of a colorless solid.

^{1}H NMR (400 MHz, CD_2Cl_2): δ = 6.16 (s, 1H), 3.56 (t, *J* = 6.9 Hz, 2H), 2.50 – 2.41 (m, 2H), 1.81 – 1.69 (m, 2H), 1.57 – 1.46 (m, 2H), 1.26 – 1.23 (m, 24H) ppm.

^{13}C NMR (101 MHz, CD_2Cl_2): δ = 84.1, 83.5, 45.8, 33.0, 32.8, 28.1, 25.2, 25.1 ppm. (C=C not detected)

RT (GC): 7.30 min MS: 370.11 m/z $[M]^+$

These spectroscopic correspond to reported data.[19]

2-[(*Z*)-2-(4,4,5,5-Tetramethyl-1,3,2-dioxaborolan-2-yl)-1-hexenyl]-4,4,5,5-tetramethyl-1,3,2-dioxaborolane (**7m**)

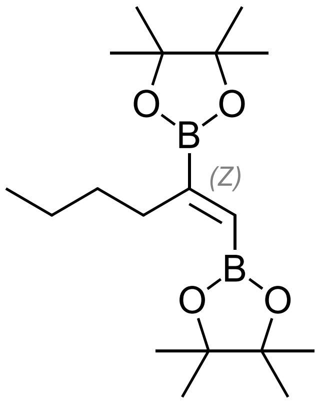

1-Hexyne (130 µL, 1.13 mmol, 1 eq.); eluent: petroleum ether and diethyl ether (12:1); 49 mg (13%) of a colorless liquid.

^{1}H NMR (400 MHz, CD_2Cl_2): δ = 6.11 (s, 1H), 2.42 (t, *J* = 7.1 Hz, 2H), 1.38 – 1.27 (m, 4H), 1.24 (d, *J* = 1.6 Hz, 24H), 0.89 (t, *J* = 7.1 Hz, 3H).

^{13}C NMR (101 MHz, CD_2Cl_2): δ = 133.4, 84.0, 83.4, 33.9, 33.5, 25.2, 25.1, 23.1, 14.4 ppm. (C-**C**(B)=C not detected)

RT (GC): 17.11 min HRMS (APCI): m/z calculated for $C_{18}H_{35}B_2O_4$ $[M+H]^+$: 337.2715, found: 337.2721 m/z

6. Computational details

The computational results presented have been achieved in part using the Vienna Scientific Cluster (VSC). All calculations were performed using the GAUSSIAN 09 software package[20] without symmetry constraints. The optimized geometries were obtained with the the PBE0 functional. That functional uses a hybrid generalized gradient approximation (GGA), including 25 % mixture of Hartree-Fock[21] exchange with DFT[22] exchange-correlation, given by Perdew, Burke and Ernzerhof functional (PBE).[23] The basis set used for the geometry optimizations (basis b1) consisted of the Stuttgart/Dresden ECP (SDD) basis set[24] to describe the electrons of iron, and a standard 6-31G(d,p) basis set[25] for all other atoms. Transition state optimizations were performed with the Synchronous Transit-Guided Quasi-Newton Method (STQN) developed by Schlegel *et al*,[26] following extensive searches of the Potential Energy Surface. Frequency calculations were performed to confirm the nature of the stationary points, yielding one imaginary frequency for the transition states and none for the minima. Each transition state was further confirmed by following its vibrational mode downhill on both sides and obtaining the minima presented on the energy profiles. The electronic energies (E_{b1}) obtained at the PBE0/b1 level of theory were converted to free energy at 298.15 K and 1 atm (G_{b1}) by using zero point energy and thermal energy corrections based on structural and vibration frequency data calculated at the same level.

Single point energy calculations were performed on the geometries optimized at the PBE0/b1 level, using the M06 functional and a standard 6-311++G(d,p) basis set.[27] The M06 functional is a hybrid meta-GGA functional developed by Truhlar and Zhao,[28] and it was shown to perform very well for the kinetics of transition metal molecules, providing a good description of weak and long range interactions.[29] Solvent effects (THF) were considered in *all* calculations (PBE0/b1 geometry optimizations included) using the Polarizable Continuum Model (PCM) initially devised by Tomasi and coworkers[30] with radii and non-electrostatic terms of the SMD solvation model, developed by Truhlar *et al*.[31] The free energy values presented (G_{b2}) were derived from the electronic energy values obtained at the M06/6-311++G(d,p)//PBE0/b1 level (E_{b2}) according to the following expression: $G_{b2} = E_{b2} + G_{b1} – E_{b1}$.

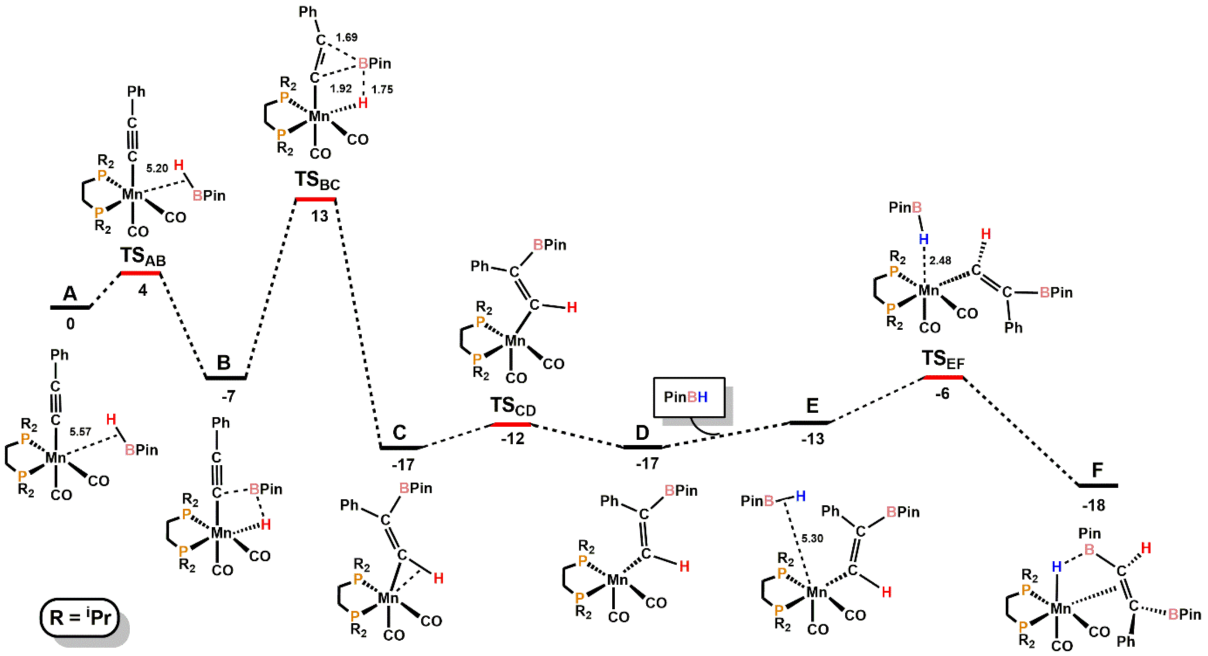

Figure S1. Energy Profile of the *trans*1-2-diboration of phenylacetylene (Intermediates **A-F**).

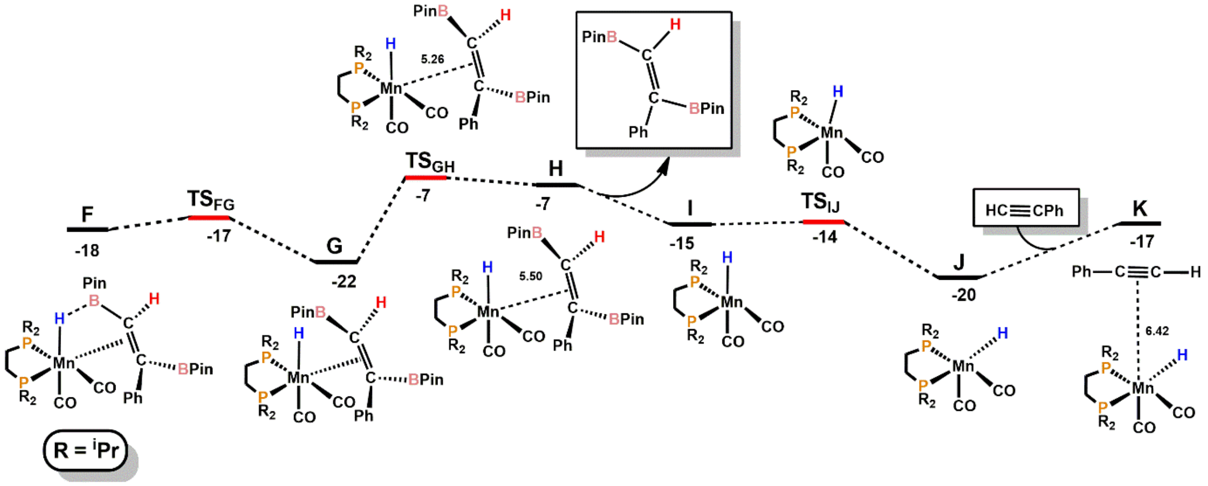

Figure S2. Energy Profile of the *trans*1-2-diboration of phenylacetylene (Intermediates **F-K**).

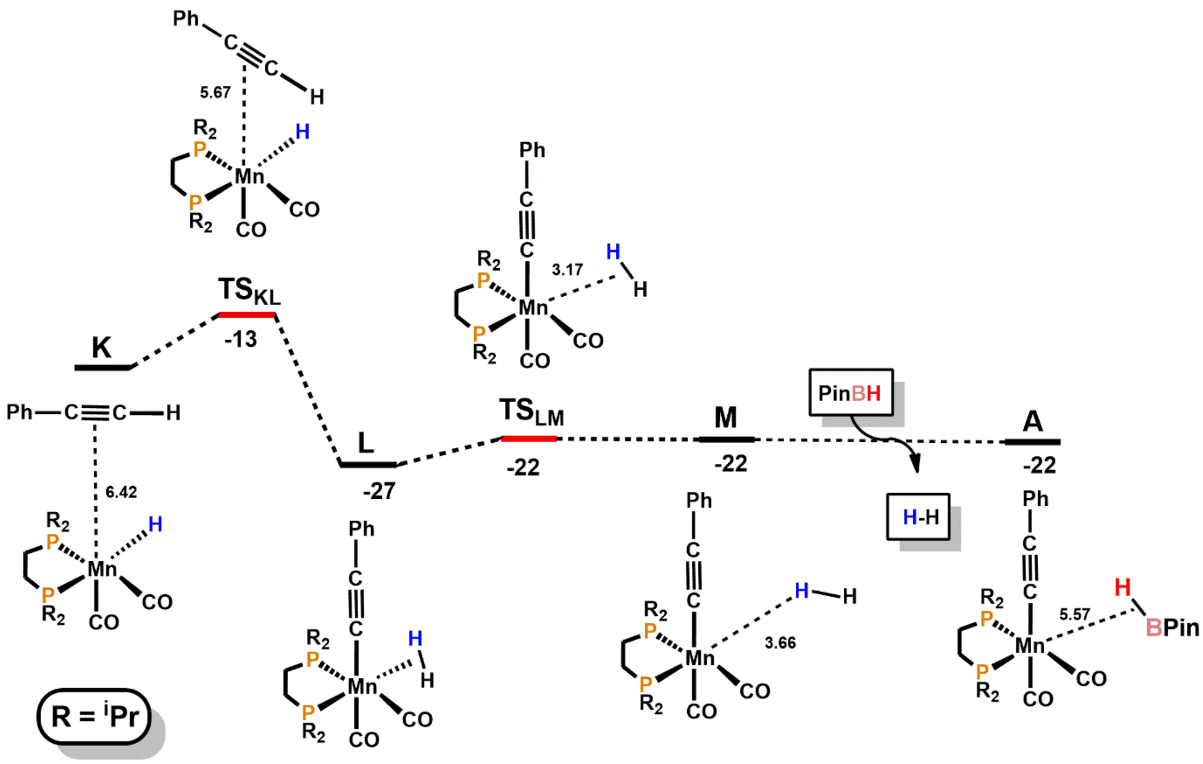

Figure S3. Energy Profile of the *trans*1-2-diboration of phenylacetylene (Intermediates **K-A**).

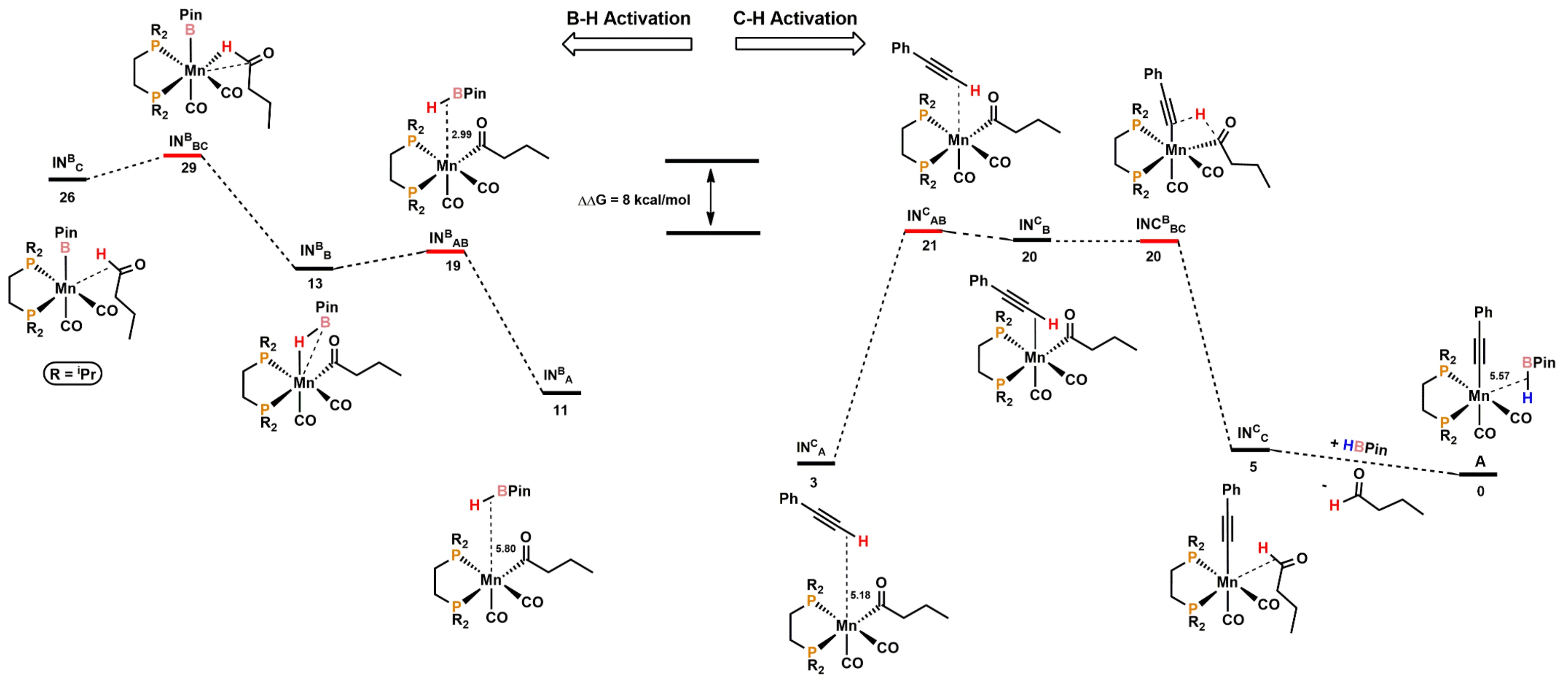

Figure S4. Energy Profile for the catalyst activation: B-H *vs.* C-H bond activation.

7. References

(1) S. Weber, B. Stöger, L. F. Veiros, K. Kirchner, *ACS Catal.* **2019**, *9*, 9715-9720.

(2) J. A. Garduño, A. Arévalo, M. Flores-Alamo, J. J. García, *Catal. Sci. Technol.* **2018**, *8*, 2606-2016.

(3) J. A. Garduño, J.J. García, *ACS Catal.* **2018**, *9*, 392-401.

(4) N. Gorgas, L. G. Alves, B. Stöger, A.M. Martins, L. F. Veiros, K. Kirchner, *J. Am. Chem. Soc.* **2017**,*139*, 8130-8133.

(5) J. W. Clay, T.J. Rettenmaier, R. Snelling, W. Bryks, J. Banwell, W.T. Wipke, B. Singarm, *J. Org. Chem.* **2011**, *76*, 9602-9610.

(6) D. J. Lippincott, R.T.H. Linstadt, M.R. Maser, F. Gallou, B. H. Lipshutz, *Org. Lett.* **2018**, *20*, 4719-4722.

(7) Bruker computer programs: APEX3, SAINT and SADABS (Bruker AXS Inc., Madison, WI, **2020**).

(8) G. M. Sheldrick, *Acta Crystallogr.* **2015**, *A71*, 3–8.

(9) A. L. Spek, *Acta Crystallogr.* **2009**, *D65*, 148–155.

(10) C. F. Macrae, P. R. Edgington, P. McCabe, E. Pidcock, G. P. Shields, R. Taylor, M. Towler and J. van de Streek, *J. Appl. Cryst.* **2006**, *39*, 453–457.

(11) L. Zhang, Z. Zuo, X. Leng, Z. Huang, *Angew. Chem. Int. Ed.* **2014**, *53*, 2696–2700.

(12) G. Zhang, J. Wu, M. Wang, H. Zeng, J. Cheng, M. C. Neary, S. Zheng *Eur. J. Org. Chem.* **2017**, 5814–5818.

(13) S. Hong, M. Liu, W. Zhang, Q. Zeng, W. Deng *Tetrahedron Lett.* **2015**, *56*, 2297–2302.

(14) A. Bismuto, M. J. Cowley, S. P. Thomas *ACS Catal.* **2018**, *8*, 2001–2005.

(15) Q. Liu, J. Hong, B. Sun, G. Bai, F. Li, G. Liu, Y. Yang, F. Mo *Org. Lett.* **2019**, *21*, 6597–6602.

(16) A. D. Ibrahim, S. W. Entsminger, A. R. Fout *ACS Catal.* **2017**, *7*, 3730–3734.

(17) Q. Chen, J. Zhao, Y. Ishikawa, N. Asao, Y. Yamamoto, T. Jin *Org. Lett.* **2013**, *15*, 5766–5769.

(18) J. Takaya, N. Kirai, N. Iwasawa *J. Am. Chem. Soc.* **2011**, *133*, 12980–12983.

(19) A. Yoshimura, Y. Takamachi, L.-B. Han, A. Ogawa *Chem. Eur. J.* **2015**, *21*, 13930–13933.

(20) Gaussian 09, Revision A.02, M. J. Frisch, G. W. Trucks, H. B. Schlegel, G. E. Scuseria, M. A. Robb, J. R. Cheeseman, G. Scalmani, V. Barone, B. Mennucci, G. A. Petersson, H. Nakatsuji, M. Caricato, X. Li, H.P. Hratchian, A. F. Izmaylov, J. Bloino, G. Zheng,

J.L. Sonnenberg, M. Hada, M. Ehara, K. Toyota, R. Fukuda, J. Hasegawa, M. Ishida, T. Nakajima, Y. Honda, O. Kitao, H. Nakai, T. Vreven, Jr. J. A. Montgomery, J. E. Peralta, F. Ogliaro, M. Bearpark, J. J. Heyd, E. Brothers, K. N. Kudin, V. N. Staroverov, R. Kobayashi, J. Normand, K. Raghavachari, A. Rendell, J. C. Burant, S. S. Iyengar, J. Tomasi, M. Cossi, N. Rega, J. M. Millam, M. Klene, J. E. Knox, J. B. Cross, V. Bakken, C. Adamo, J. Jaramillo, R. Gomperts, R. E. Stratmann, O. Yazyev, A. J. Austin, R. Cammi, C. Pomelli, J. W. Ochterski, R. L. Martin, K. Morokuma, V. G. Zakrzewski, G. A. Voth, P. Salvador, J. J. Dannenberg, S. Dapprich, A. D. Daniels, Ö. Farkas, J. B. Foresman, J. V. Ortiz, J. Cioslowski, D. J. Fox Gaussian, Inc., Wallingford CT, **2009**.

(21) W. J. Hehre, L. Radom, P. vR. Schleyer, J. A. Pople in *Ab Initio Molecular Orbital Theory*, John Wiley & Sons, NY, (1986).

(22) R. G. Parr, W. Yang in *Density Functional Theory of Atoms and Molecules*; Oxford University Press: New York, (1989).

(23) a) J. P. Perdew, K. Burke, M. Ernzerhof *Phys. Rev. Lett.* **1997**, *78*, 1396; (b) J. P. Perdew *Phys. Rev. B* **1986**, *33*, 8822.

(24) a) U. Haeusermann, M. Dolg, H. Stoll, H. Preuss *Mol. Phys.* **1993**, *78*, 1211-1224; b) W. Kuechle, M. Dolg, H. Stoll, H. Preuss *J. Chem. Phys.* **1994**, *100*, 7535-7542; c) T. Leininger, A. Nicklass, H. Stoll, M. Dolg, P. J. Schwerdtfeger *Chem. Phys.* **1996**, *105*, 1052-1059.

(25) a) A. D. McLean, G.S Chandler *J. Chem. Phys.* **1980**, *7*2, 5639-5648.; b) R. Krishnan, J. S. Binkley, R. Seeger, J. A. Pople. *J. Chem. Phys.* **1980**, *7*2, 650-654; c) A. J. H. Wachters *J. Chem. Phys.* **1970**, *52*, 1033-1036; d) P. J. Hay *J. Chem. Phys.* **1977**, *66*, 4377-4384; e) K. Raghavachari, G. W. Trucks *J. Chem. Phys.* **1989**, *91*, 1062-1065; f) Jr. R. C. Binning, L. A. Curtiss *J. Comp. Chem.*, **1990**, *11*, 1206; g) M. P. McGrath, L. Radom *J. Chem. Phys.* **1991**, *94*, 511-516.

(26) a) C. Peng, P. Y. Ayala, H. B. Schlegel, M. J. Frisch *J. Comp. Chem.* **1996**, *17*, 49-56; b) C. Peng, H. B. Schlegel *Israel J. Chem.* **1993**, *33*, 449-454.

(27) a) A. D. McLean, G.S Chandler *J. Chem. Phys.* **1980**, *7*2, 5639-5648.; b) R. Krishnan, J. S. Binkley, R. Seeger, J. A. Pople. *J. Chem. Phys.* **1980**, *7*2, 650-654; c) A. J. H. Wachters *J. Chem. Phys.* **1970**, *52*, 1033-1036; d) P. J. Hay *J. Chem. Phys.* **1977**, *66*, 4377-4384; e) K. Raghavachari, G. W. Trucks *J. Chem. Phys.* **1989**, *91*, 1062-1065; f) Jr. R. C. Binning, L. A. Curtiss *J. Comp. Chem.*, **1990**, *11*, 1206; g) M. P. McGrath, L. Radom *J. Chem. Phys.* **1991**, *94*, 511-516; h) T. Clark, J. Chandrasekhar, G.W.

Spitznagel, P. v.R. Schleyer *Comp. Chem.* **1983**, *4*, 294-301; i) M. J. Frisch, J. A. Pople, J. S. Binkley *J. Chem. Phys.* **1984**, *80*, 3265-3269.

(28) Y. Zhao, D. G. Truhlar *Theor. Chem. Acc.*, **2008**, *120*, 215-241.

(29) a) Y. Zhao, D. G. Truhlar *Acc, Chem. Res.* **2008**, *41*, 157-167; b) Y. Zhao, D. G. Truhlar *Chem. Phys. Lett.* **2011**, *502*, 1-13.

(30) a) M. T. Cancès, B. Mennucci, J. A. Tomasi *J. Chem. Phys.* **1997**, *107*, 3032-3041; b) M. Cossi, V. Barone, B. Mennucci, J. Tomasi *Chem. Phys. Lett.* **1998**, *286*, 253-260; c) B. Mennucci, J. Tomasi *J. Chem. Phys.* **1997**, *106*, 5151-5158; d) J. Tomasi, B. Mennucci, R. Cammi *Chem. Rev.* **2005**, *105*, 2999-3094.

(31) A. V. Marenich, C. J. Cramer, D. G. Truhlar *J. Phys. Chem. B,* **2009**, *113*, 6378-6396.

8. NMR Spectra

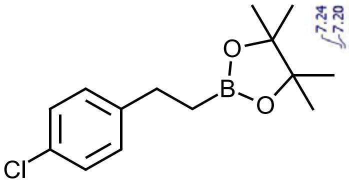

4a

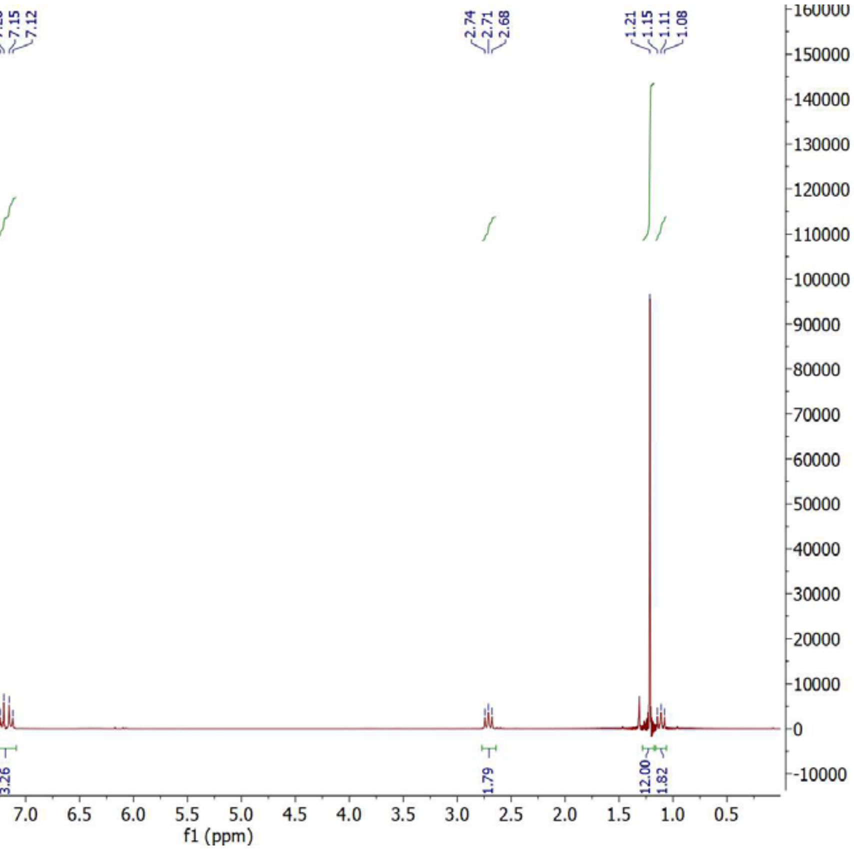

4a

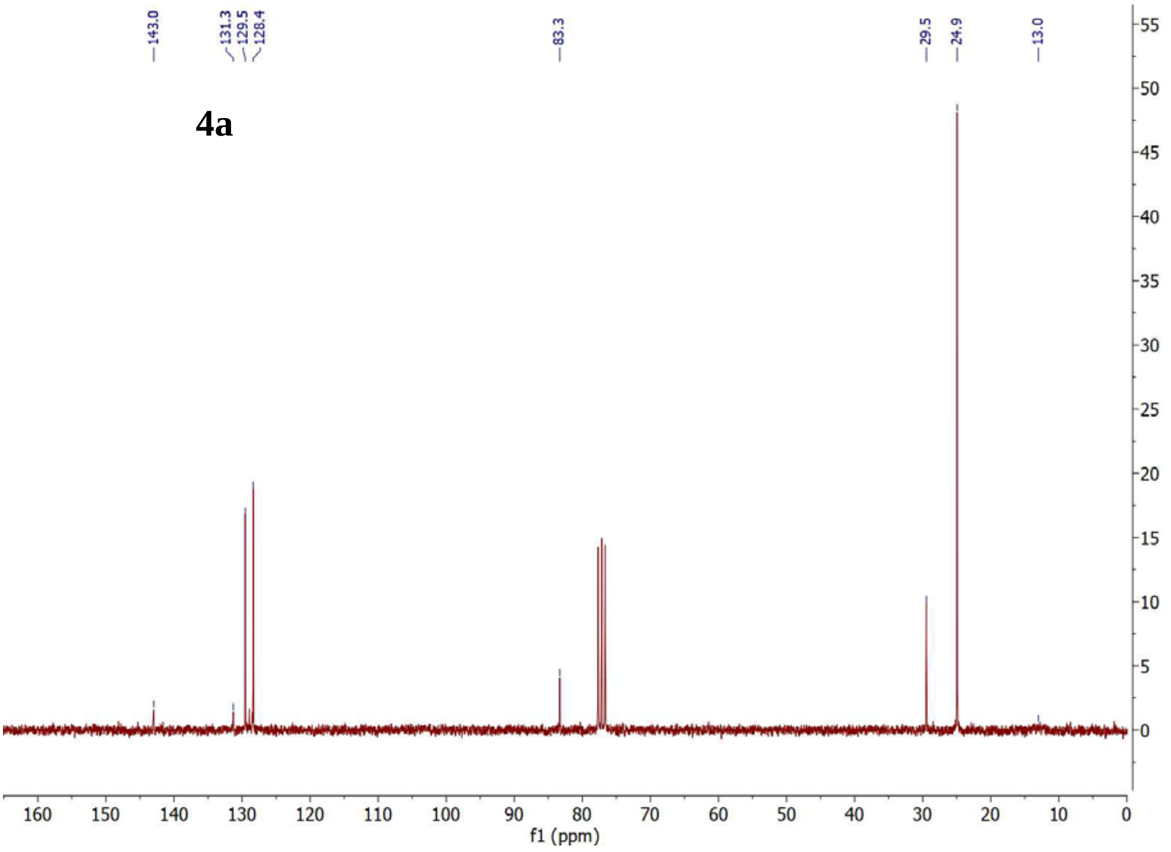

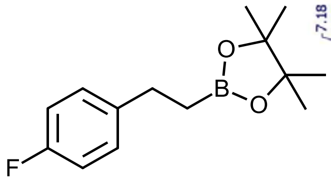

4b

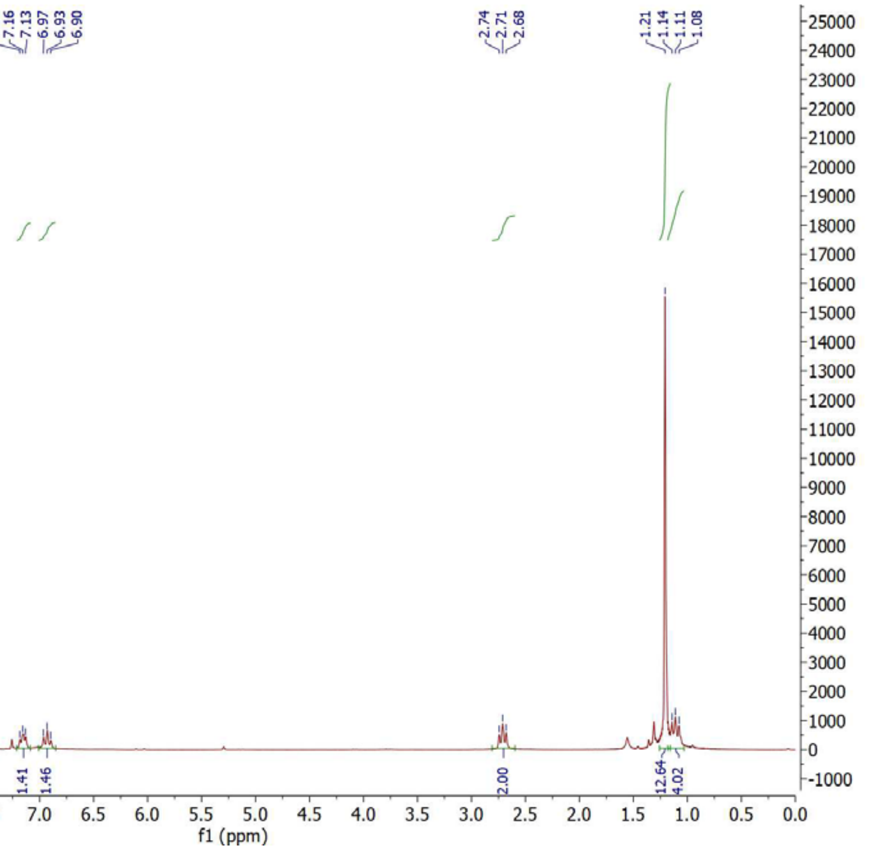
7.18 7.16 7.13 6.97 6.93 6.90
2.74 2.71 2.68
1.21 1.14 1.11 1.08
1.41 1.46 2.00 12.64 4.02
f1 (ppm)

4b

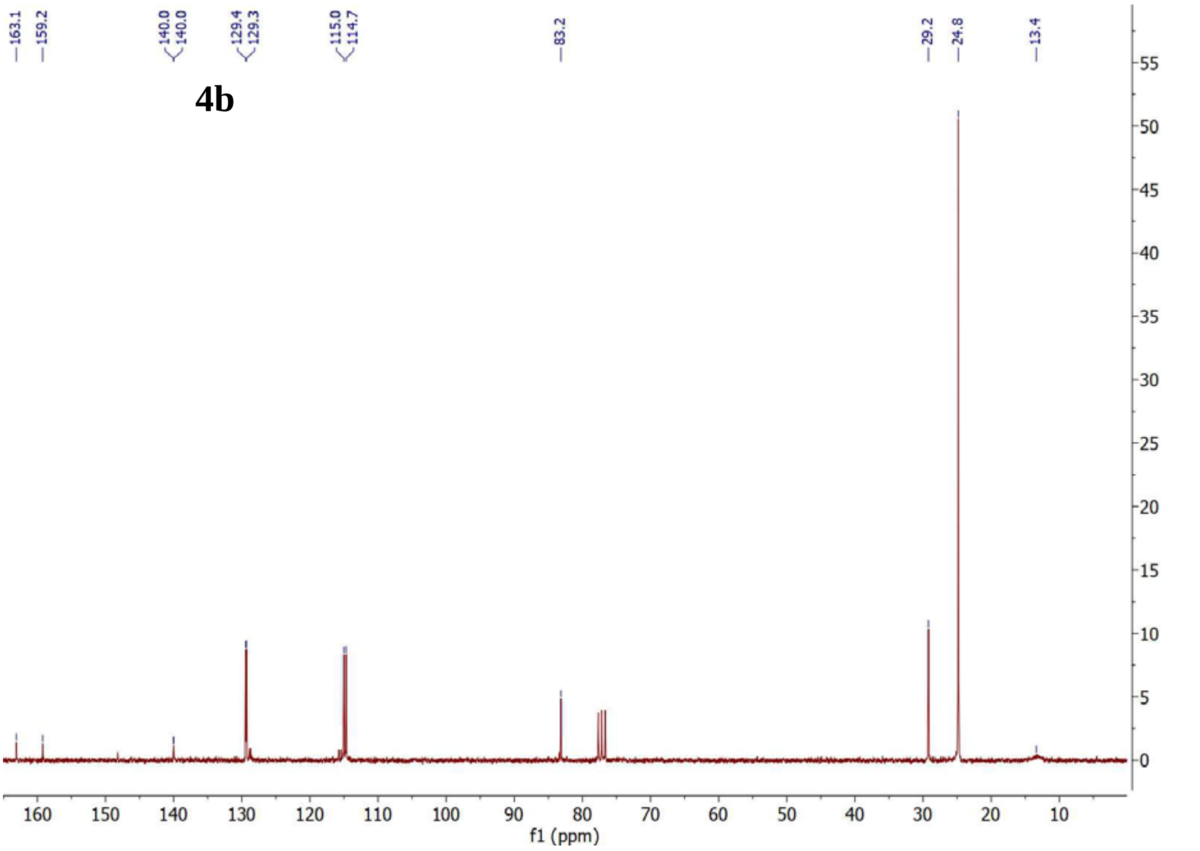
163.1 159.2 140.0 140.0 129.4 129.3 115.0 114.7 83.2 29.2 24.8 13.4
f1 (ppm)

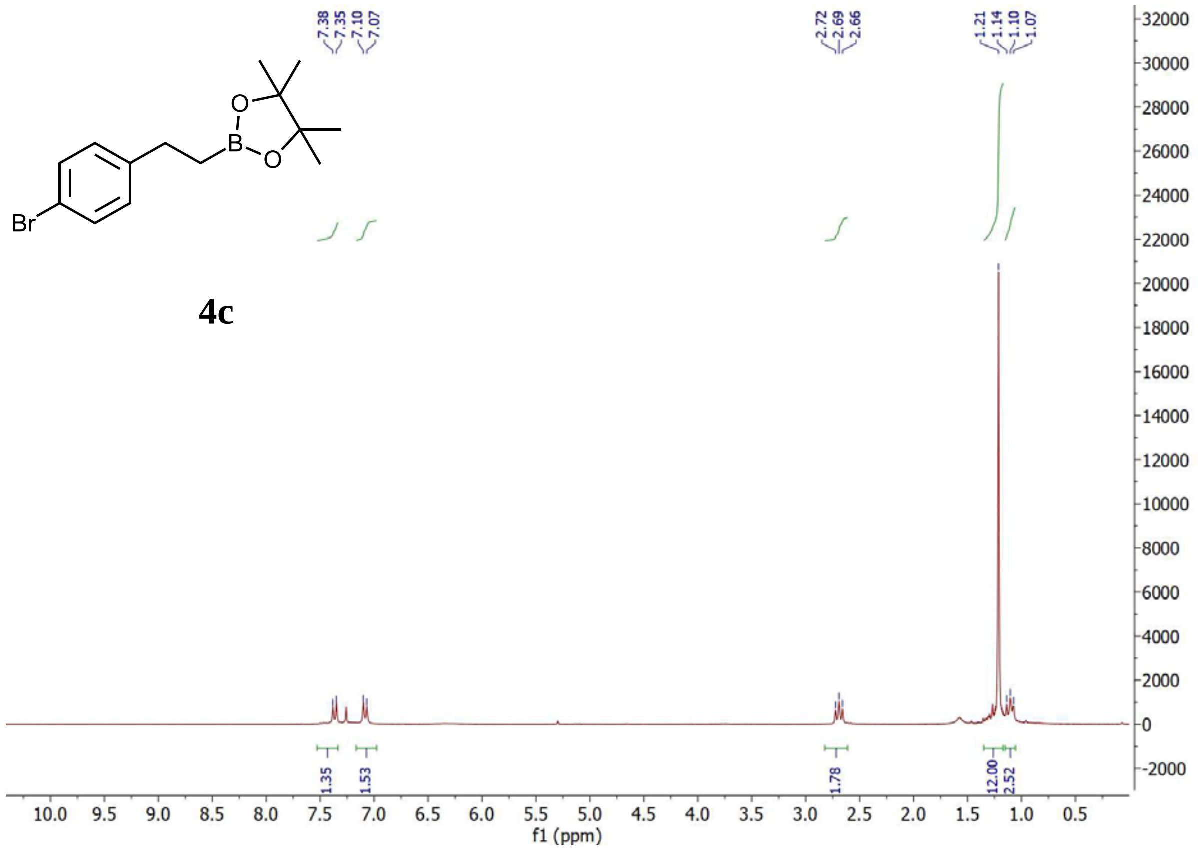
Br
B
O
O
7.38
7.35
7.10
7.07
2.72
2.69
2.66
1.21
1.14
1.10
1.07
1.35
1.53
1.78
12.00
2.52
f1 (ppm)

4c

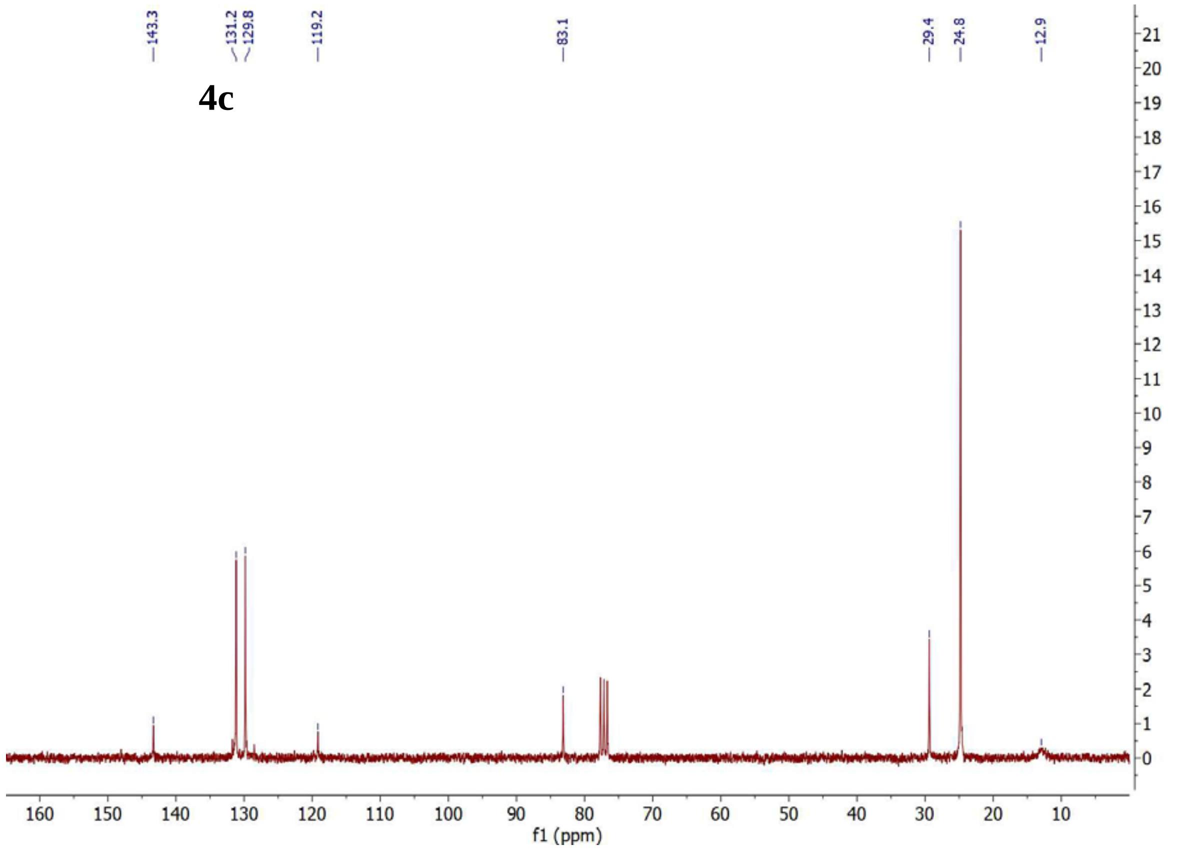
143.3
131.2
129.8
119.2
83.1
29.4
24.8
12.9
f1 (ppm)

4c

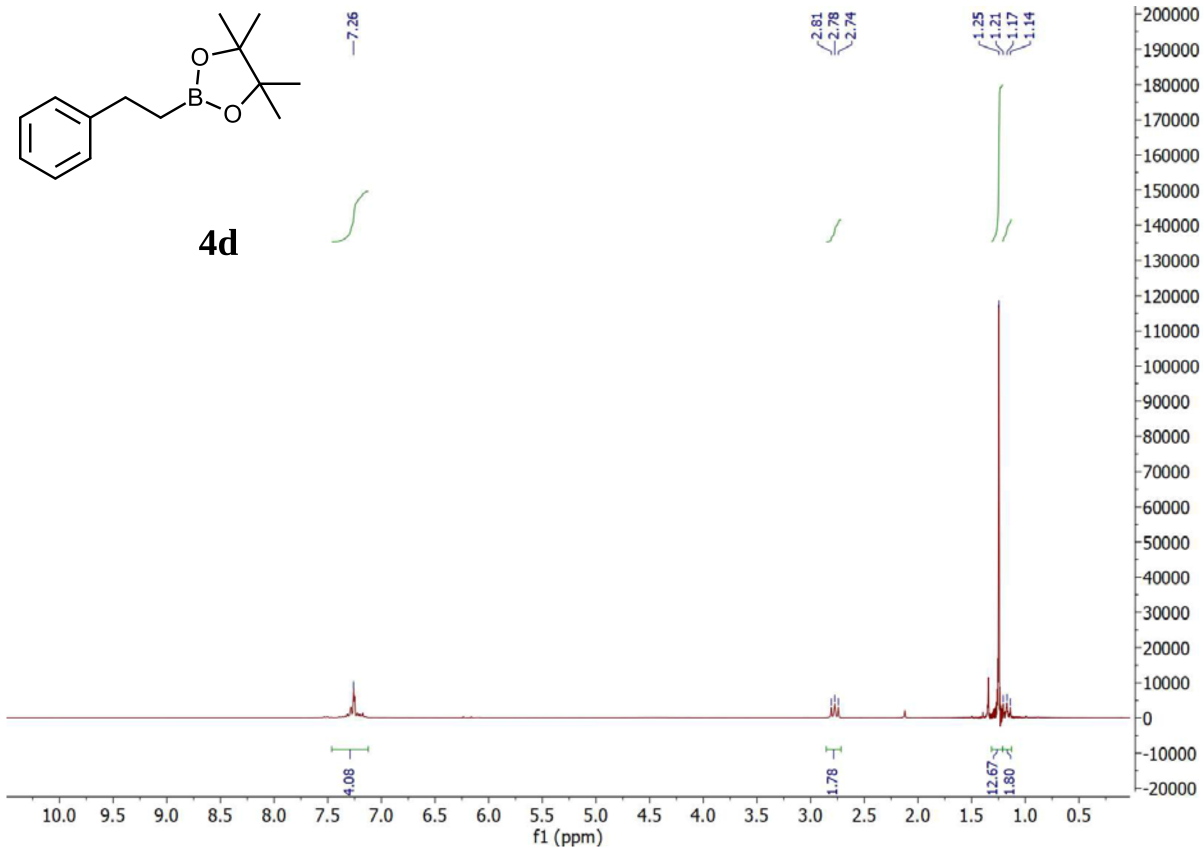
4d
7.26
2.81
2.78
2.74
1.25
1.21
1.17
1.14
4.08
1.78
12.67
1.80
f1 (ppm)

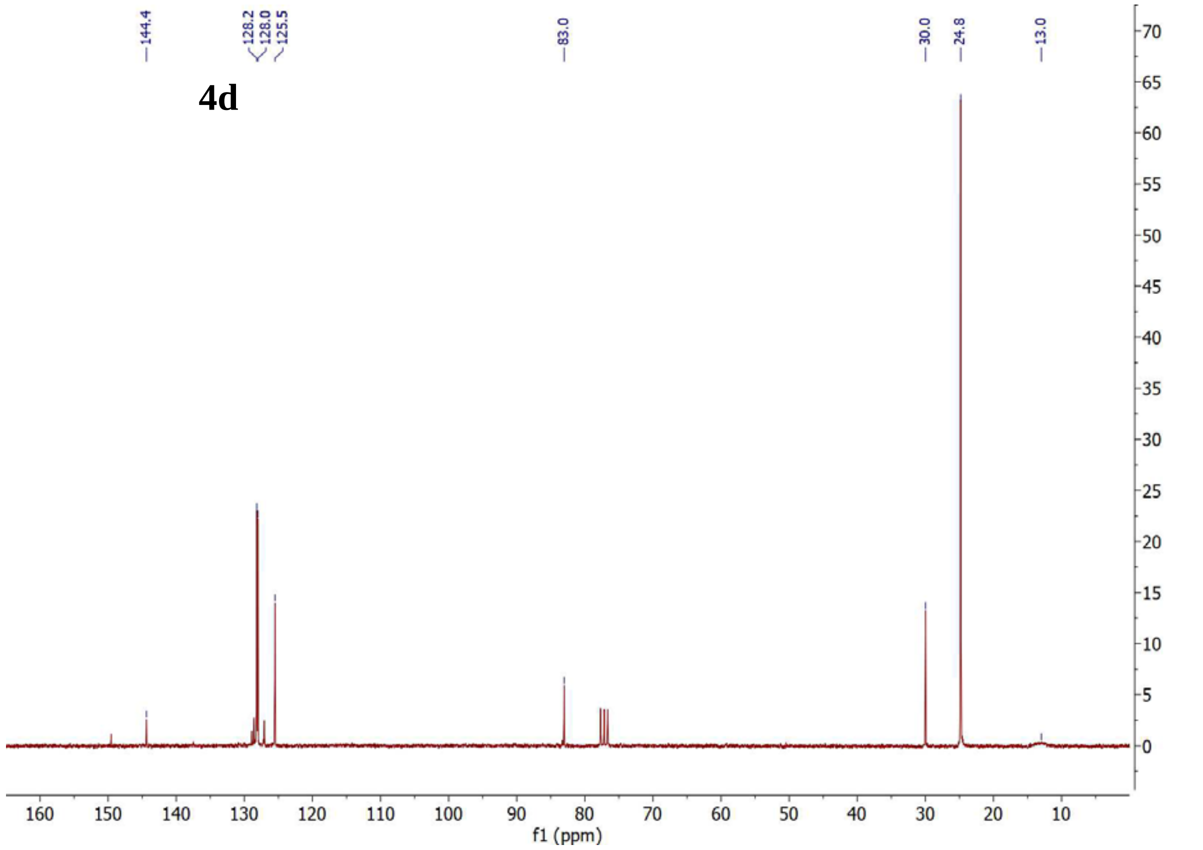
4d
144.4
128.2
128.0
125.5
83.0
30.0
24.8
13.0
f1 (ppm)

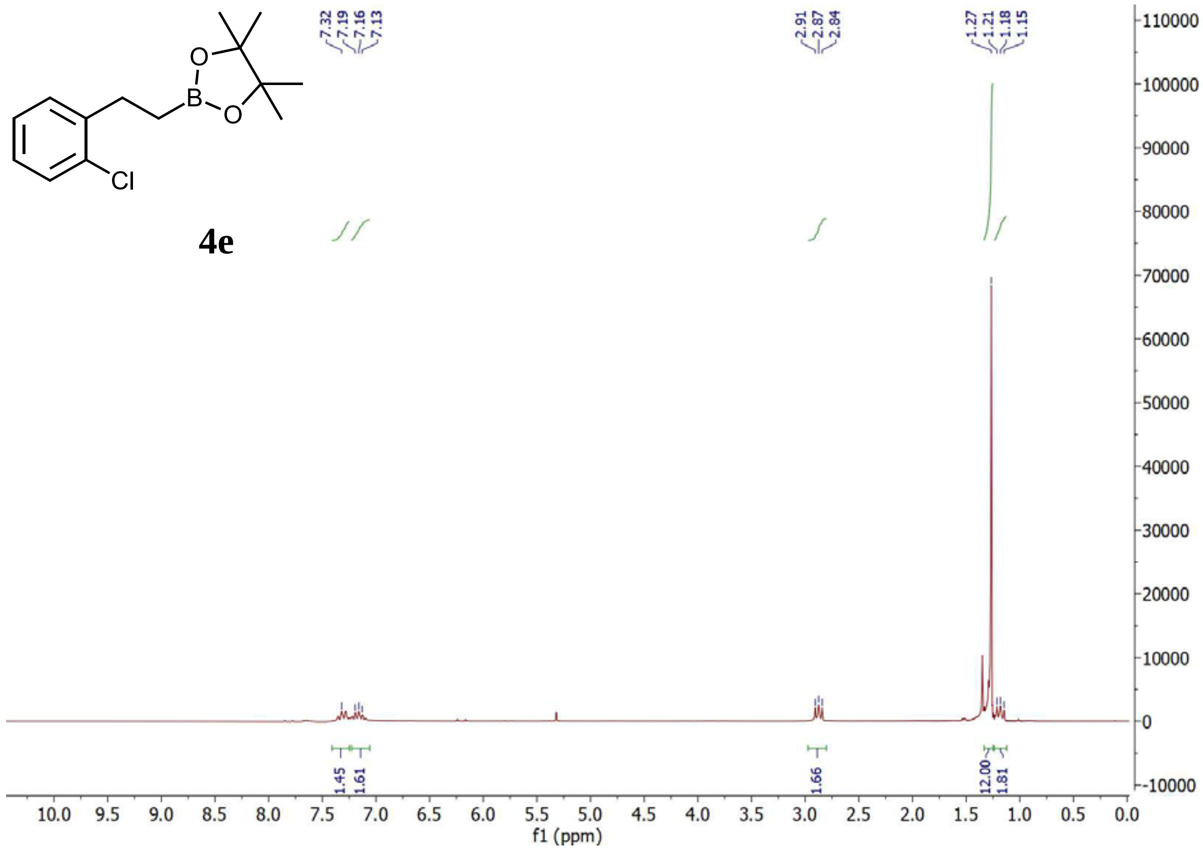
O
B
O
Cl
7.32
7.19
7.16
7.13
2.91
2.87
2.84
1.27
1.21
1.18
1.15
1.45
1.61
1.66
12.00
1.81
f1 (ppm)

4e

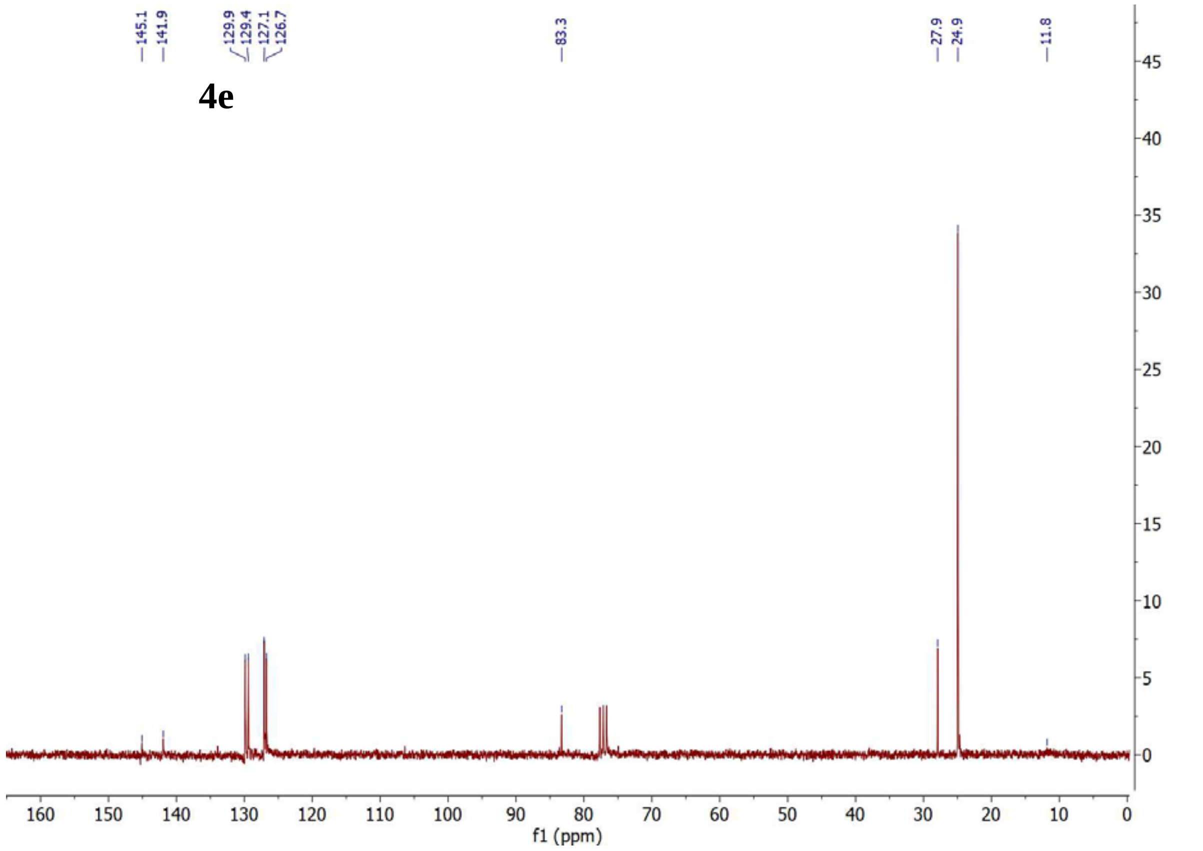
145.1
141.9
129.9
129.4
127.1
126.7
83.3
27.9
24.9
11.8
f1 (ppm)

4e

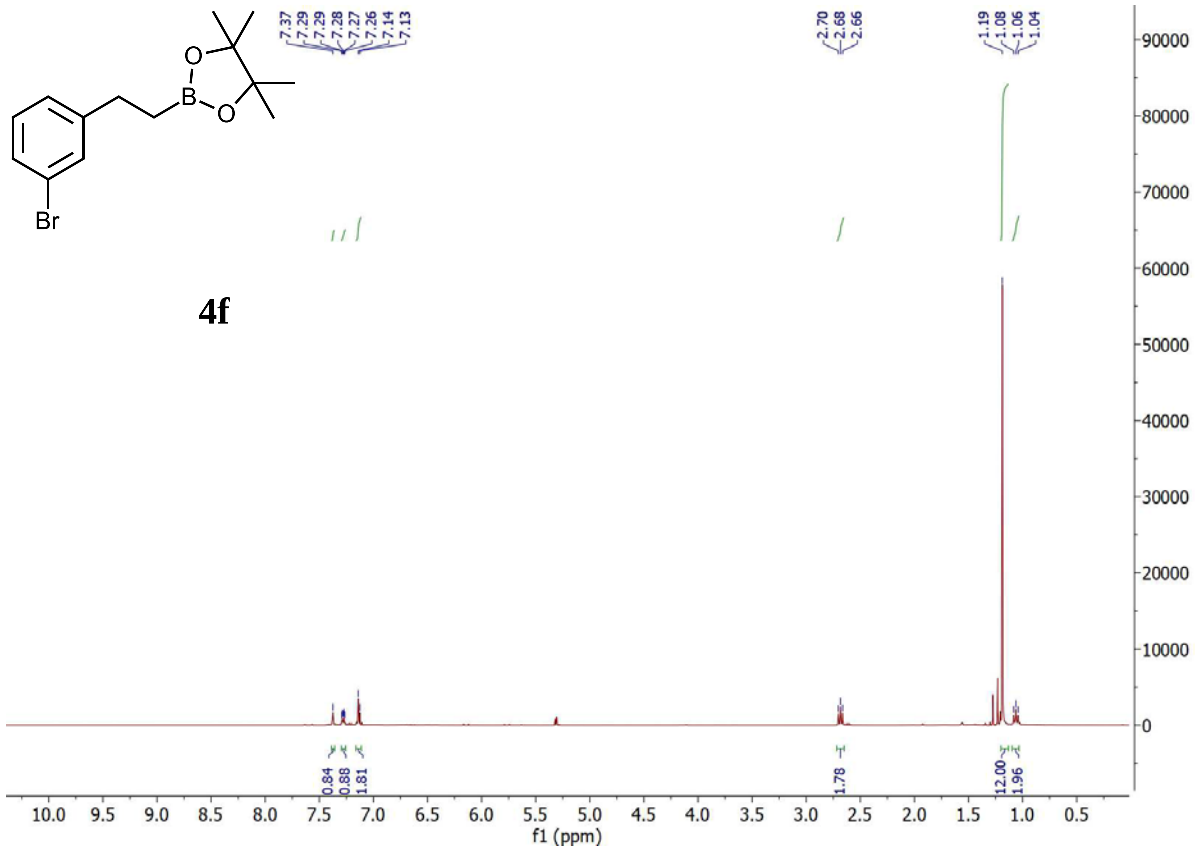
Br
O
B
O
4f
7.37
7.29
7.29
7.28
7.27
7.26
7.14
7.13
2.70
2.68
2.66
1.19
1.08
1.06
1.04
0.84
0.88
1.81
1.78
12.00
1.96
f1 (ppm)

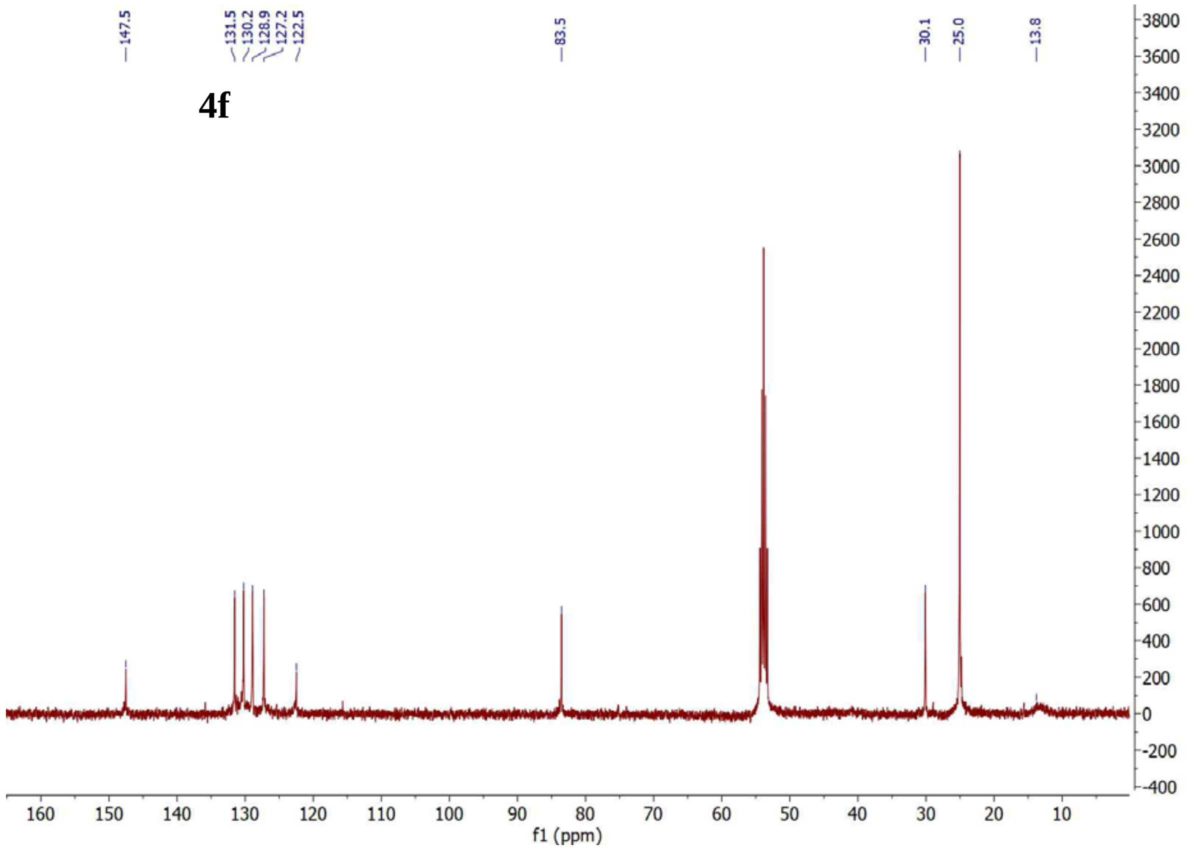
147.5
131.5
130.2
128.9
127.2
122.5
83.5
30.1
25.0
13.8
4f
f1 (ppm)

4g

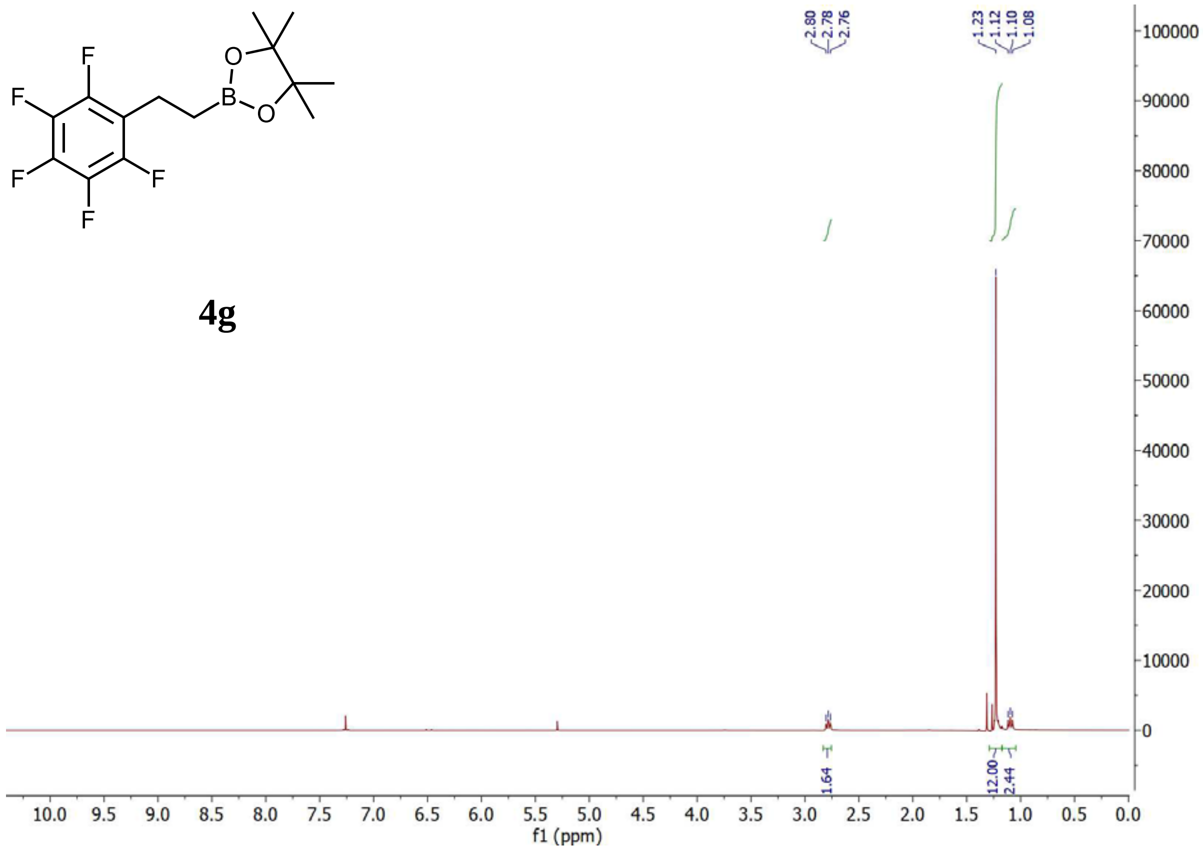

2.80
2.78
2.76
1.23
1.12
1.10
1.08
1.64
12.00
2.44
f1 (ppm)

4g

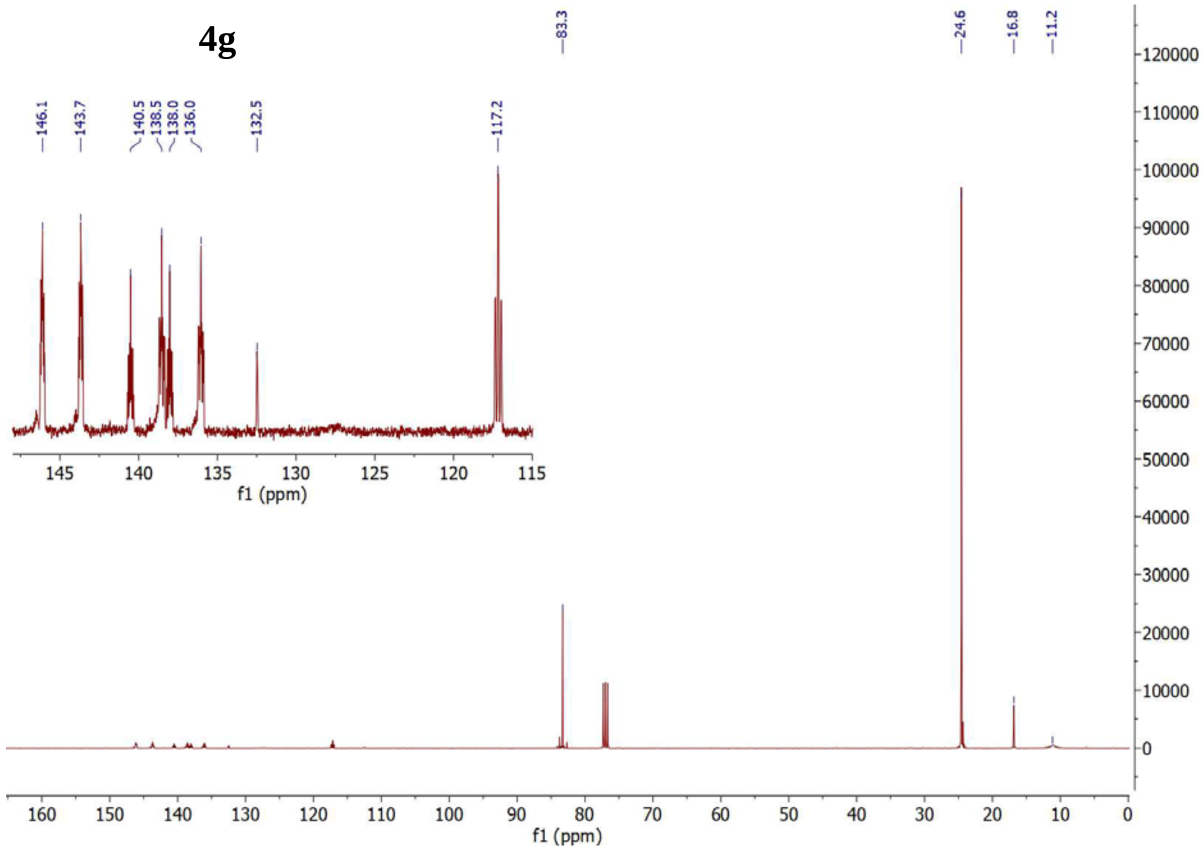

83.3
24.6
16.8
11.2
146.1
143.7
140.5
138.5
138.0
136.0
132.5
117.2
f1 (ppm)
f1 (ppm)

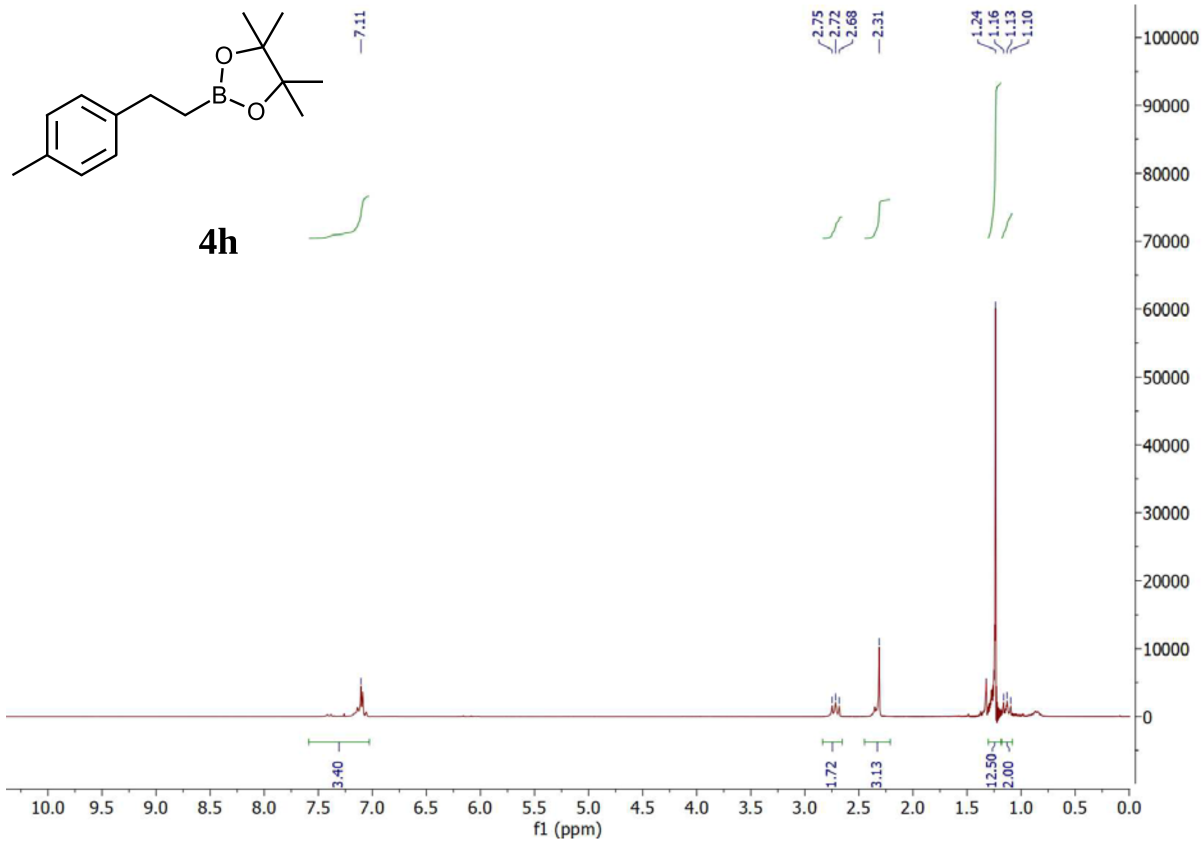
4h
7.11
2.75
2.72
2.68
2.31
1.24
1.16
1.13
1.10
3.40
1.72
3.13
12.50
2.00
f1 (ppm)

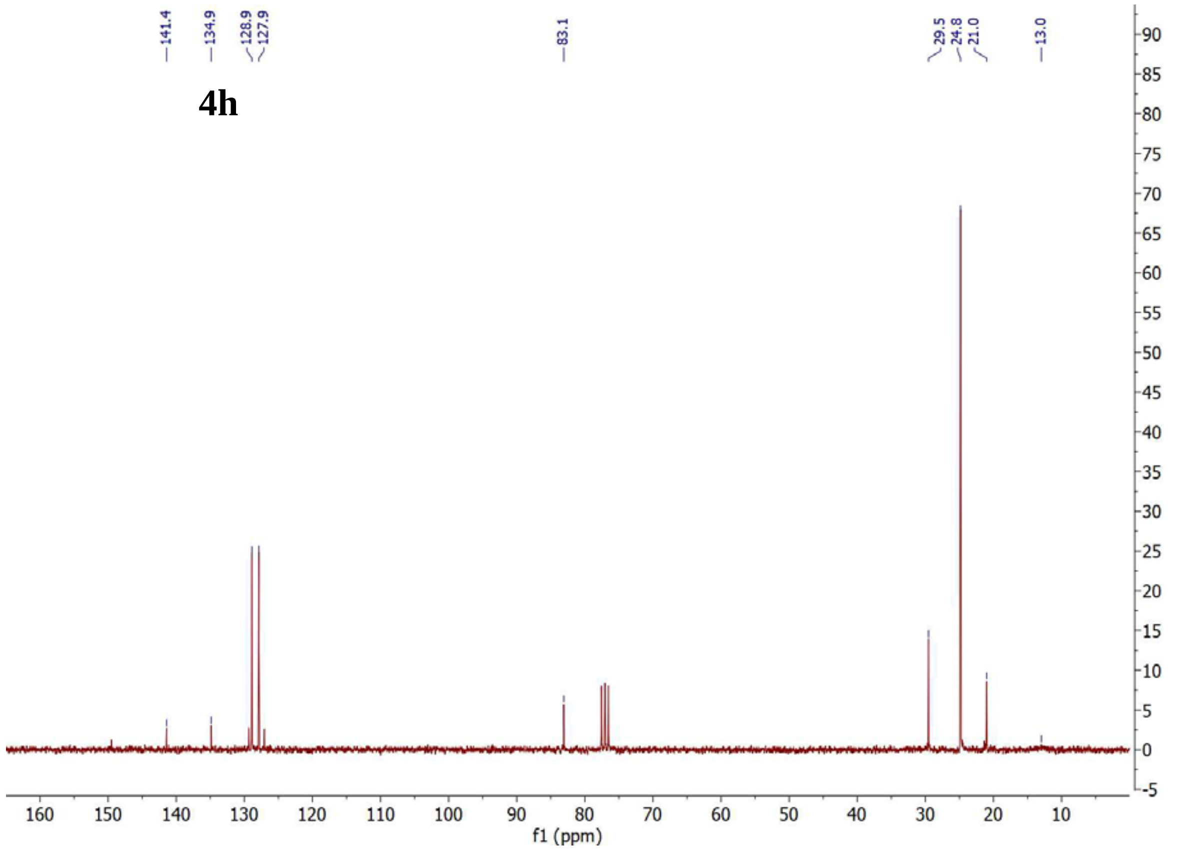
4h
141.4
134.9
128.9
127.9
83.1
29.5
24.8
21.0
13.0
f1 (ppm)

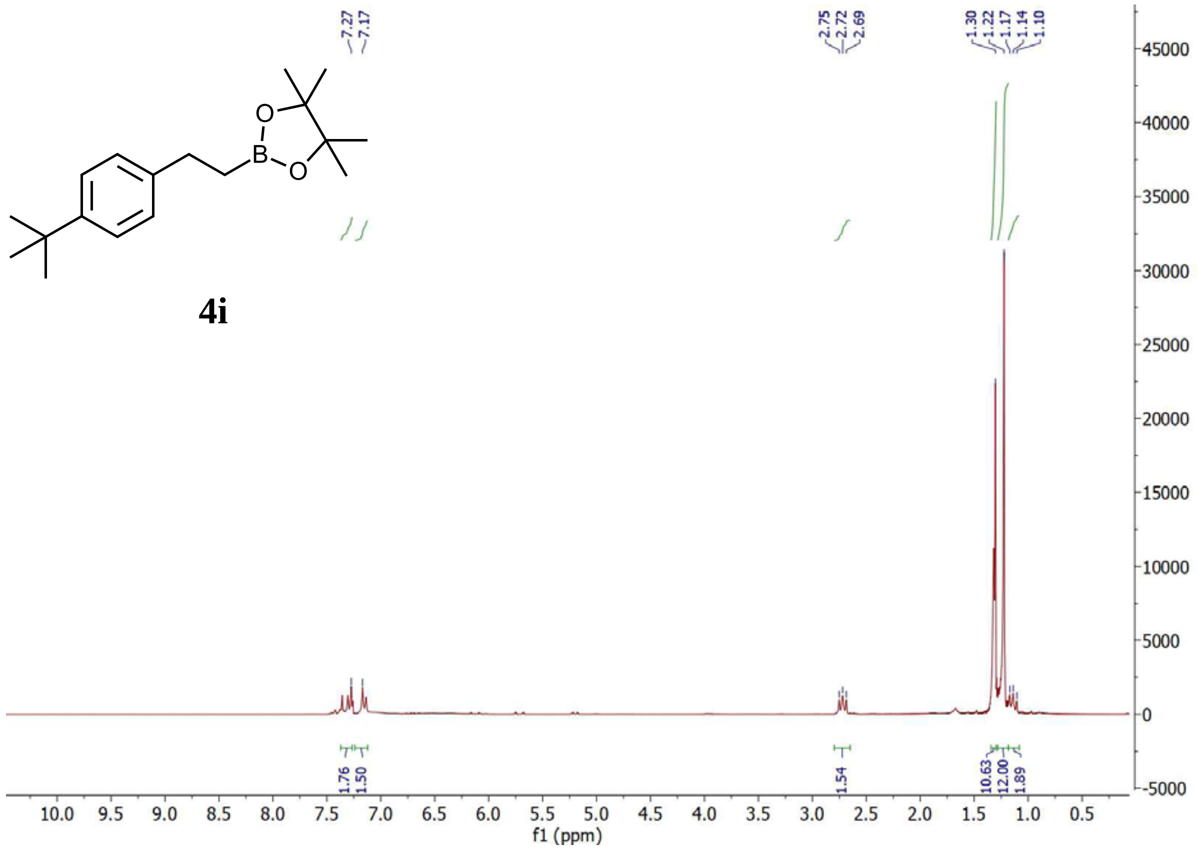
4i
7.27
7.17
2.75
2.72
2.69
1.30
1.22
1.17
1.14
1.10
1.76
1.50
1.54
10.63
12.00
1.89
f1 (ppm)

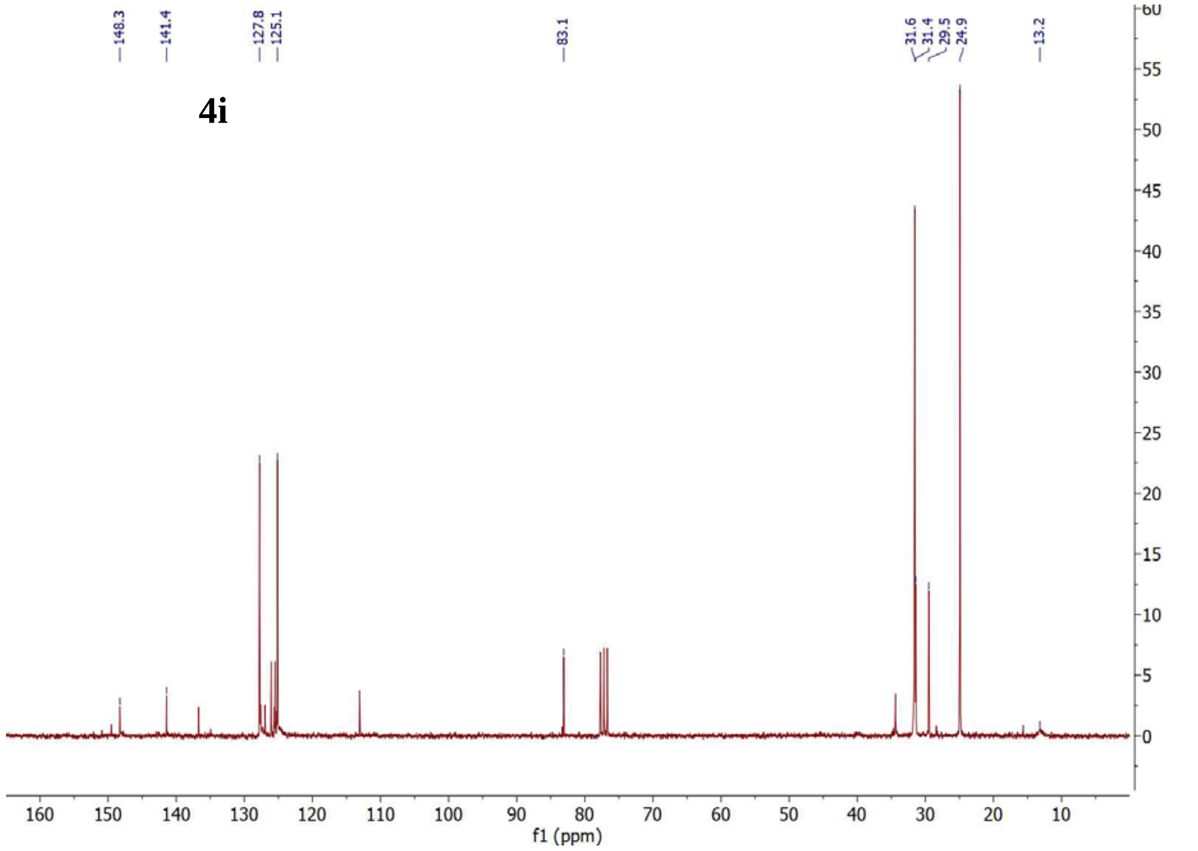
4i
148.3
141.4
127.8
125.1
83.1
31.6
31.4
29.5
24.9
13.2
f1 (ppm)

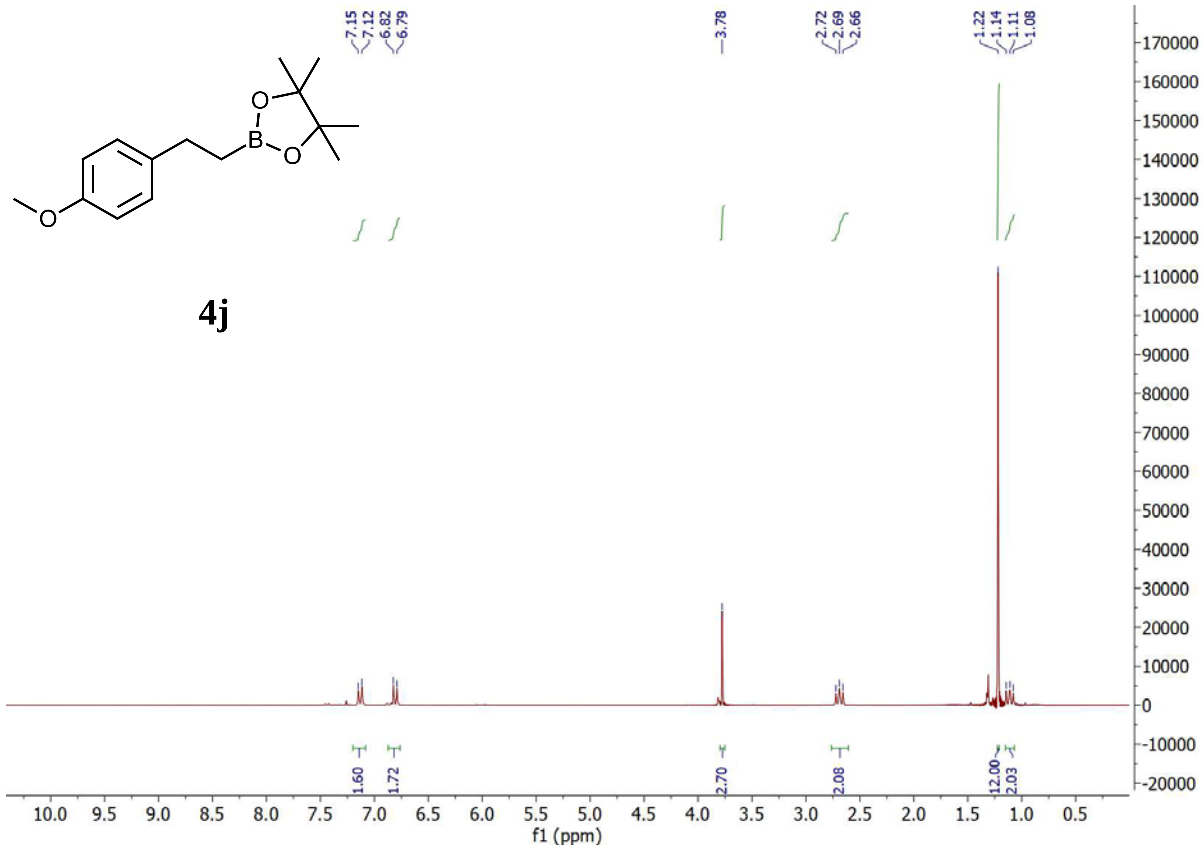
7.15
7.12
6.82
6.79
3.78
2.72
2.69
2.66
1.22
1.14
1.11
1.08
1.60
1.72
2.70
2.08
12.00
2.03
f1 (ppm)

4j

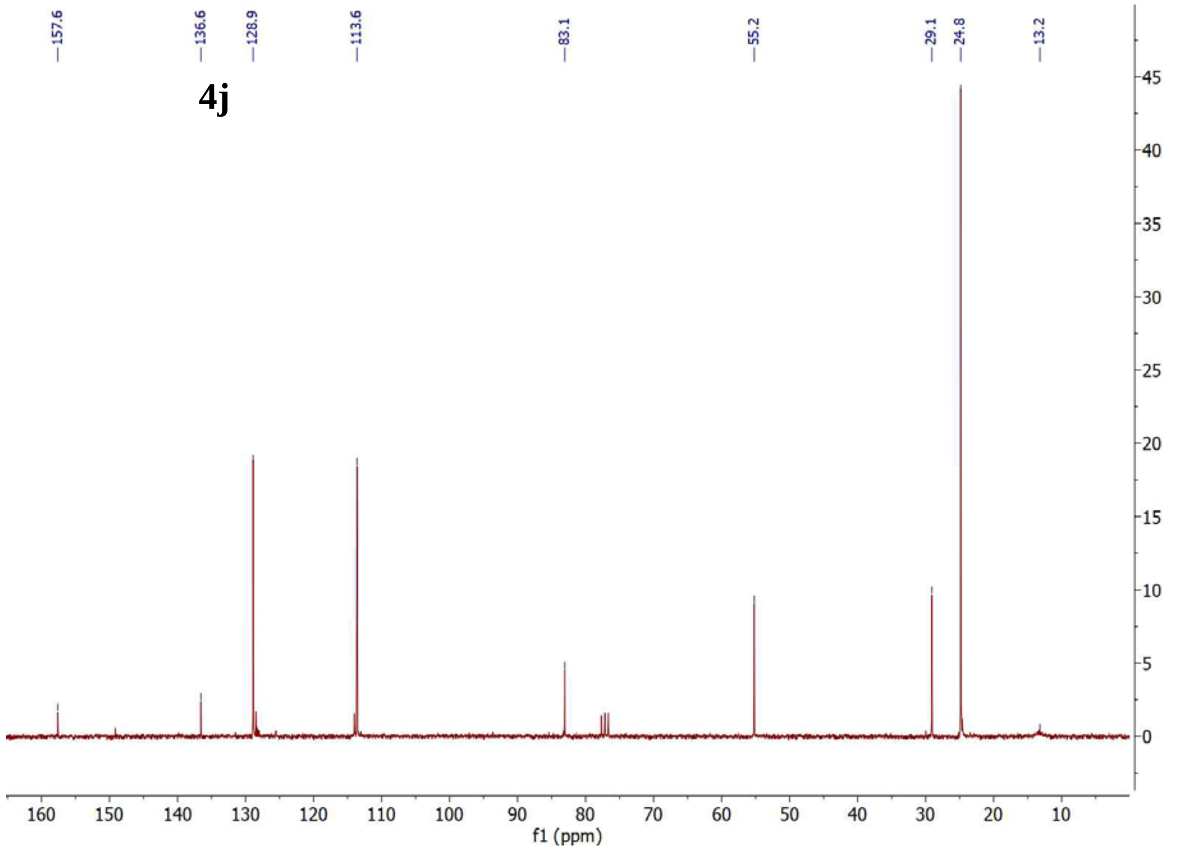
157.6
136.6
128.9
113.6
83.1
55.2
29.1
24.8
13.2
f1 (ppm)

4j

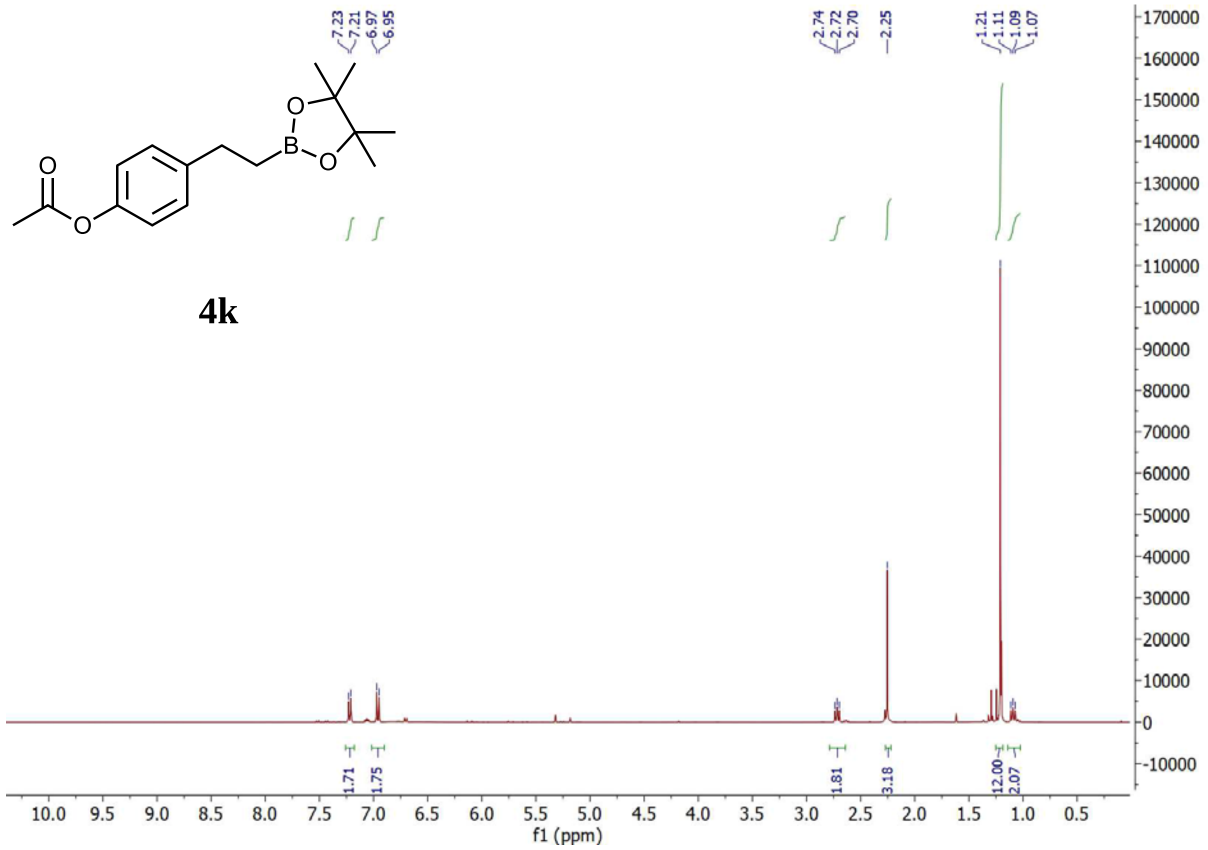
4k
7.23
7.21
6.97
6.95
2.74
2.72
2.70
2.25
1.21
1.11
1.09
1.07
1.71
1.75
1.81
3.18
12.00
2.07
f1 (ppm)

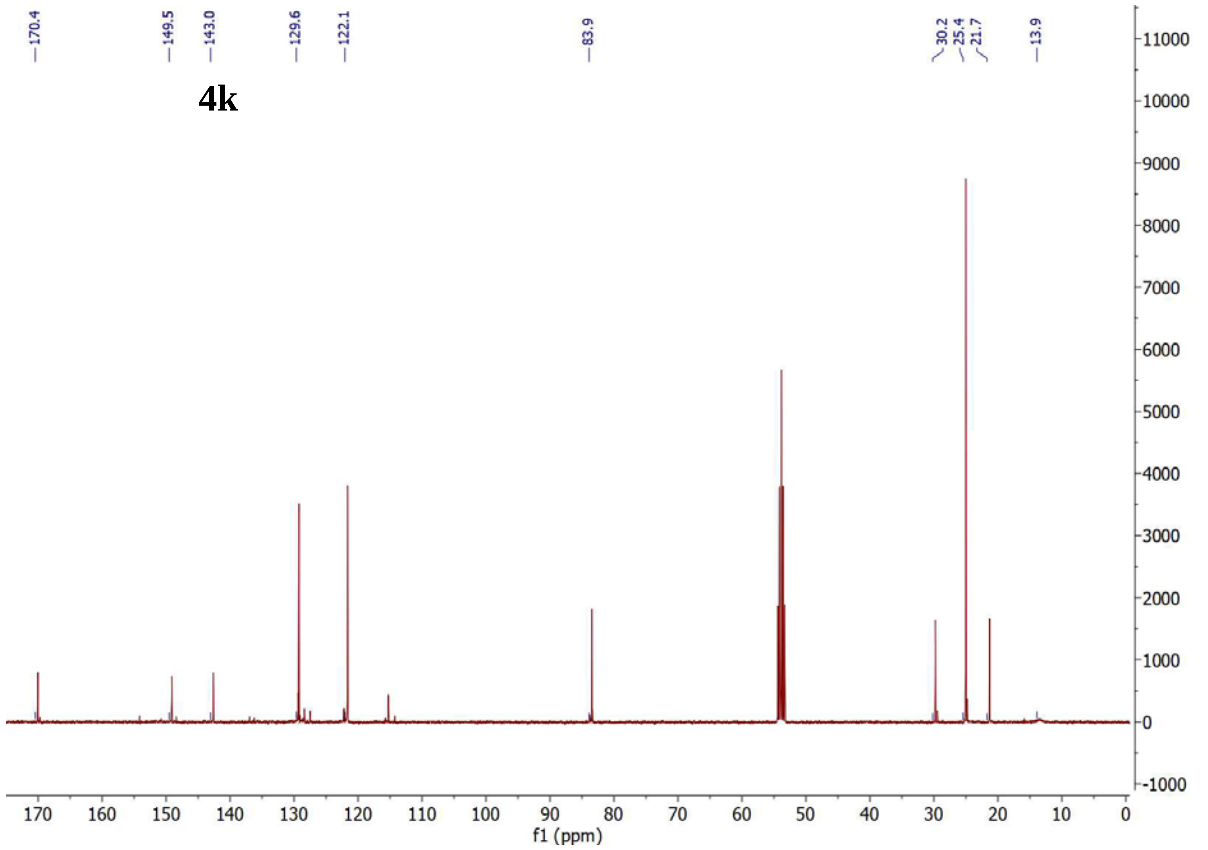
4k
170.4
149.5
143.0
129.6
122.1
83.9
30.2
25.4
21.7
13.9
f1 (ppm)

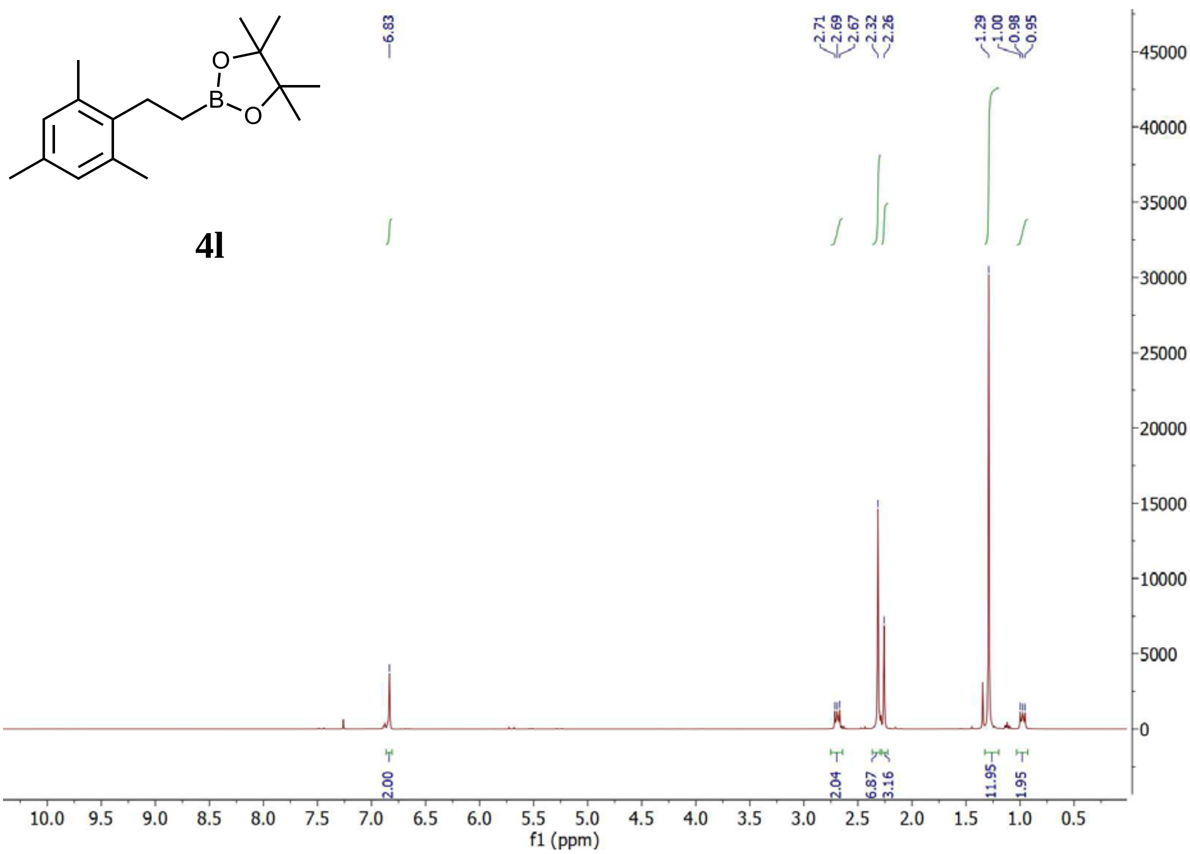

O
B
O
6.83
2.71
2.69
2.67
2.32
2.26
1.29
1.00
0.98
0.95
2.00
2.04
6.87
3.16
11.95
1.95
f1 (ppm)

4l

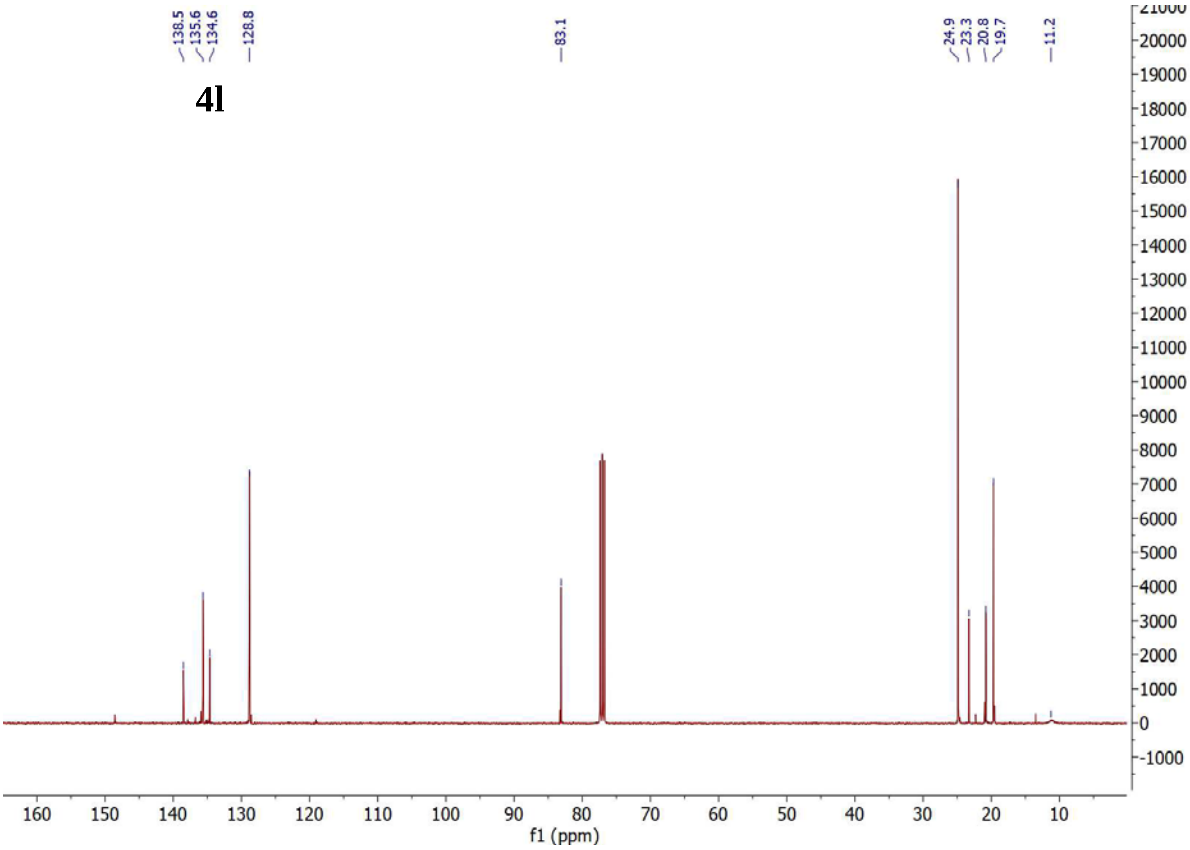

138.5
135.6
134.6
128.8
83.1
24.9
23.3
20.8
19.7
11.2
f1 (ppm)

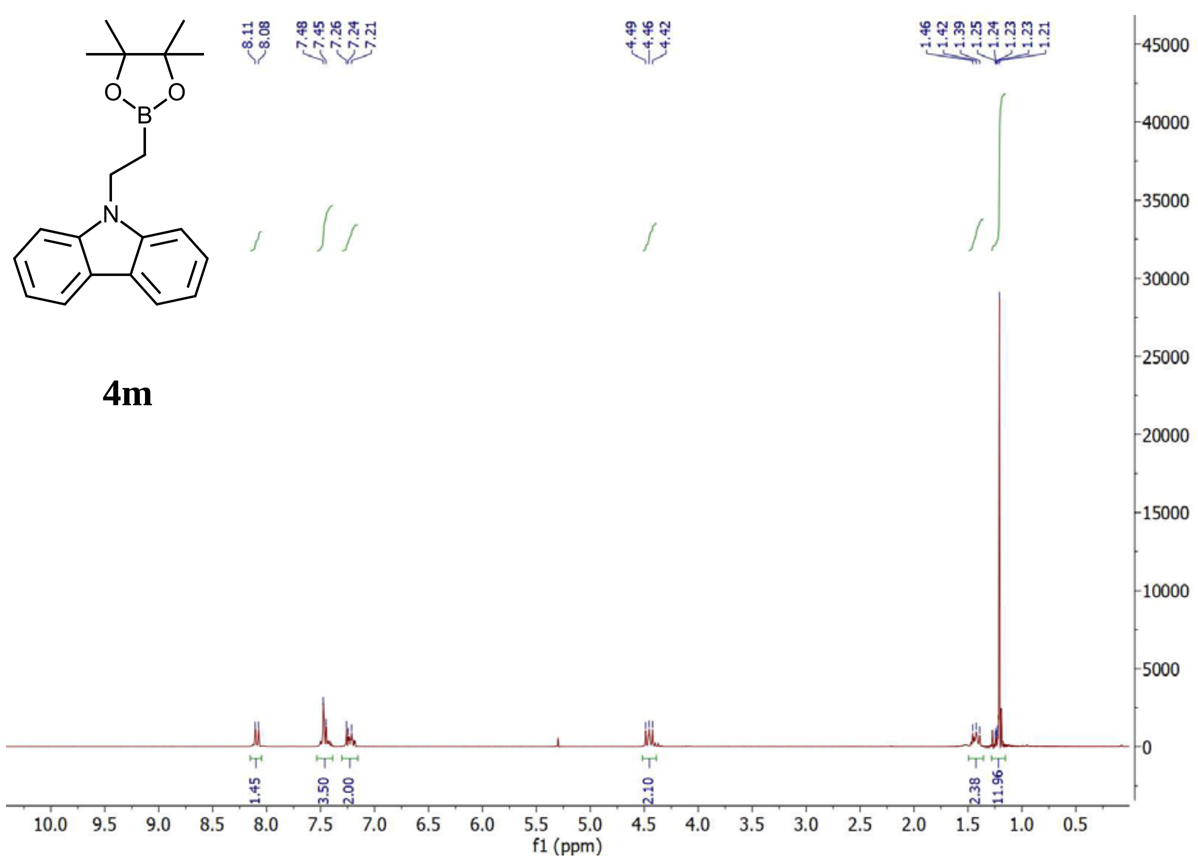
8.11
8.08
7.48
7.45
7.26
7.24
7.21
4.49
4.46
4.42
1.46
1.42
1.39
1.25
1.24
1.23
1.23
1.21
1.45
3.50
2.00
2.10
2.38
11.96
f1 (ppm)

4m

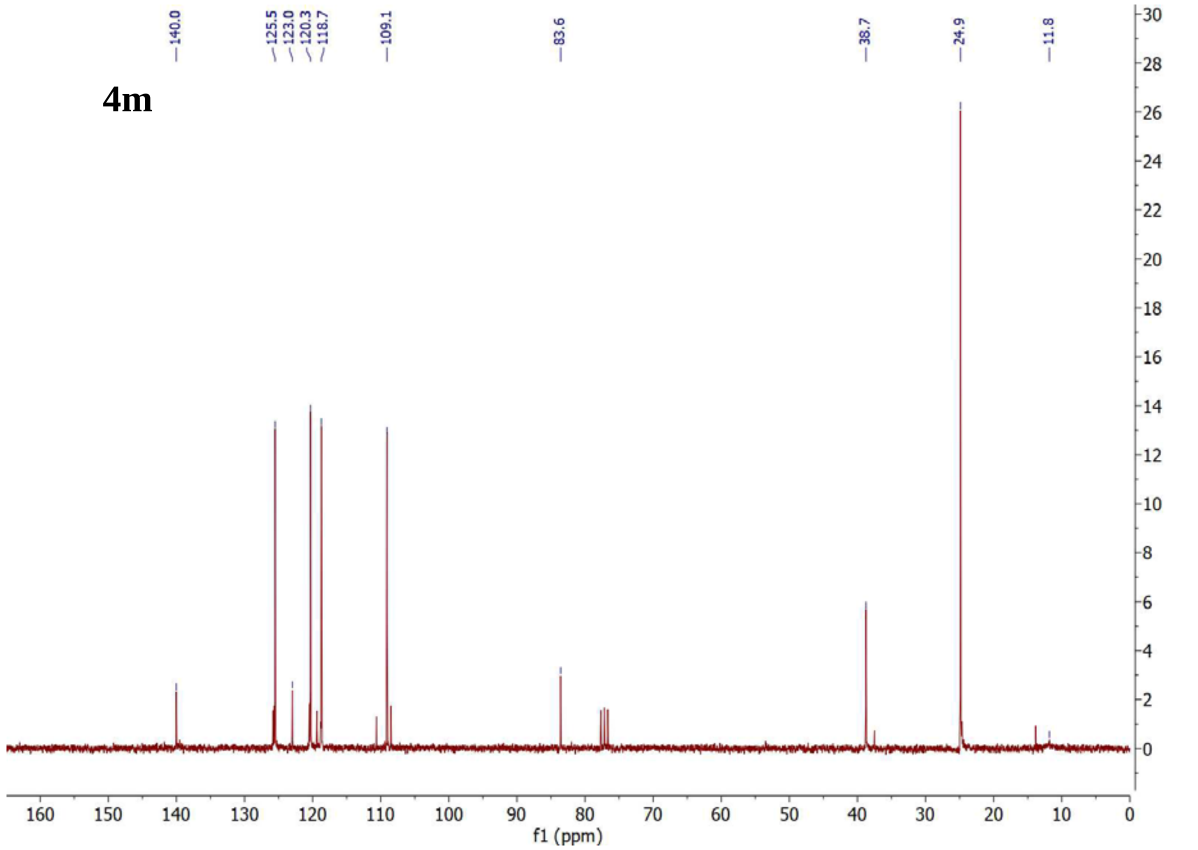
140.0
125.5
123.0
120.3
118.7
109.1
83.6
38.7
24.9
11.8
f1 (ppm)

4m

4n

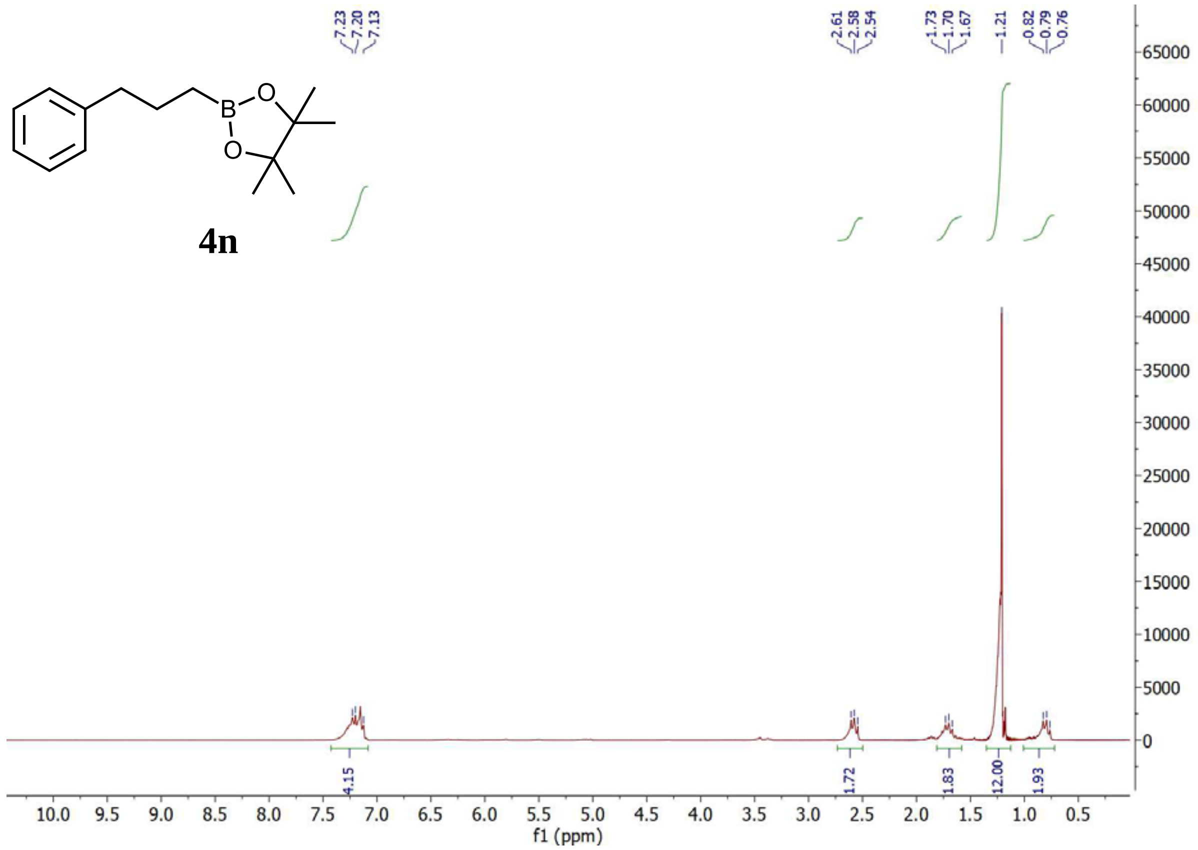
7.23
7.20
7.13
2.61
2.58
2.54
1.73
1.70
1.67
1.21
0.82
0.79
0.76
4.15
1.72
1.83
12.00
1.93
f1 (ppm)

4n

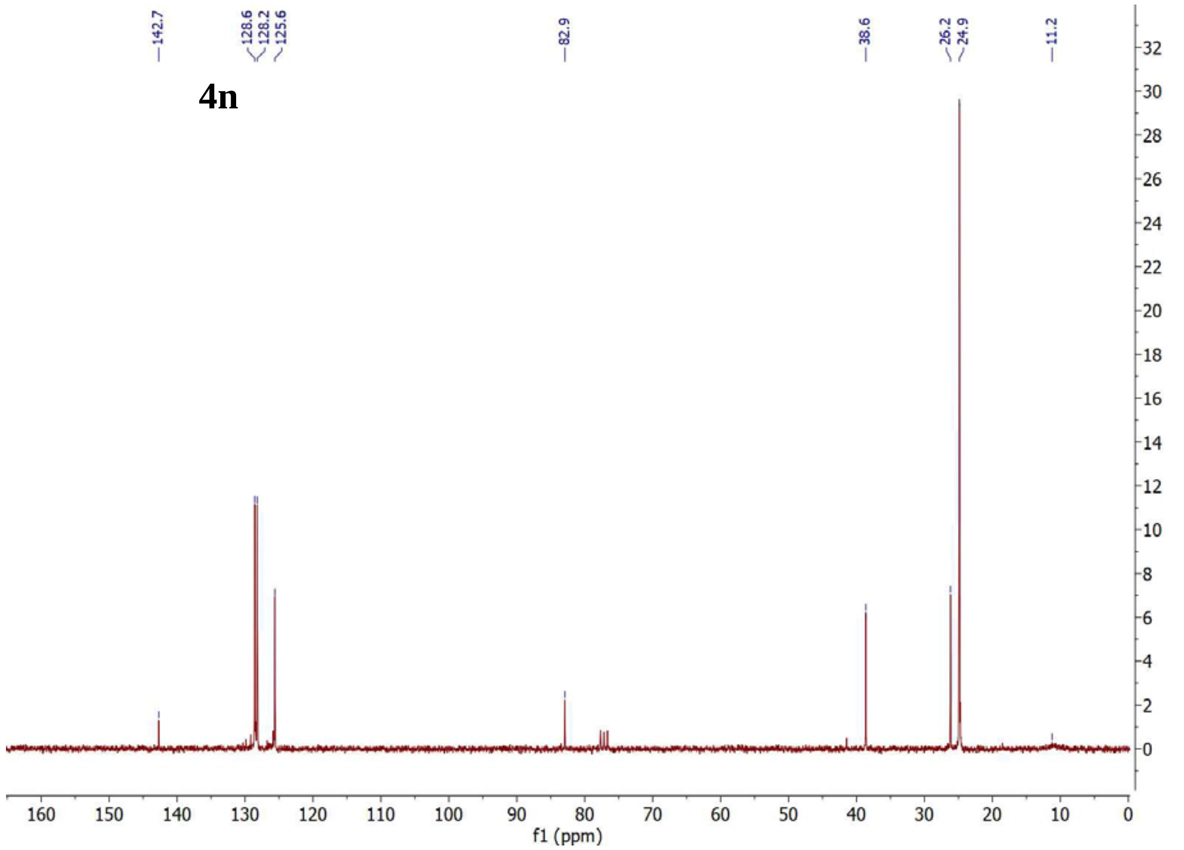
142.7
128.6
128.2
125.6
82.9
38.6
26.2
24.9
11.2
f1 (ppm)

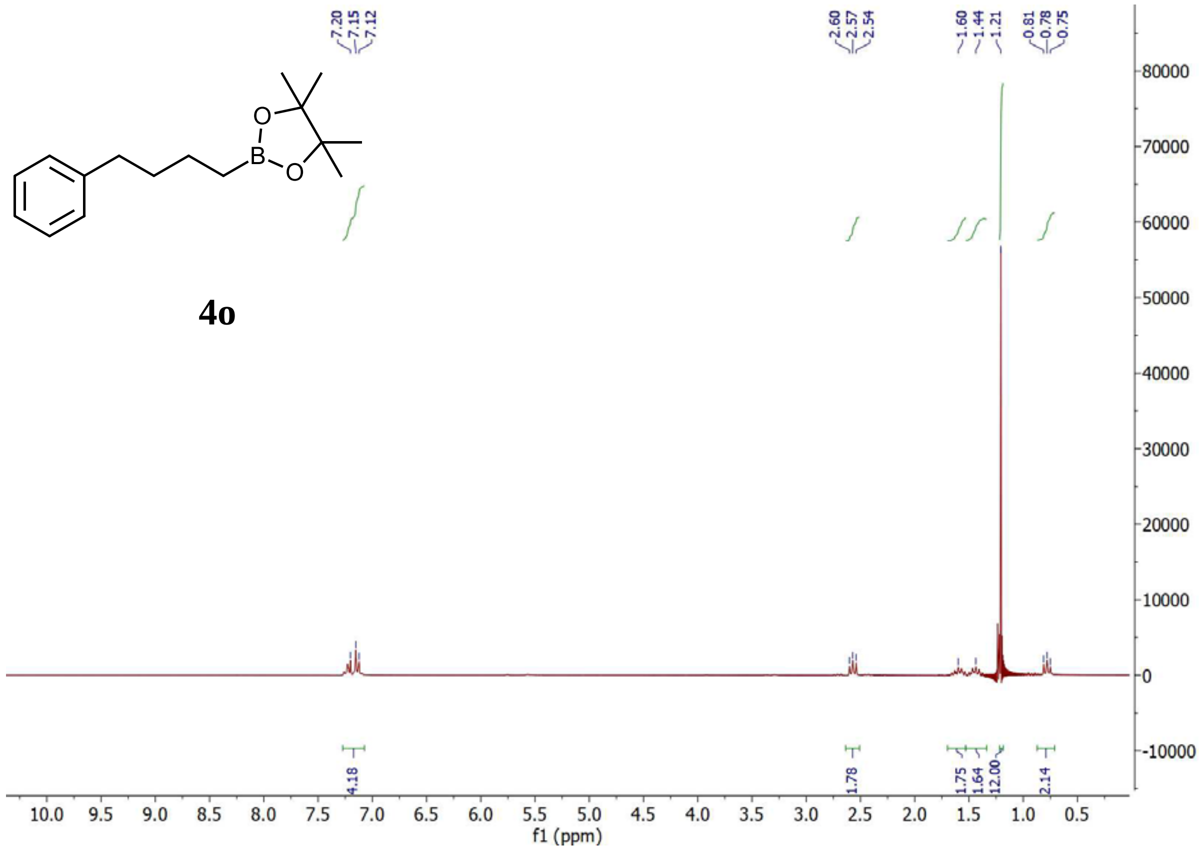
7.20
7.15
7.12
2.60
2.57
2.54
1.60
1.44
1.21
0.81
0.78
0.75
O
B
O
4o
4.18
1.78
1.75
1.64
12.00
2.14
f1 (ppm)

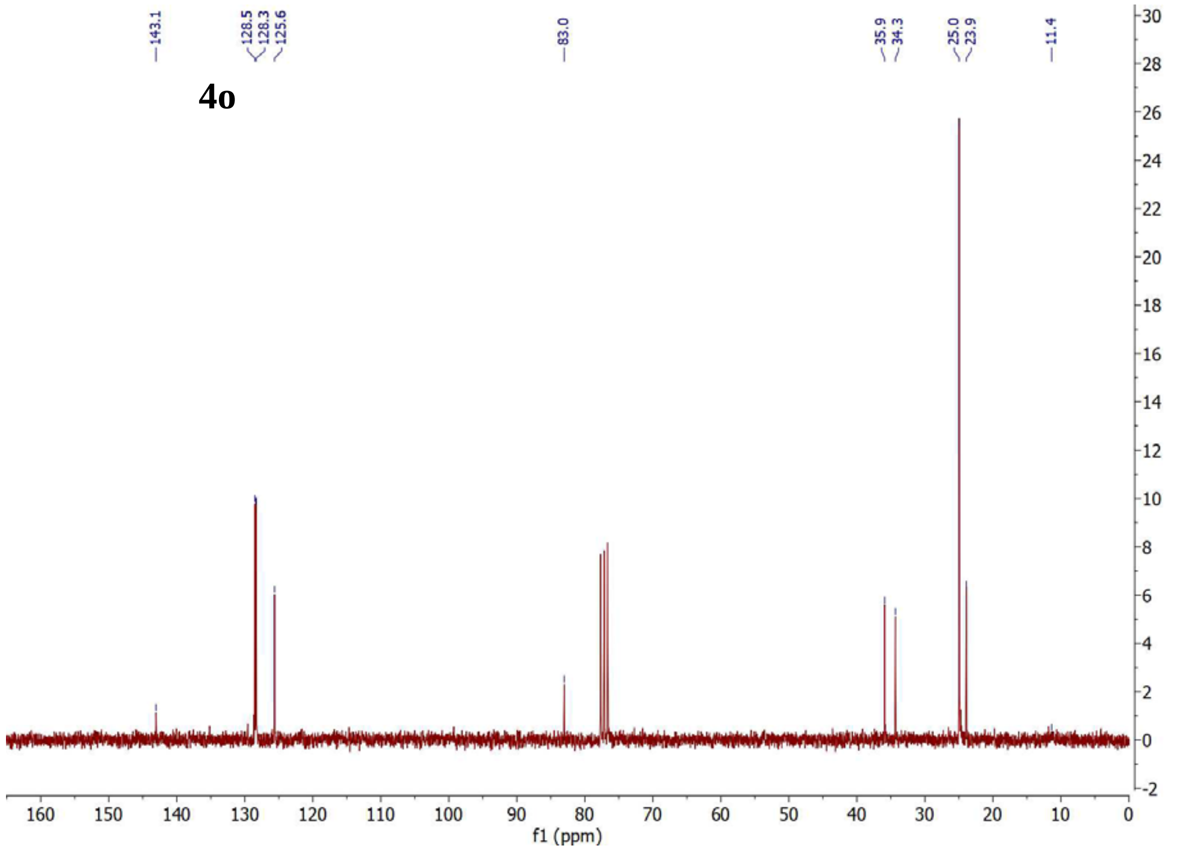
143.1
128.5
128.3
125.6
83.0
35.9
34.3
25.0
23.9
11.4
4o
f1 (ppm)

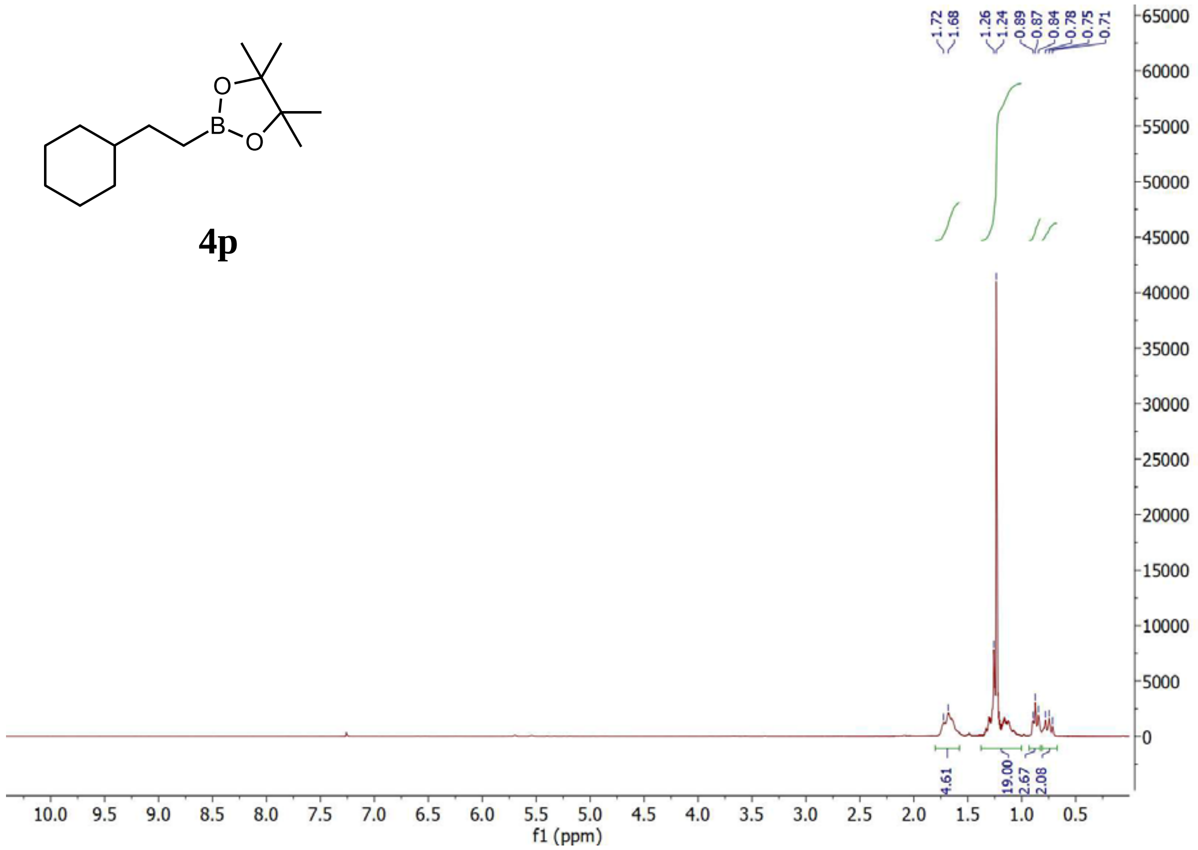
O
B
O
1.72
1.68
1.26
1.24
0.89
0.87
0.84
0.78
0.75
0.71
4.61
19.00
2.67
2.08
f1 (ppm)

4p

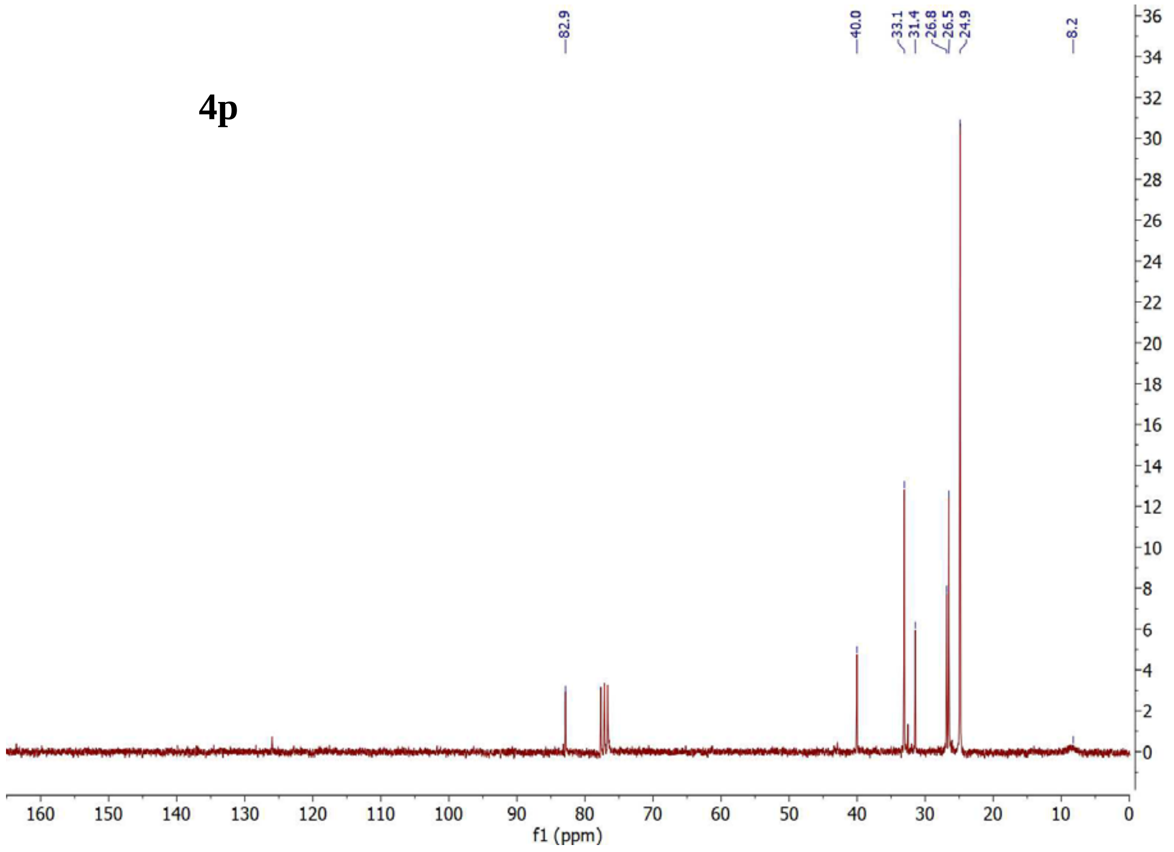
82.9
40.0
33.1
31.4
26.8
26.5
24.9
8.2
f1 (ppm)

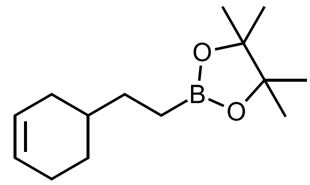

4q

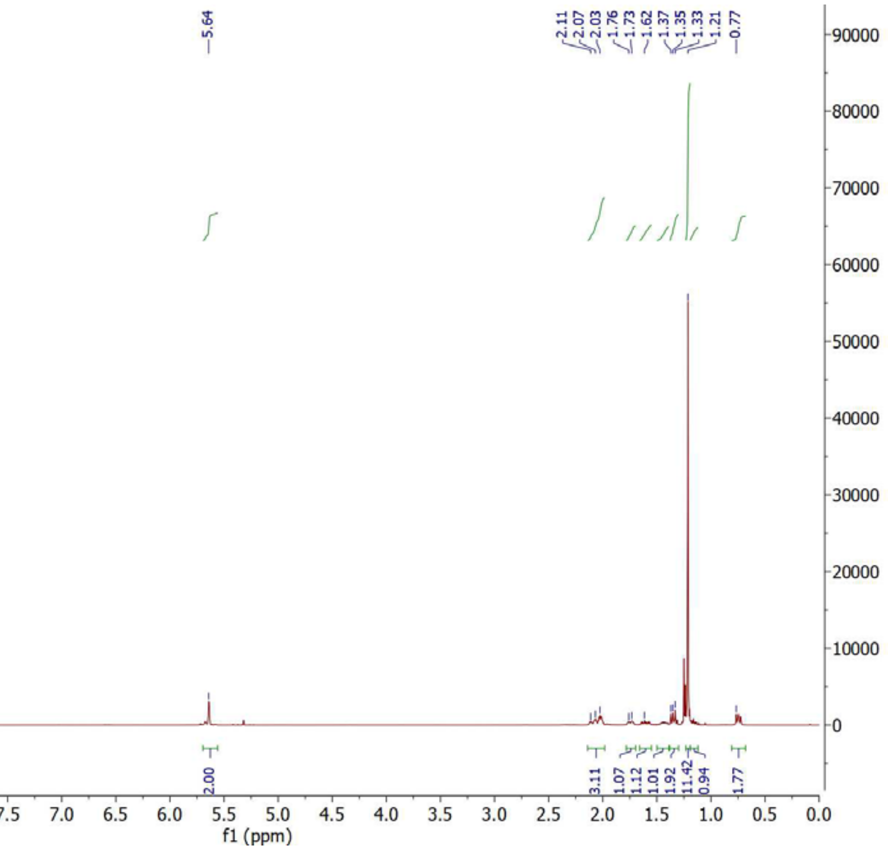

5.64
2.11
2.07
2.03
1.76
1.73
1.62
1.37
1.35
1.33
1.21
0.77
2.00
3.11
1.07
1.12
1.01
1.92
11.42
0.94
1.77
f1 (ppm)

4q

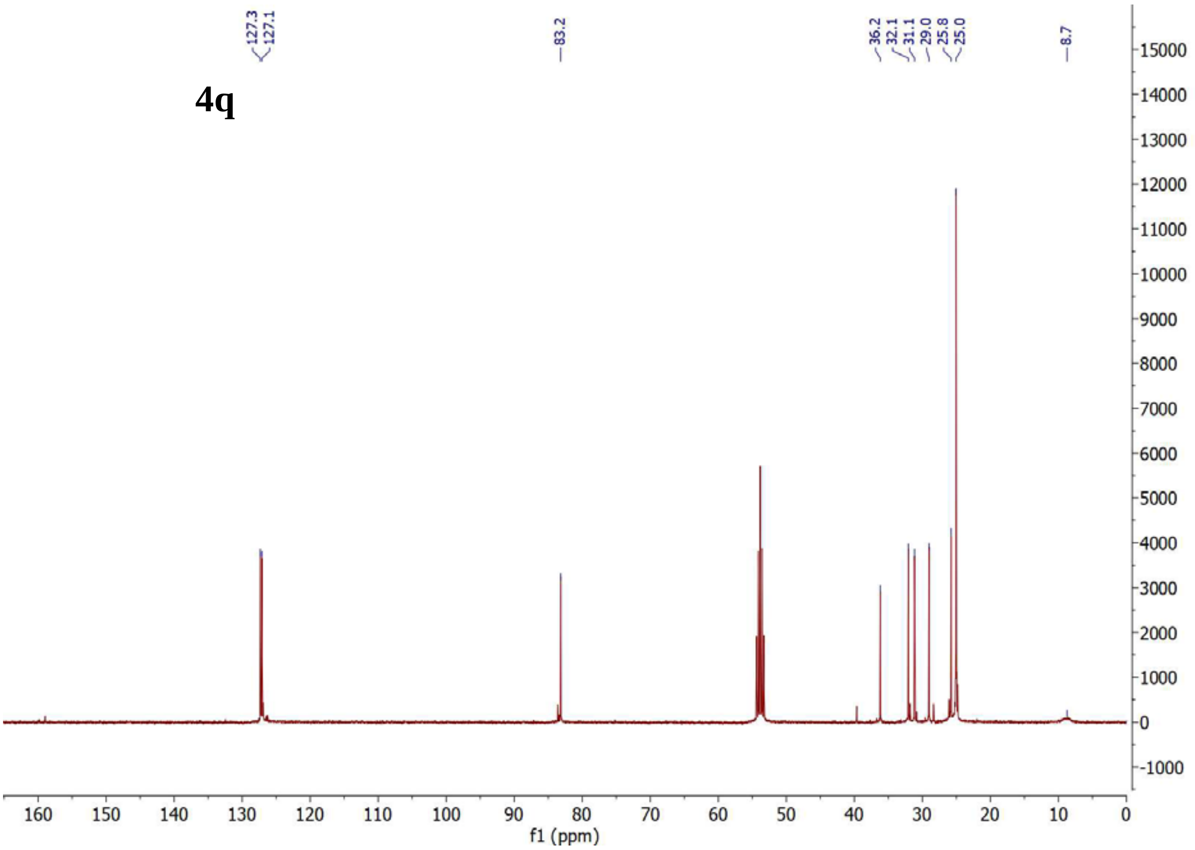

127.3
127.1
83.2
36.2
32.1
31.1
29.0
25.8
25.0
8.7
f1 (ppm)

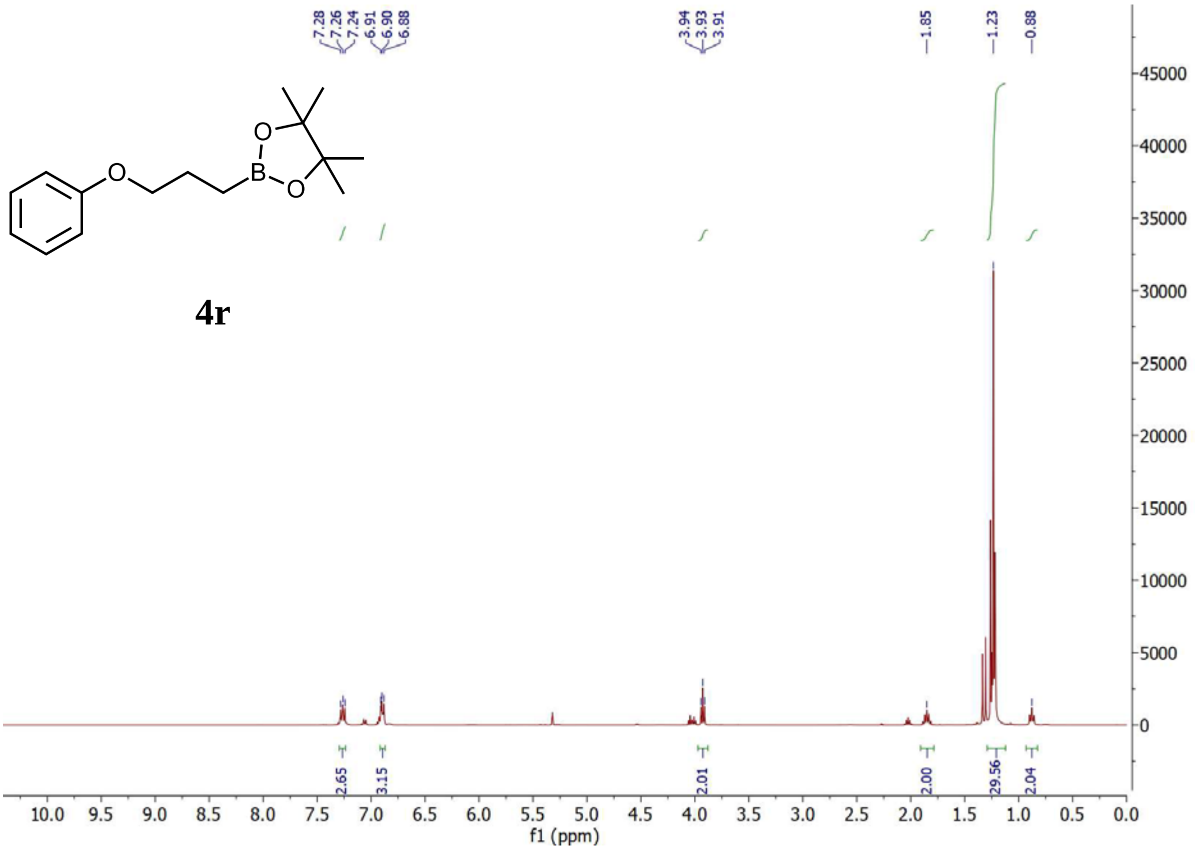

7.28
7.26
7.24
6.91
6.90
6.88
3.94
3.93
3.91
1.85
1.23
0.88
O
B
O
O
2.65
3.15
2.01
2.00
29.56
2.04
f1 (ppm)

4r

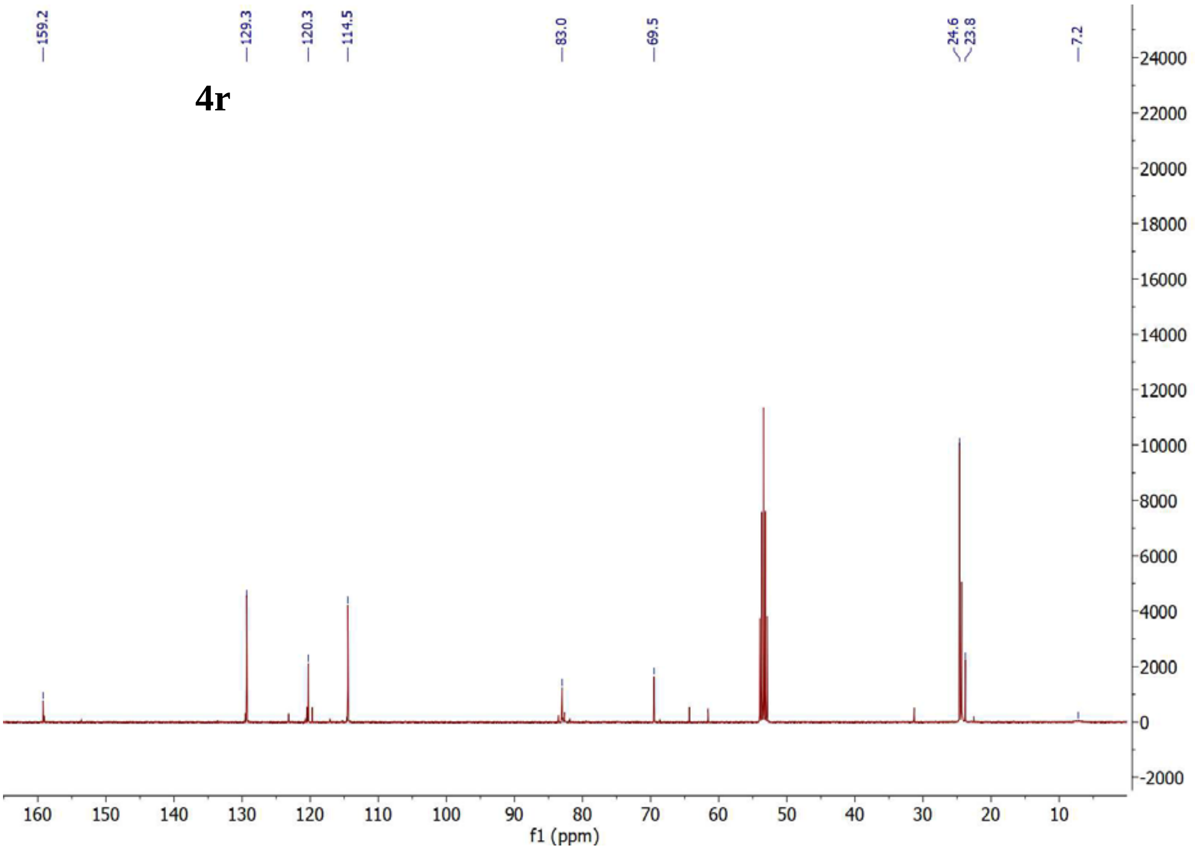

159.2
129.3
120.3
114.5
83.0
69.5
24.6
23.8
7.2
f1 (ppm)

4r

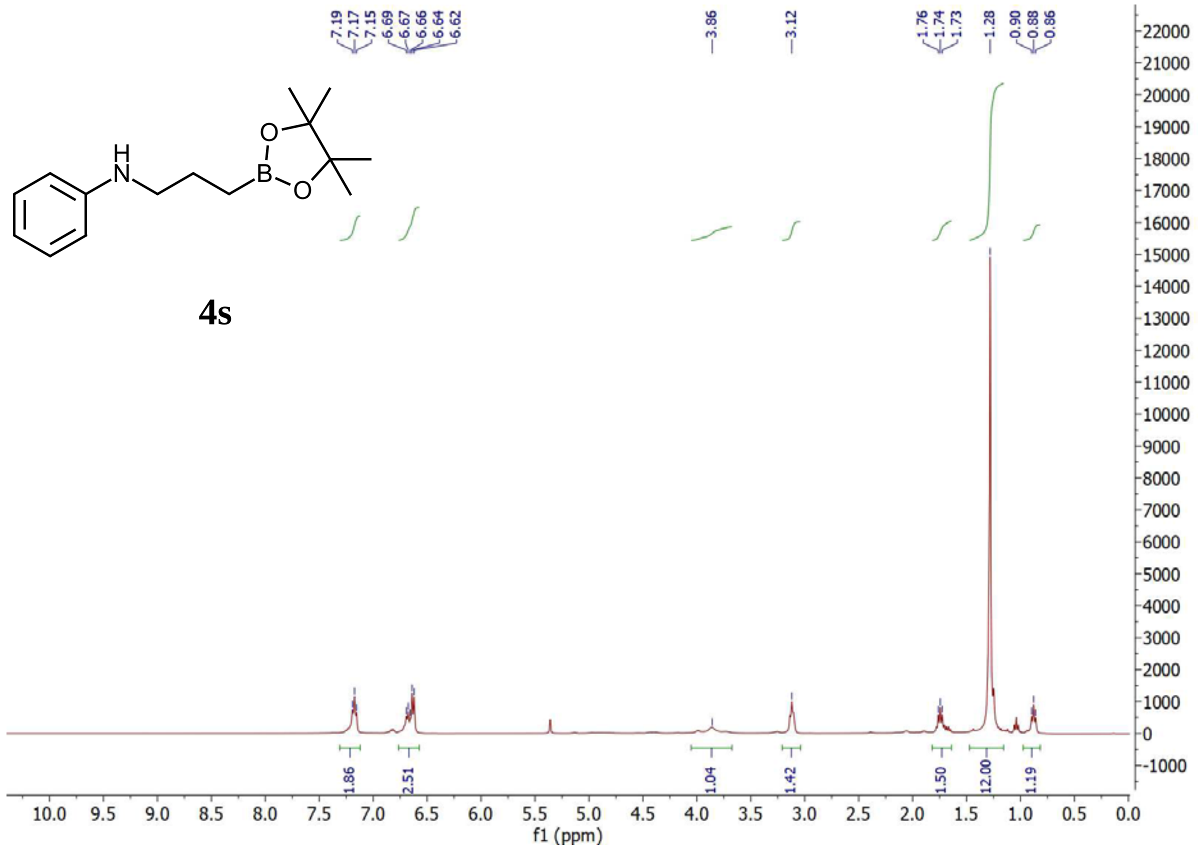
4s
7.19
7.17
7.15
6.69
6.67
6.66
6.64
6.62
3.86
3.12
1.76
1.74
1.73
1.28
0.90
0.88
0.86
1.86
2.51
1.04
1.42
1.50
12.00
1.19
f1 (ppm)

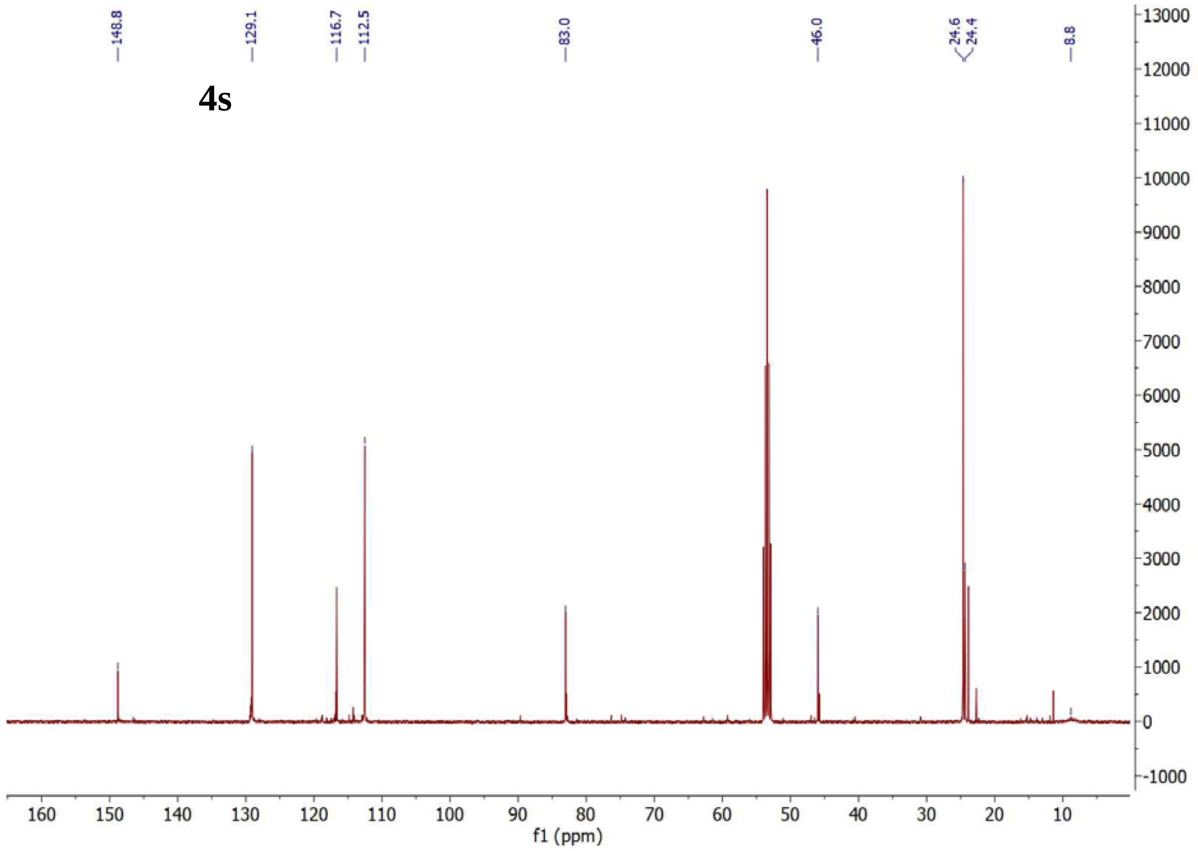
4s
148.8
129.1
116.7
112.5
83.0
46.0
24.6
24.4
8.8
f1 (ppm)

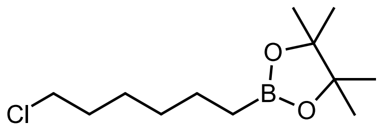
Cl
B
O
O

4t

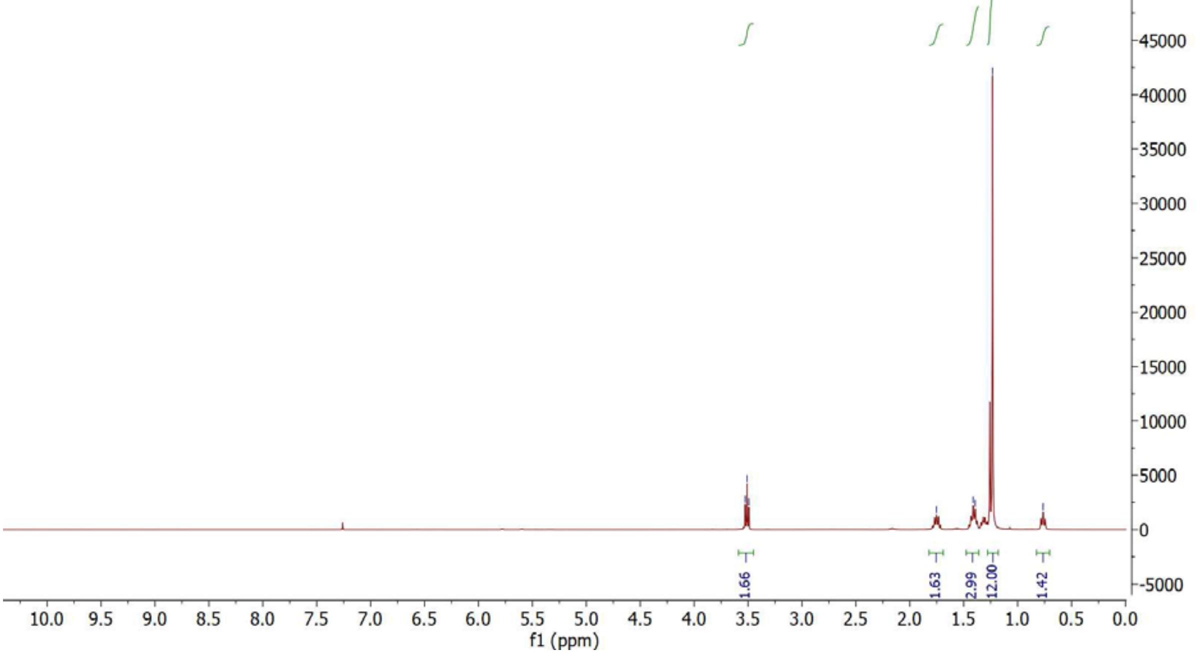
3.53
3.51
3.49
1.75
1.41
1.39
1.23
0.76
1.66
1.63
2.99
12.00
1.42
f1 (ppm)

4t

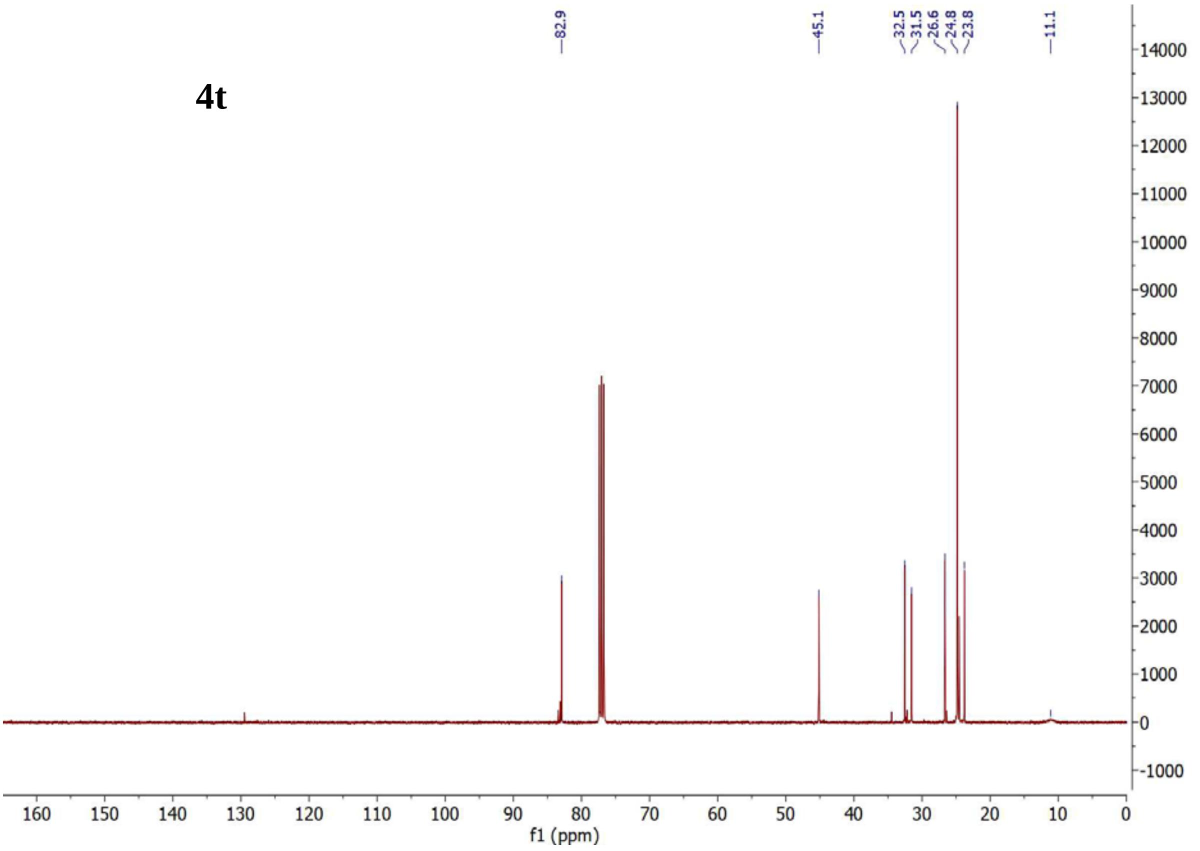
82.9
45.1
32.5
31.5
26.6
24.8
23.8
11.1
f1 (ppm)

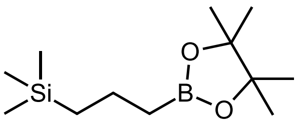

4u

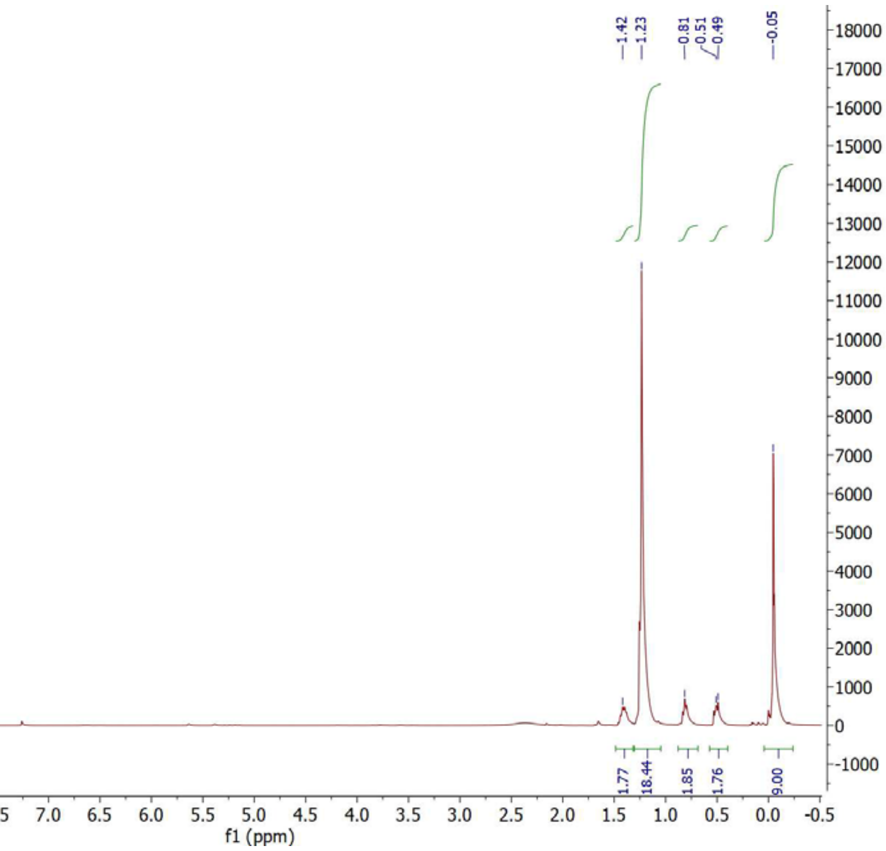

1.42
1.23
0.81
0.51
0.49
-0.05
1.77
18.44
1.85
1.76
9.00
f1 (ppm)

4u

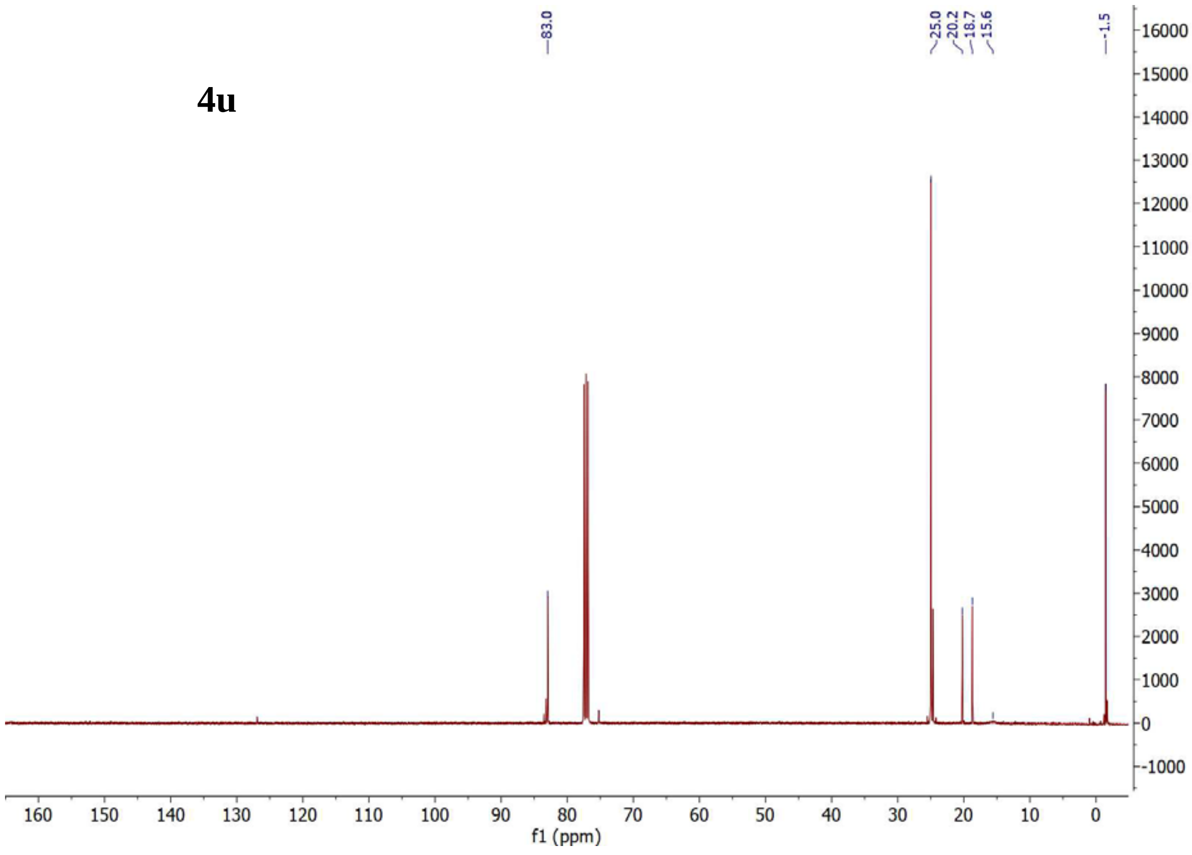

83.0
25.0
20.2
18.7
15.6
-1.5
f1 (ppm)

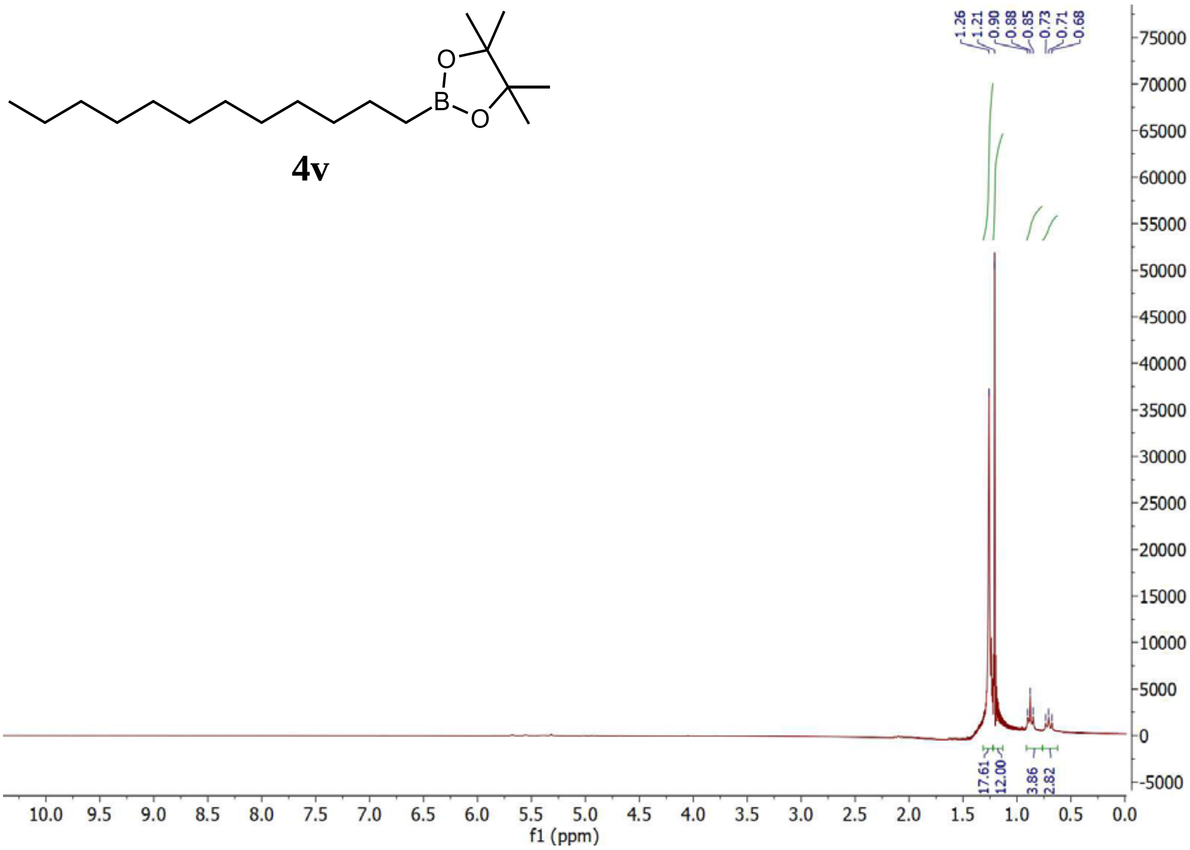
1.26
1.21
0.90
0.88
0.85
0.73
0.71
0.68
17.61
12.00
3.86
2.82
f1 (ppm)

4v

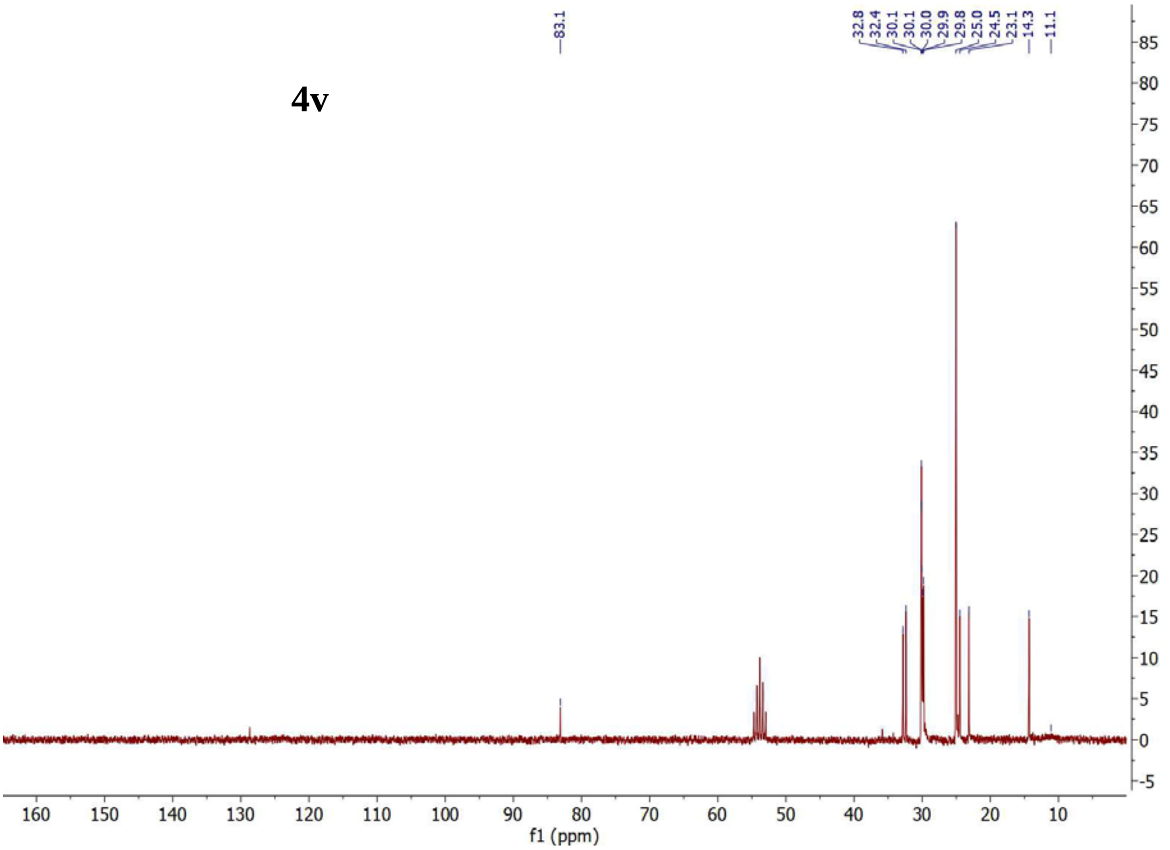
83.1
32.8
32.4
30.1
30.1
30.0
29.9
29.8
25.0
24.5
23.1
14.3
11.1
f1 (ppm)

4v

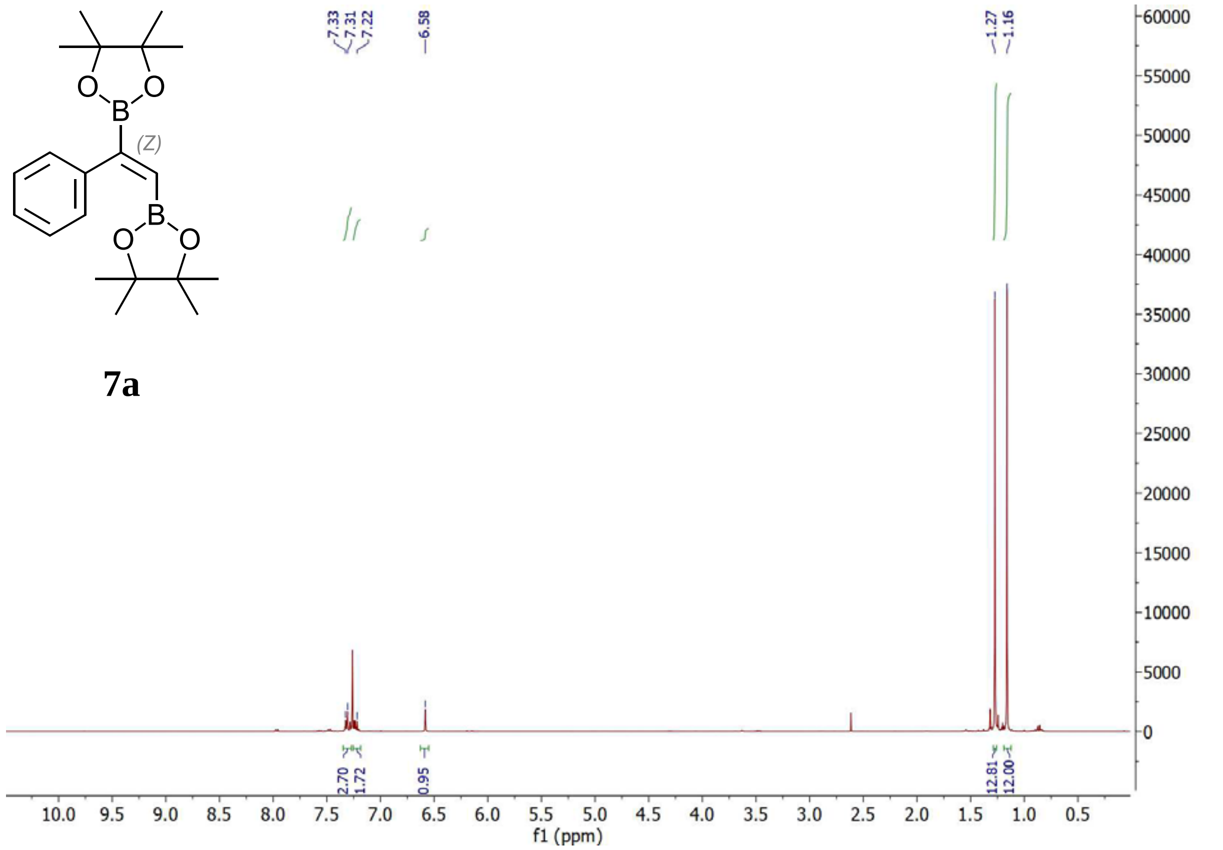

(Z)
7.33
7.31
7.22
6.58
1.27
1.16
2.70
1.72
0.95
12.81
12.00
f1 (ppm)

7a

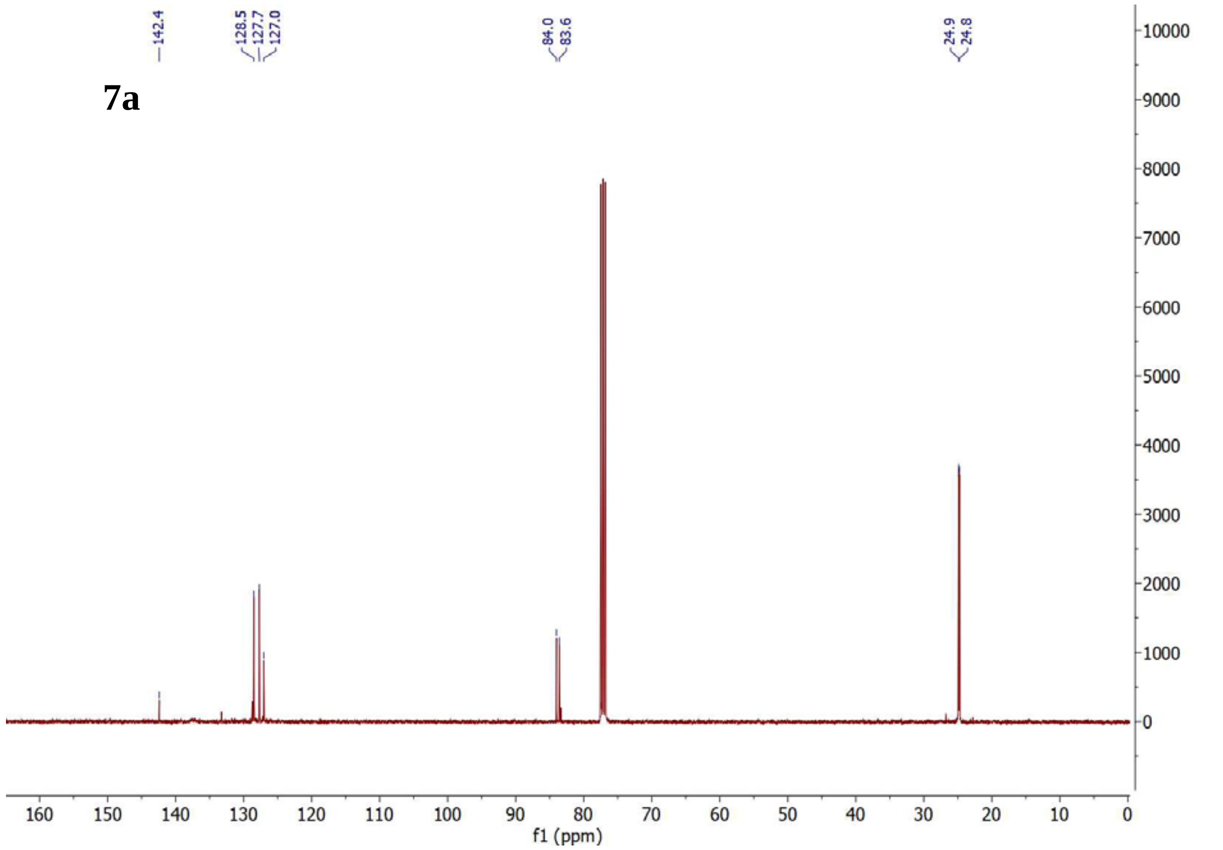

142.4
128.5
127.7
127.0
84.0
83.6
24.9
24.8
f1 (ppm)

7a

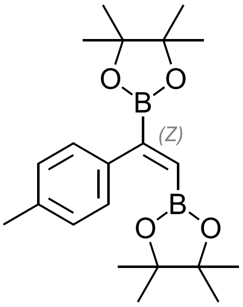
O
B
O
(Z)
B
O
O

7d

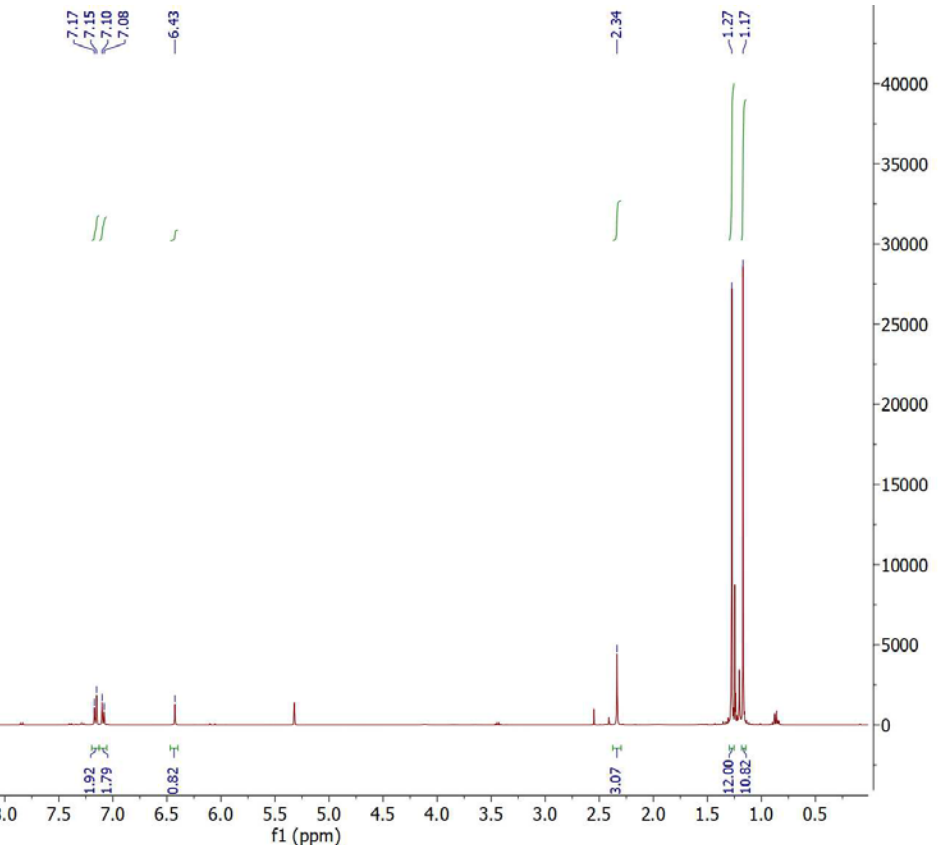
7.17
7.15
7.10
7.08
6.43
2.34
1.27
1.17
1.92
1.79
0.82
3.07
12.00
10.82
f1 (ppm)

7d

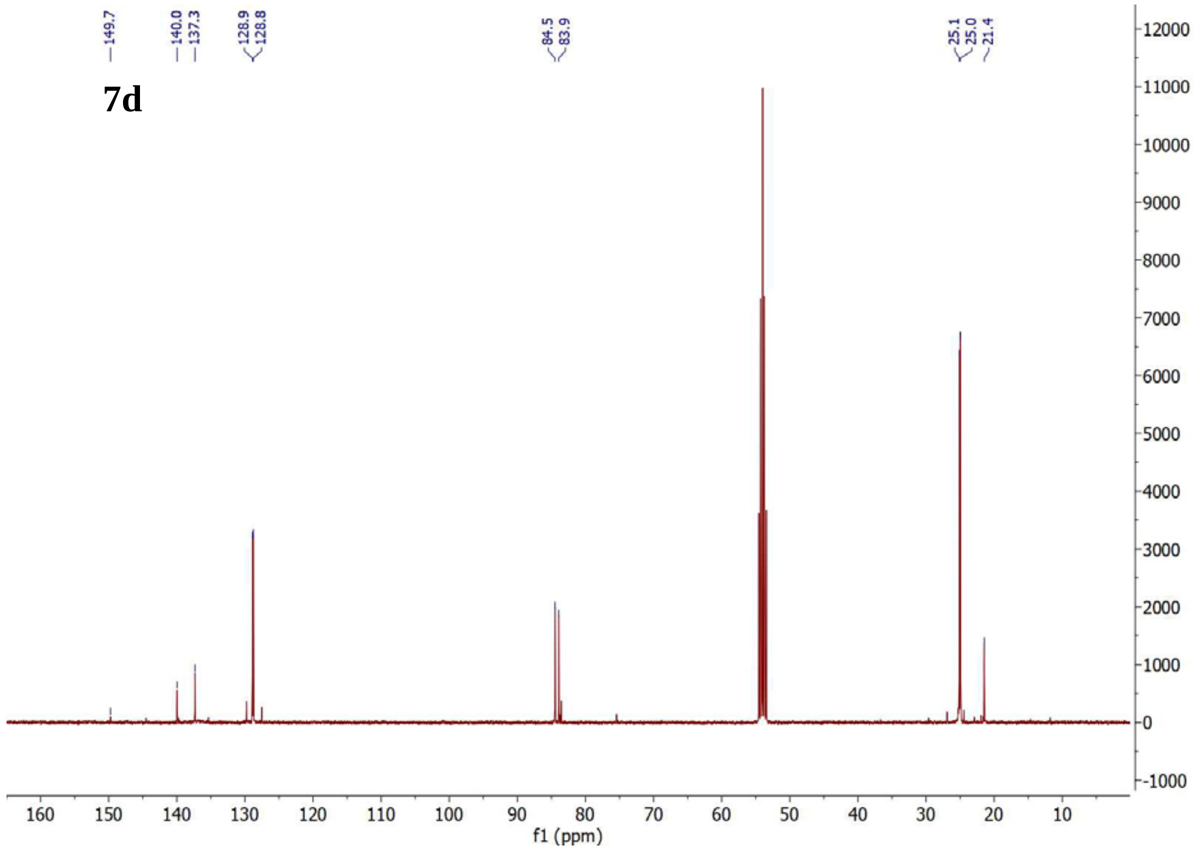
149.7
140.0
137.3
128.9
128.8
84.5
83.9
25.1
25.0
21.4
f1 (ppm)

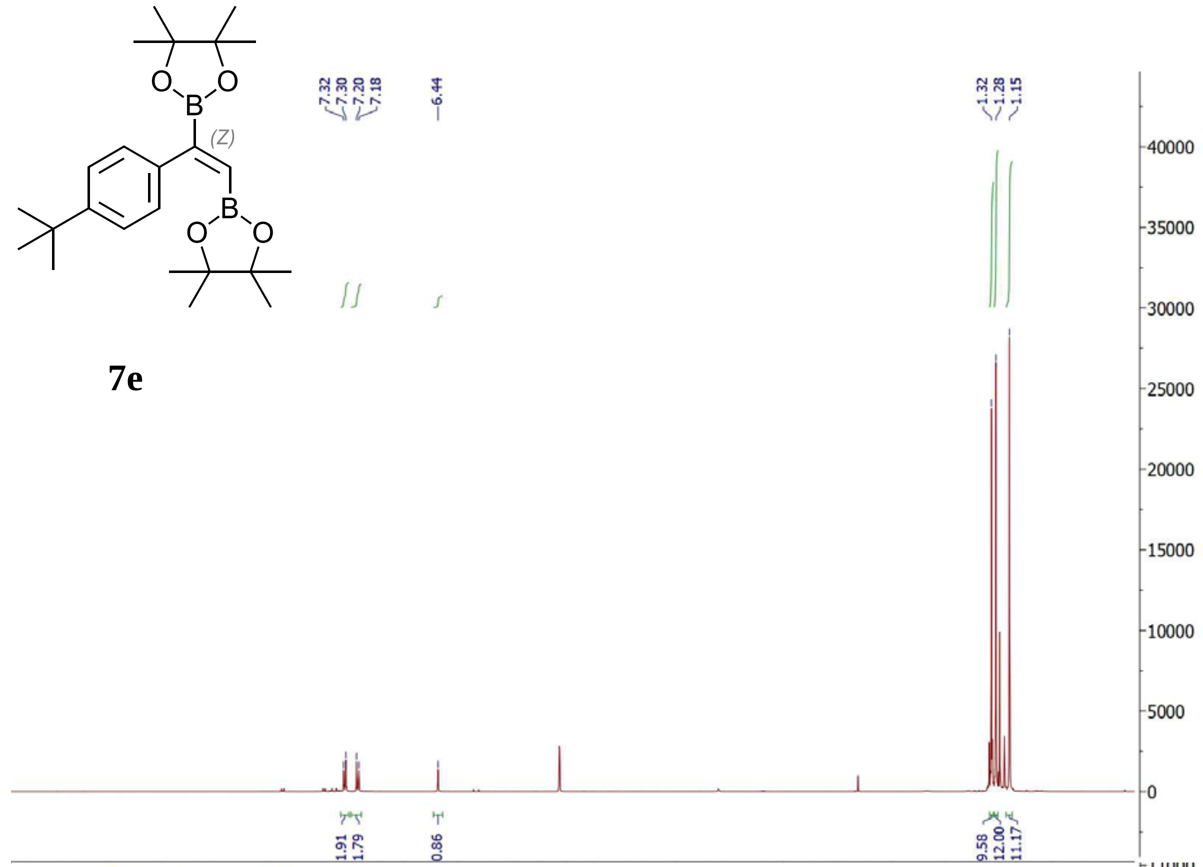
(Z)
O B O
B O O
7.32
7.30
7.20
7.18
6.44
1.32
1.28
1.15
1.91
1.79
0.86
9.58
12.00
11.17
40000
35000
30000
25000
20000
15000
10000
5000
0

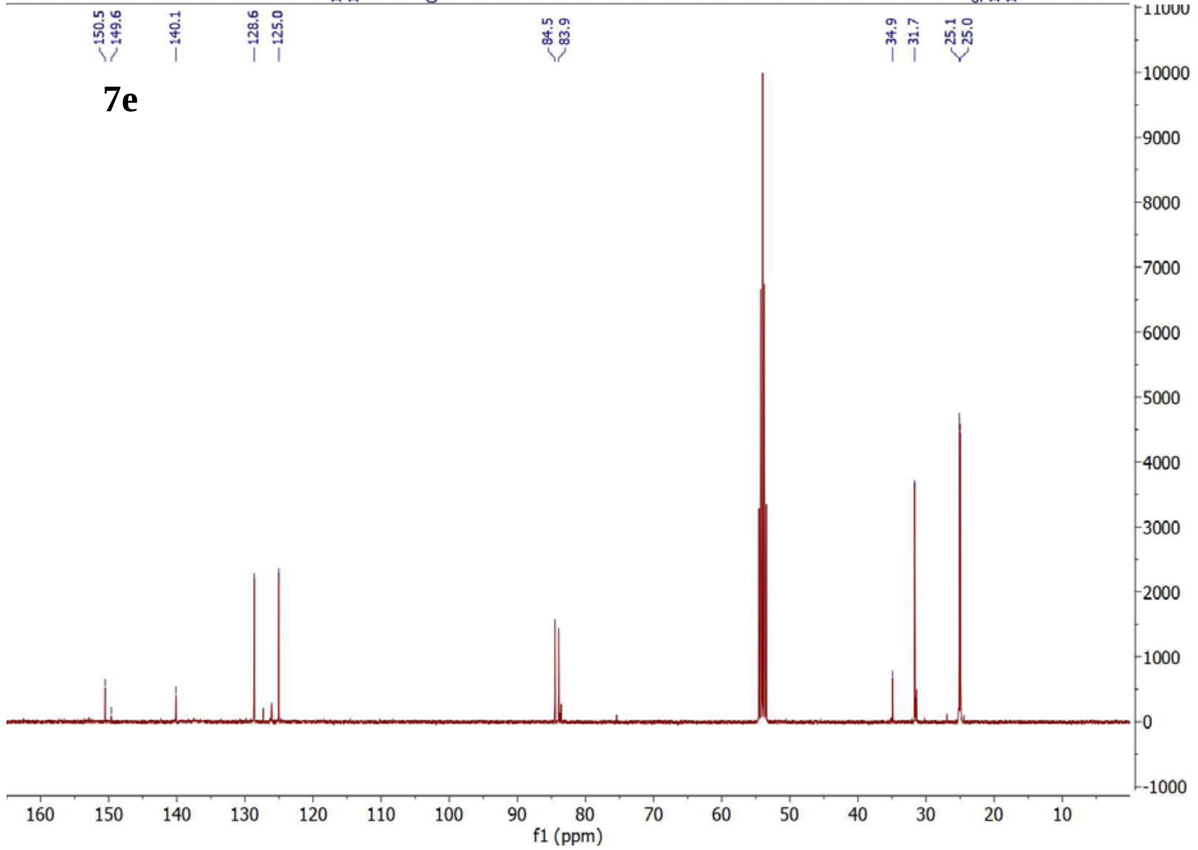
150.5
149.6
140.1
128.6
125.0
84.5
83.9
34.9
31.7
25.1
25.0
7e
11000
10000
9000
8000
7000
6000
5000
4000
3000
2000
1000
0
-1000
160 150 140 130 120 110 100 90 80 70 60 50 40 30 20 10
f1 (ppm)

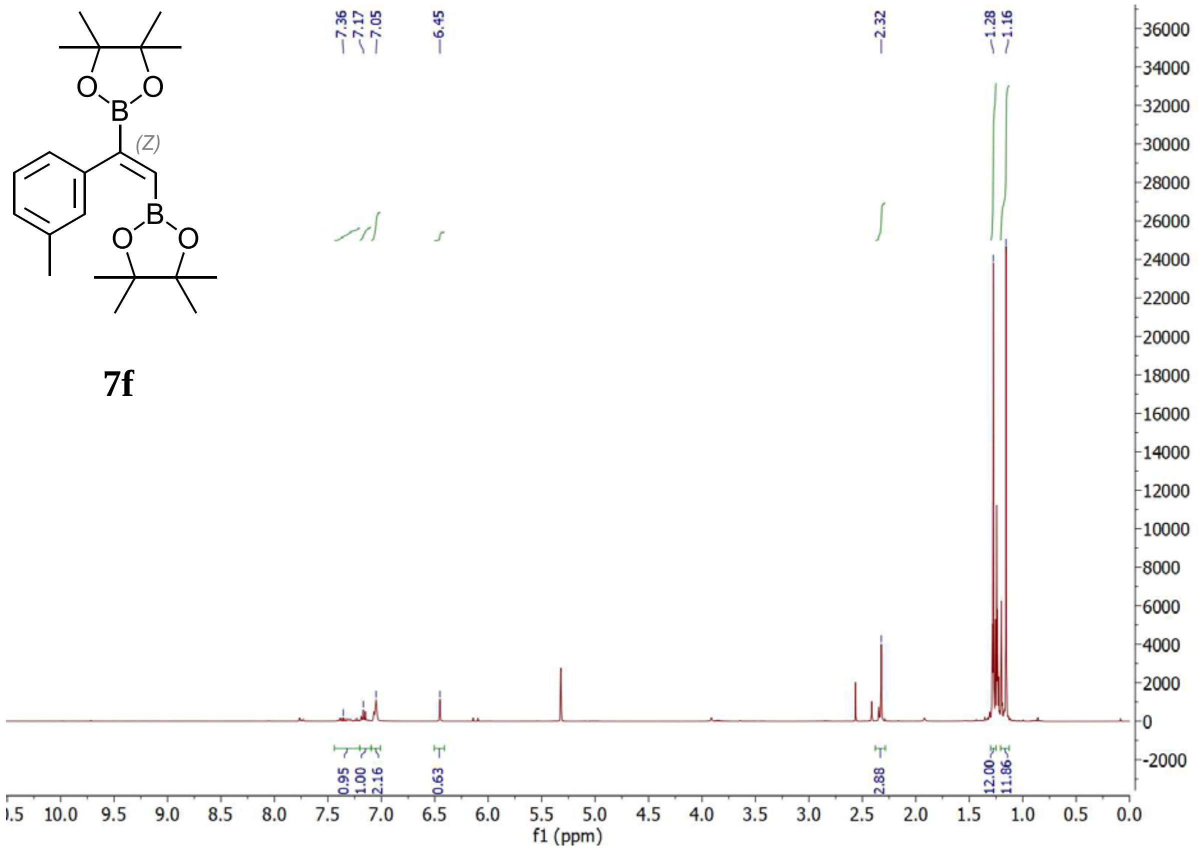
(Z)
7.36
7.17
7.05
6.45
2.32
1.28
1.16
0.95
1.00
2.16
0.63
2.88
12.00
11.86
f1 (ppm)

7f

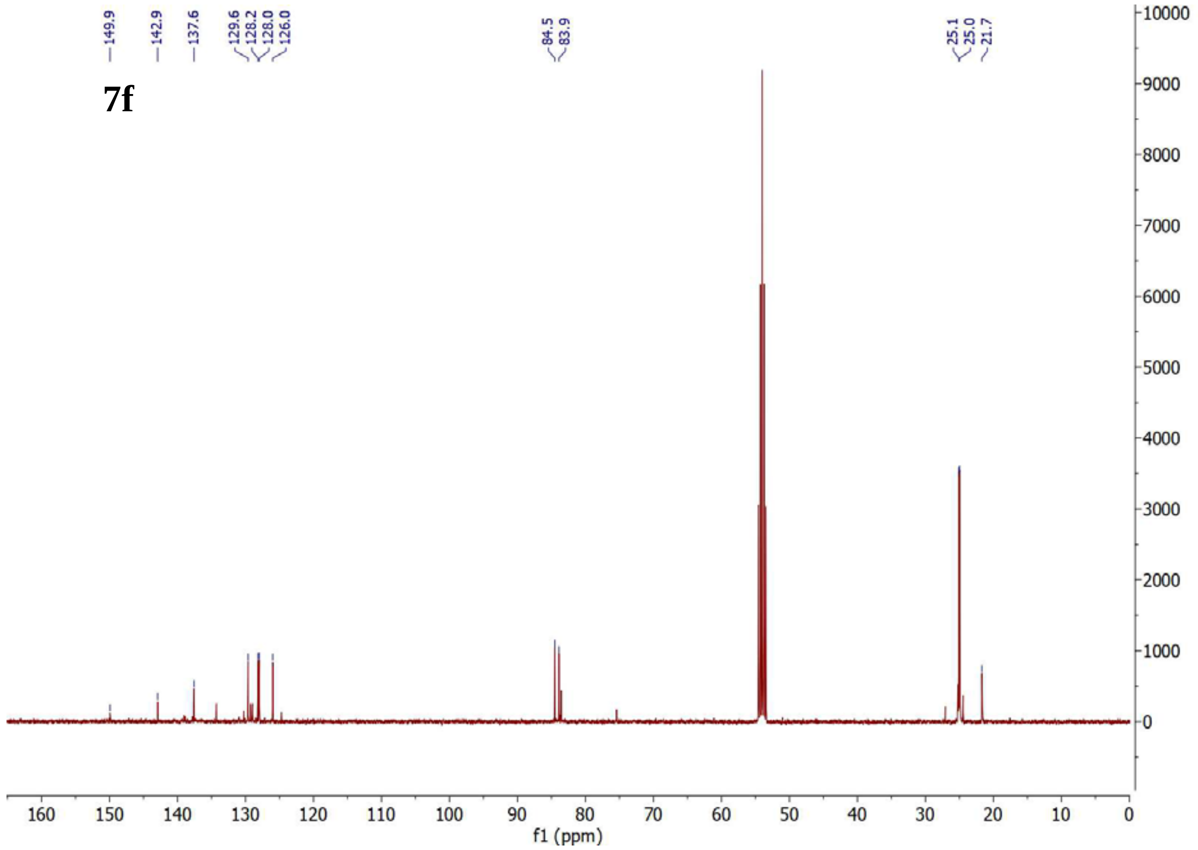
149.9
142.9
137.6
129.6
128.2
128.0
126.0
84.5
83.9
25.1
25.0
21.7
f1 (ppm)

7f

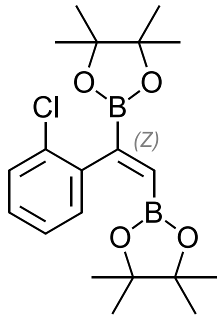
Cl
O
B
O
(Z)
O
B
O

7g

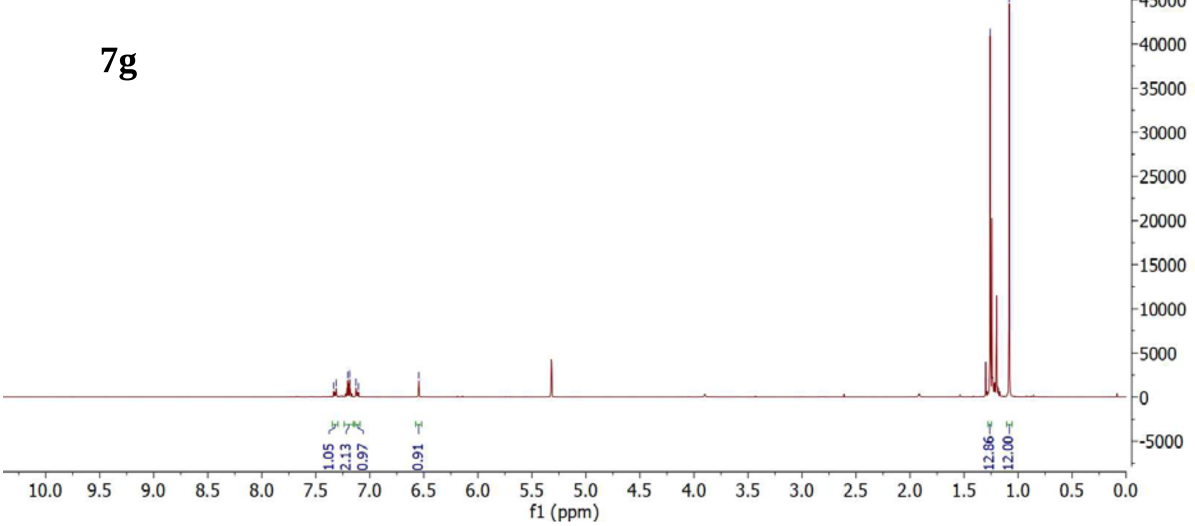
7.33
7.31
7.20
7.19
7.13
7.11
6.55
1.26
1.08
1.05
2.13
0.97
0.91
12.86
12.00
80000
75000
70000
65000
60000
55000
50000
45000
40000
35000
30000
25000
20000
15000
10000
5000
0
-5000
10.0 9.5 9.0 8.5 8.0 7.5 7.0 6.5 6.0 5.5 5.0 4.5 4.0 3.5 3.0 2.5 2.0 1.5 1.0 0.5 0.0
f1 (ppm)

7g

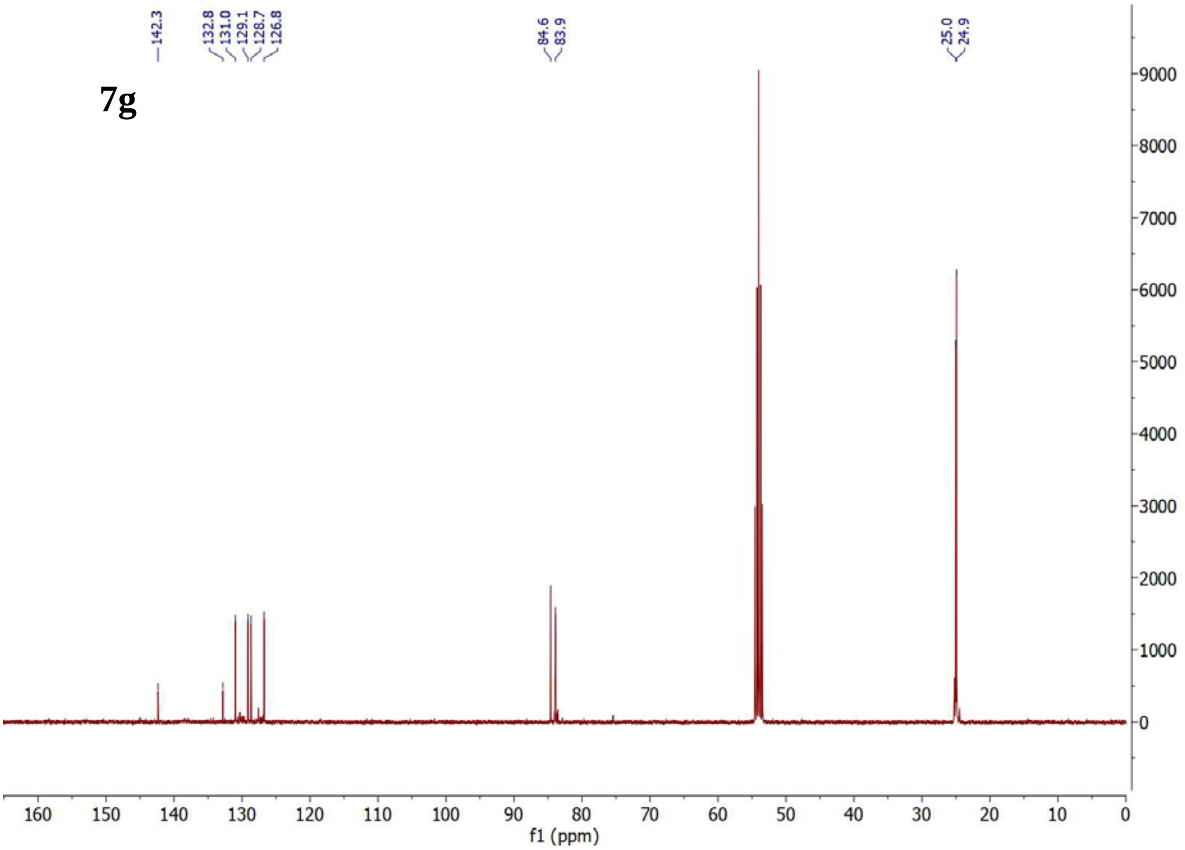
142.3
132.8
131.0
129.1
128.7
126.8
84.6
83.9
25.0
24.9
9000
8000
7000
6000
5000
4000
3000
2000
1000
0
160 150 140 130 120 110 100 90 80 70 60 50 40 30 20 10 0
f1 (ppm)

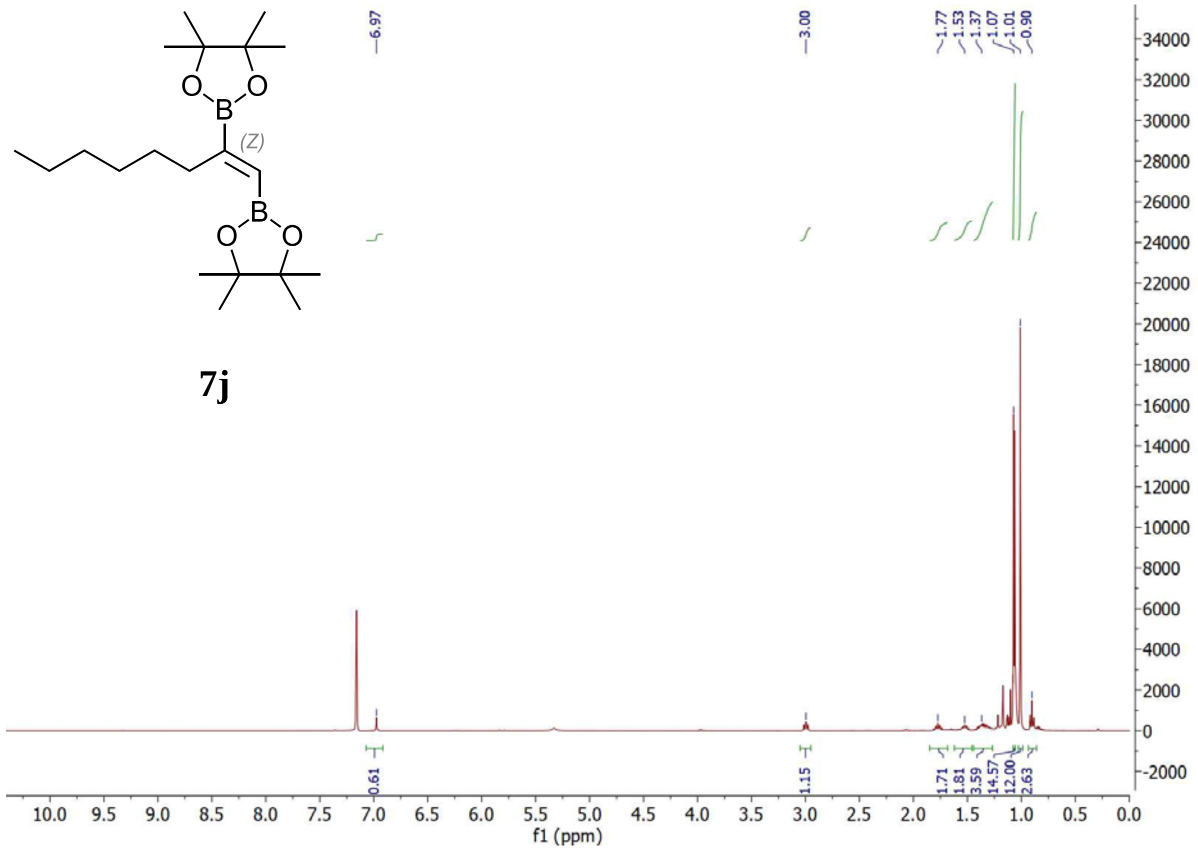
7j
(Z)
6.97
3.00
1.77
1.53
1.37
1.07
1.01
0.90
0.61
1.15
1.71
1.81
3.59
14.57
12.00
2.63
f1 (ppm)

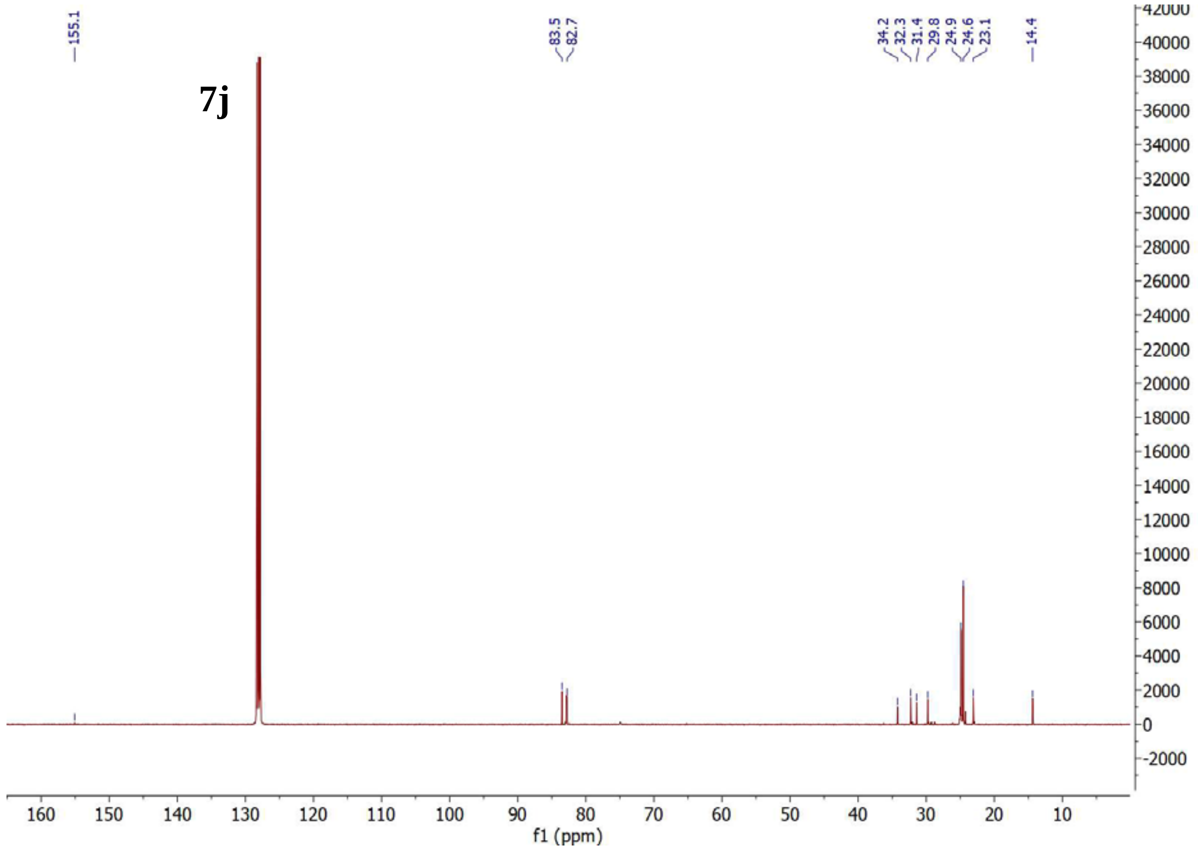
7j
155.1
83.5
82.7
34.2
32.3
31.4
29.8
24.9
24.6
23.1
14.4
f1 (ppm)

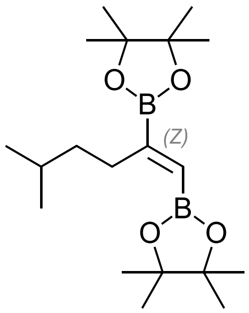
O
B
O
(Z)
B
O
O

7k

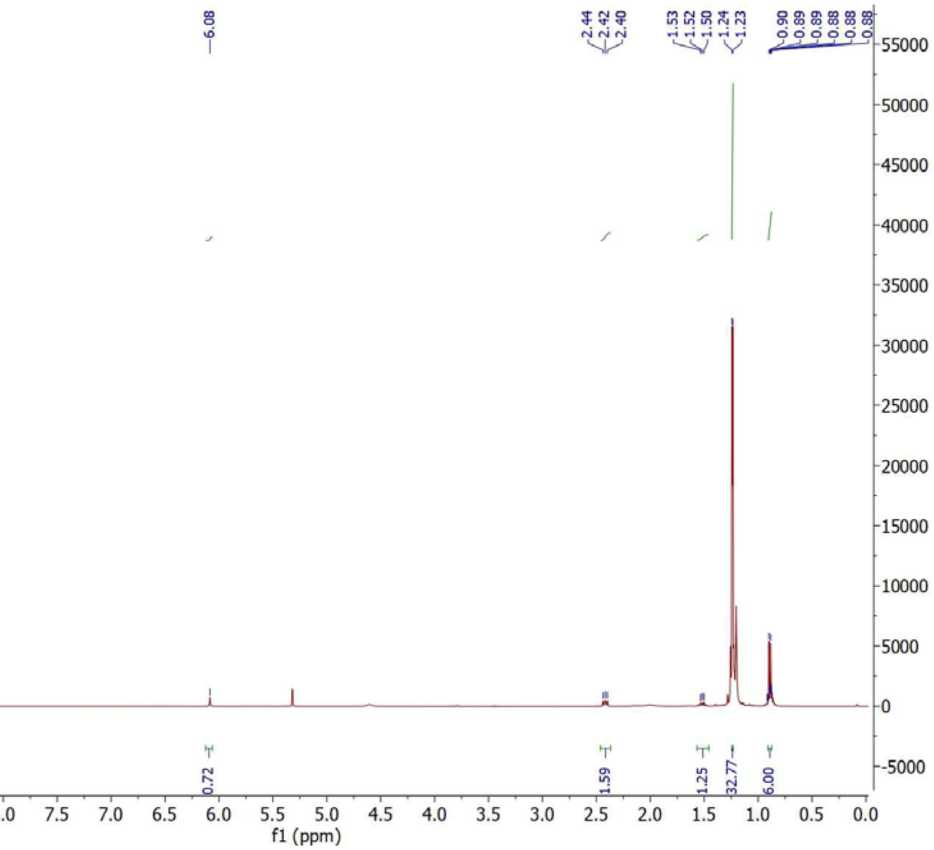
6.08
2.44
2.42
2.40
1.53
1.52
1.50
1.24
1.23
0.90
0.89
0.89
0.88
0.88
0.88
0.72
1.59
1.25
32.77
6.00
f1 (ppm)

7k

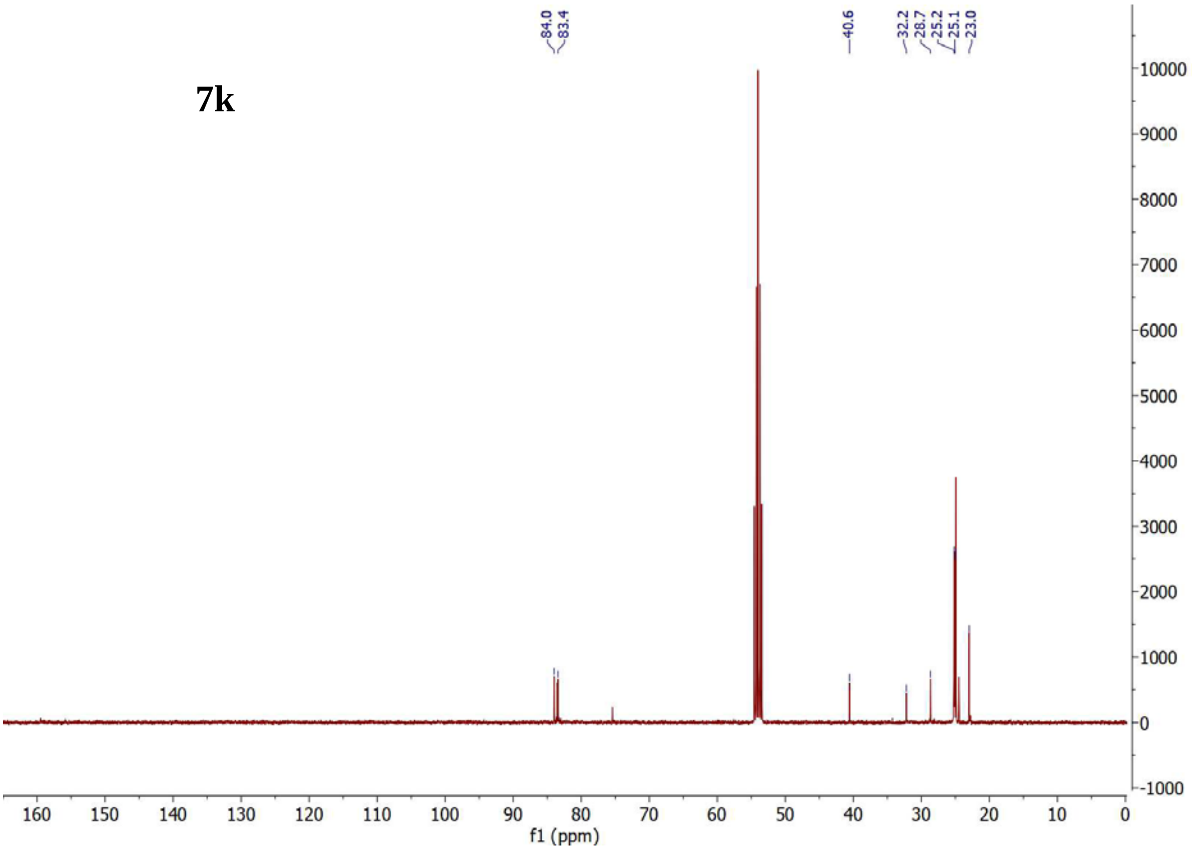
84.0
83.4
40.6
32.2
28.7
25.2
25.1
23.0
f1 (ppm)

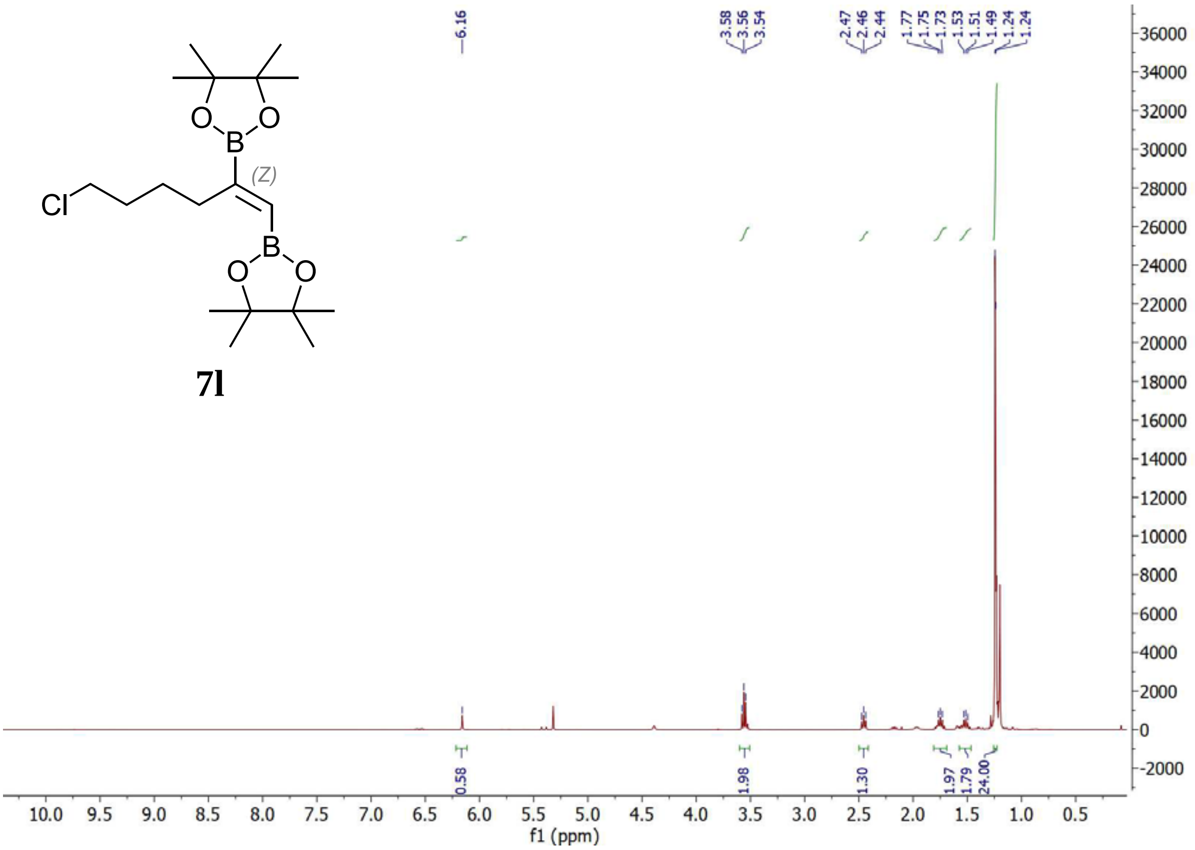
7l
Cl
O
B
(Z)
6.16
3.58
3.56
3.54
2.47
2.46
2.44
1.77
1.75
1.73
1.53
1.51
1.49
1.24
1.24
0.58
1.98
1.30
1.97
1.79
24.00
f1 (ppm)

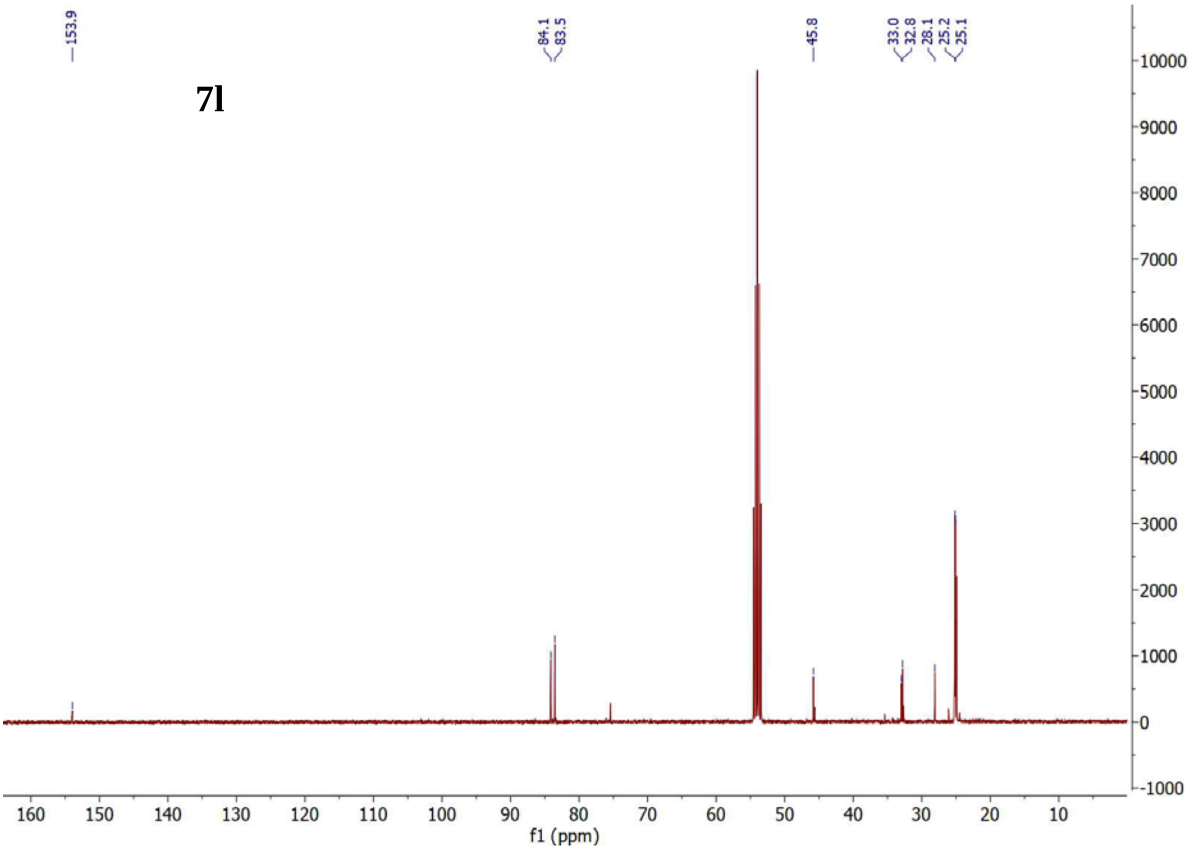
7l
153.9
84.1
83.5
45.8
33.0
32.8
28.1
25.2
25.1
f1 (ppm)

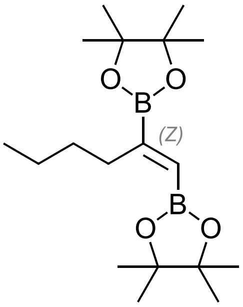
O
B
O
(Z)
O
B
O

7m

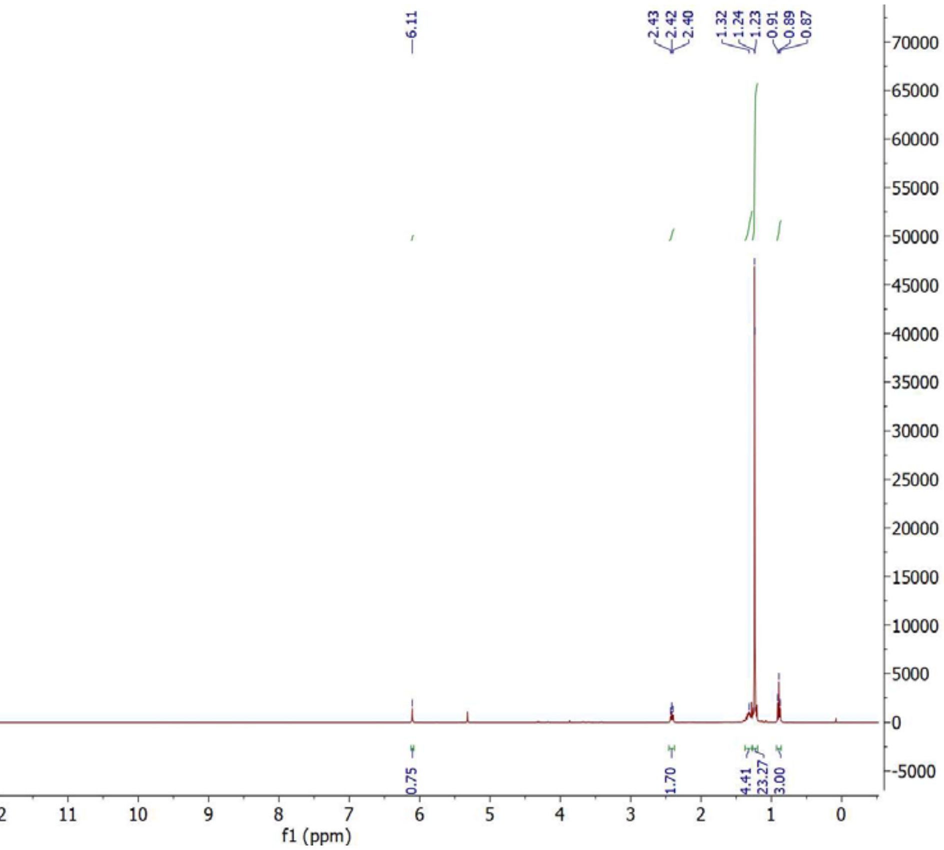
6.11
2.43
2.42
2.40
1.32
1.24
1.23
0.91
0.89
0.87
0.75
1.70
4.41
23.27
3.00
f1 (ppm)

7m

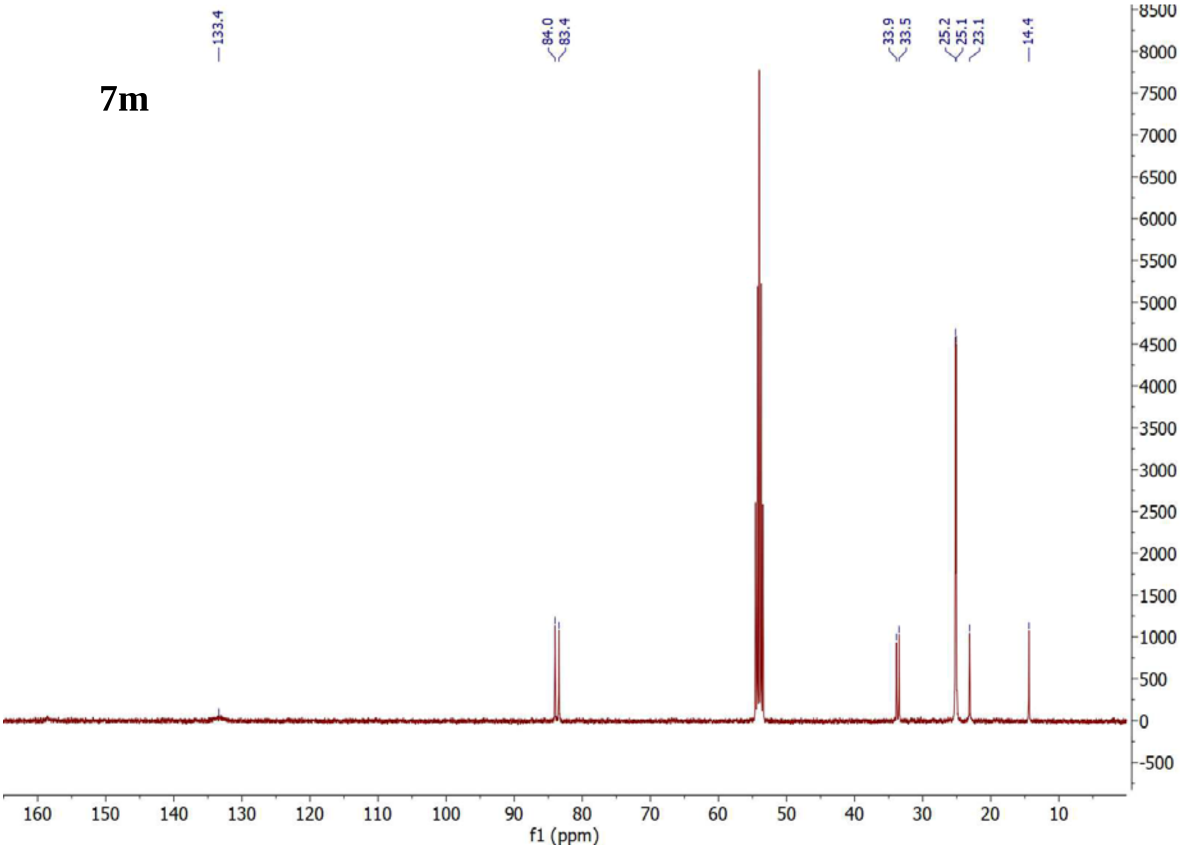
133.4
84.0
83.4
33.9
33.5
25.2
25.1
23.1
14.4
f1 (ppm)
